# Nrf2-mediated metabolic reprogramming drives regulatory T cell accumulation in hepatocellular carcinoma

**DOI:** 10.1038/s41467-026-73485-3

**Published:** 2026-07-13

**Authors:** E. Perpiñán, N. Sompairac, D. Marin Correa, E. Ramon-Gil, RCH Man, D. Camell-Raventos, R. Savoldelli, M. Fleming, L. Bellersheim, CX Santos, M. Elgosbi, JJ Lozano, M. Maddaloni, C. Schmidl, E. Kodela, M. Piqué-Gili, AH de Sande, R. Pinyol, S. Higginbotham, Q. Peng, E. Landman, J. Torres-Yaguana, V. Sharma, C. Zhang, A. Kurt, X. Liao, T. Dowe, M. Habarwaa, Z. Liu, Y. Zen, R. Miquel, D. Sarker, S. Kordasti, T. Tree, AM Shah, GO Fruhwirth, AT Dinkova-Kostova, EL Pearce, J. Leslie, JM Llovet, A. Sánchez-Fueyo, N. Safinia

**Affiliations:** 1 Roger Williams Institute of Liver Studies, School of Immunology & Microbial Sciences, Faculty of Life Sciences and Medicine, King’s College London University, and King’s College Hospital, London, UK; 2 Comprehensive Cancer Centre, School of Cancer and Pharmaceutical Sciences, Faculty of Life Sciences and Medicine, King’s College London, London, UK; 3 Haematology Department, Guy’s Hospital, London, UK; 4 Newcastle Fibrosis Research Group, Biosciences Institute, Faculty of Medical Sciences, Newcastle University, Newcastle Upon Tyne, UK; 5 The Newcastle University Centre for Cancer, Newcastle University, Newcastle upon Tyne, UK; 6 Imaging Therapies and Cancer Group, Comprehensive Cancer Centre, School of Cancer and Pharmaceutical Sciences, King’s College London, London, UK; 7 Liver Cancer Translational Research Group, Institut d’Investigacions Biomèdiques August Pi i Sunyer (IDIBAPS), Liver Unit, Hospital Clínic, Universitat de Barcelona, Barcelona, Catalonia Spain; 8 King’s College London British Heart Foundation Centre, School of Cardiovascular & Metabolic Medicine and Sciences, London, UK; 9 CIBER of Hepatic and Digestive Diseases (CIBERehd), Barcelona, Spain; 10 Leibniz Institute for Immunotherapy (LIT), Regensburg, Germany; 11 Department of Protein Science, Science for Life Laboratory, KTH-Royal Institute of Technology, Stockholm, Sweden; 12 Department of Medical Oncology, King’s College Hospital, London, UK; 13 Peter Gorer Department of Immunobiology, King’s College London, London, UK; 14 Jacqui Wood Cancer Centre, Division of Cancer Research, School of Medicine, University of Dundee, Dundee, UK; 15 Bloomberg Kimmel Institute of Cancer Immunotherapy, Department of Oncology, Johns Hopkins University School of Medicine, Baltimore, MD USA; 16 Department of Biochemistry and Molecular Biology, Johns Hopkins Bloomberg School of Public Health, Baltimore, MD USA; 17 Mount Sinai Liver Cancer Program, Division of Liver Diseases, Department of Medicine, Tisch Cancer Institute, Icahn School of Medicine at Mount Sinai, New York, NY USA; 18 Institució Catalana de Recerca i Estudis Avançats (ICREA), Barcelona, Spain

**Keywords:** Lymphocyte activation, Tumour immunology, Regulatory T cells

## Abstract

Excessive recruitment and/or activation of regulatory T cells (Treg) into the tumour microenvironment (TME) hamper anti-cancer immunity. Targeting Tregs is therefore a promising strategy to reverse the immunosuppressive features of the TME. Here, we investigate how the development of hepatocellular carcinoma (HCC) impacts the molecular programmes of tissue-resident Tregs. Tregs residing in non-tumoral liver are metabolically inert and prone to apoptosis. Conversely, HCC-infiltrating Tregs activate the nuclear factor erythroid 2-related factor-2 (Nrf2) pathway in response to the lactate-rich TME, which couples redox homeostasis with mitochondrial function and promotes Treg metabolic activity, survival, and suppressive function. Nrf2 loss of function, through either Treg-specific Nfe2l2 ablation or systemic pharmacological inhibition, prevents intra-tumoral Treg accumulation and suppresses cancer growth. Furthermore, patients with advanced HCCs enriched in Tregs with high Nrf2 activation exhibit shorter progression-free survival following atezolizumab/bevacizumab treatment. We propose Nrf2 as a target to disrupt Treg metabolic adaptation within the TME, tipping the balance between effector and regulatory immune cells and reducing cancer progression.

## Abstract (web summary)

Regulatory T cells are instrumental to the maintenance of an immune suppressive tumour microenvironment in many cancer types. Here authors show that in the hepatocellular carcinoma lactate-rich milieu, regulatory T cells acquire a more immune suppressive phenotype via upregulating Nrf2, which pathway could be targeted for immunotherapy.

## Introduction

Regulatory T cells (Treg) constitute a specialised immune subset dedicated to curbing excessive immune activation, maintaining immune homeostasis, and facilitating tissue repair^1^. In most solid tumours, Tregs are found at high frequencies, contributing to the immunosuppressive milieu of the tumour microenvironment (TME) and representing a major barrier to anti-tumour immunity^2,3^. This is particularly relevant to hepatocellular carcinoma (HCC), the third leading cause of cancer-related deaths worldwide, in which Treg infiltration is negatively associated with patient survival^4–6^.

Recent evidence highlights that the survival, lineage stability, proliferation, and suppressive function of Tregs hinge on their metabolic and nutrient-sensing machineries^7–10^. Intra-tumoral Tregs, in particular, rapidly re-wire their biosynthetic and bioenergetic metabolism to expand and persist within the TME^11–13^. It is also well-established that under non-inflammatory conditions, Tregs can traffic to healthy tissues, where they adapt to the local microenvironment by modifying their molecular and/or metabolic programs and provide specialised functions that contribute to tissue homeostasis^14–19^. Tissue-resident Tregs have been characterised in human skin, bone marrow, adipose tissue and gut, but remain to be adequately examined in other compartments. The human liver provides a unique opportunity to study how the transcriptional and metabolic adaptations of tissue-resident Tregs transition from health to disease, as the liver gradually evolves from a histologically intact organ to chronic damage and cancer.

We previously described that in patients with cirrhosis, the underlying liver pathology in most cases of HCC, circulating Tregs are reduced in number and exhibit impaired suppressive capacity and increased apoptosis. These defects are associated with dysregulation of the nuclear factor-erythroid 2-related factor 2 (Nrf2) pathway and oxidative stress-induced mitochondrial damage^20^. Nrf2 is a transcription factor that acts as a master regulator of antioxidant responses, preserves mitochondrial integrity, and supports cellular metabolism^21–25^. Under homeostatic conditions, Nrf2 is bound to Kelch-like ECH-associated protein 1 (Keap1) and subsequently degraded via the ubiquitin-proteasome pathway^26^. This process is disrupted by reactive oxygen species, electrophiles, and stress-related molecules, which interact with the cysteine sensors of Keap1, impairing its ability to ubiquitylate Nrf2 and allowing Nrf2 stabilization and nuclear translocation^27,28^. While Nrf2 has been extensively studied in cancer cells (including HCC), where it is often overexpressed and provides a survival advantage^29–31^, its role in controlling Treg redox balance and metabolism remains unexplored.

Here, we undertook a comprehensive transcriptional, metabolic, and functional analysis of circulating and intra-hepatic Tregs. Our objectives were to identify the key determinants of Treg liver tissue residency, investigate how these processes are impacted by HCC development, and elucidate the contribution of Treg Nrf2 signalling to the TME and to cancer progression. We show that Nrf2 functions as a critical metabolic checkpoint that differentially governs Treg versus effector T cell survival in HCC, with lactate-induced Nrf2 activation conferring a selective survival advantage to Tregs under metabolic stress. Genetic or pharmacological Nrf2 disruption reduces Treg numbers within tumours while preserving effector function, resulting in marked decrease in tumour growth. The therapeutic relevance of this mechanism is supported by the finding that patients with elevated intra-tumoral Nrf2-activated Tregs exhibit reduced progression-free survival following immune checkpoint inhibitors.

## Results

### Tregs in non-tumoral liver tissue are reduced in number and prone to apoptosis, while those accumulating in HCC are viable and highly suppressive

**Fig. 1:**
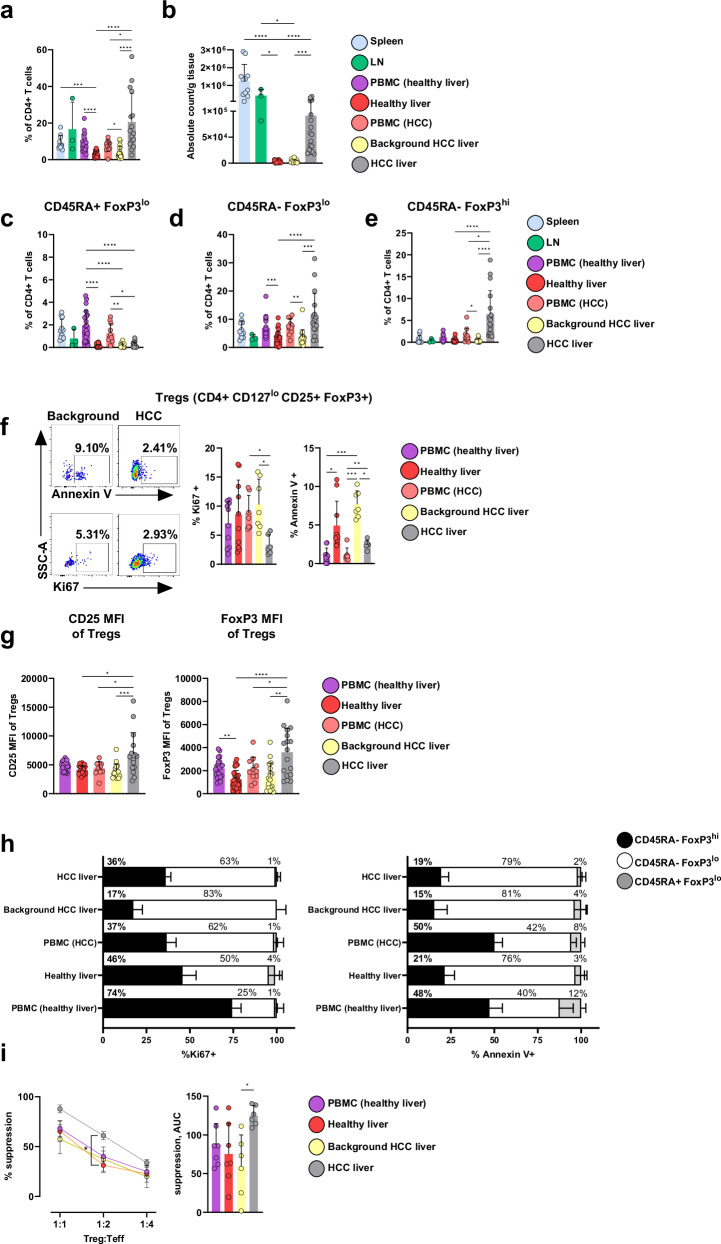
Tregs are enriched in the HCC, do not undergo proliferation or apoptosis and retain their suppressive capacity. **a** Frequency of Tregs within CD4+ T cells in different compartments: spleen and lymph nodes (LN) of healthy donors, PBMCs and paired healthy liver, and HCC and paired background HCC tissues and PBMCs. **b** Absolute number of Tregs per gram of tissue in the different liver specimens. **c**–**e** Frequencies of CD45RA+FoxP3^lo^, CD45RA-FoxP3^lo^ and CD45RA-FoxP3^hi^ within CD4+ T cells in different compartments. **f** Representative gating in an HCC patient (left) and frequencies (right) of Annexin V+ and Ki67+ cells within Tregs in different compartments. **g** Mean fluorescent intensity (MFI) of CD25 and FoxP3, respectively, in Tregs on different compartments. **h** Frequencies of Ki67+ (left) and Annexin V+ (right) cells within CD45RA-FoxP3^hi^ (black), CD45RA-FoxP3^lo^ (white) and CD45RA+FoxP3^lo^ (grey) cells in the different compartments. **i** Percentage of Treg suppression in an autologous system for each Treg:Teff ratio (left), and Treg suppressive capacity calculated by integrating the percentage of suppression for each ratio in different compartments (right). *P*-value was calculated by one-way ANOVA with Tukey’s multiple comparisons test. Data are presented as mean values + SD or ±SD as appropriate. Only statistically significant *P*-values are displayed: *, *P* < 0.05; **, *P* < 0.01; ***, *P* < 0.001; ****, *P* < 0.0001. AUC area under the curve.

To assess how Tregs adapt to a range of different microenvironments, we isolated leukocytes from human liver tissue specimens (histologically healthy liver, HCC and paired non-tumoral background HCC liver), blood, and secondary lymphoid tissues (spleen and intra-abdominal lymph nodes), and used flow cytometry to enumerate Treg subsets and characterise their homeostatic features (Supplementary Fig. 1). Healthy liver and background HCC liver specimens contained fewer Tregs than either blood or spleen (Fig. 1a and Supplementary Fig. 1). Conversely, the proportion of Tregs among HCC tumour-infiltrating lymphocytes (TIL) was higher than in blood and other liver tissue compartments (Fig. 1a–e). We next evaluated whether changes in Treg proliferation (Ki67) and/or apoptosis (Annexin V) could explain the differences in Treg numbers. Tregs from non-tumoral liver tissue were more apoptotic than circulating Tregs (Fig. 1f). On the other hand, TIL Tregs exhibited the lowest proliferation rate of all tissue specimens and reduced apoptosis compared to Tregs from paired background HCC tissue (Fig. 1f). Given the essential role of IL-2 in promoting Treg survival, we next compared the expression of the IL-2 receptor CD25 and the Treg lineage-specifying transcription factor FoxP3 in Tregs from different compartments. Tregs from healthy liver tissue expressed similar CD25 but lower FoxP3 protein levels than circulating Tregs, while TIL Tregs displayed the highest levels of both markers (Fig. 1g). In keeping with previous reports^32^, CD45RA-FoxP3^hi^ Tregs were the most highly proliferative and apoptotic Treg subset in the circulation. In contrast, among TIL Tregs CD45RA-FoxP3^hi^ Tregs were less apoptotic and proliferative than CD45RA-FoxP3^lo^ Tregs (Fig. 1h). The reduced FoxP3 protein expression and inverse correlation between activation status and apoptosis suggest that liver-resident Tregs could be competing for limiting amounts of IL-2.

Functionally, TIL Tregs showed stronger in vitro suppressive capacity than Tregs from the paired background HCC tissue, effectively restraining the proliferation of both autologous (Fig. 1i) and third-party effector CD4+ T cells (Supplementary Fig. 2a, b).

Of note, although liver tissue samples also contained CD8+FoxP3+ T cells, which were present at higher frequency than in blood, they represented a small percentage of the overall FoxP3^+^ T cell compartment. In contrast to what has been described for other tumours^33^, the number of CD8+FoxP3+ T cells in HCC samples was lower than in the paired background HCC tissue (Supplementary Fig. 2c).

### The transcriptional features of human intra-hepatic Tregs differ from other tissue-resident Treg populations

To better understand the origin of intra-hepatic Tregs and the mechanisms regulating their homeostasis, we compared Tregs and conventional CD4+ T cells (Tconv) from blood and paired healthy liver tissue specimens (*n* = 5 each) using single cell RNA-sequencing (scRNA-seq) (Supplementary Fig. 3a). Cells were grouped into 18 distinct clusters using differential gene expression (Supplementary Fig. 3b, c) and the identity of each cluster defined based on their transcriptome^14,34–39^ (Supplementary Data 1 and Supplementary Table 1). Clusters 3, 8, 11, 13 and 15 corresponded to Tregs, while the remaining clusters were classified as Tconv subsets (Supplementary Data 2 and Supplementary Table 2). The use of CD127 and CD25 surface protein expression to classify cells as Tregs yielded similar results, and few cells contained in Tconv zones were identified as CD127^lo/-^CD25^hi^ (these cells, previously referred to as “furtive” Tregs^40^, were only detected in blood and not in the liver compartment (Supplementary Fig. 3d). Furthermore, T cell receptor (TCR) clonotype analysis revealed that clusters 8 and 15, predominantly comprising circulating Tregs, exhibited shared clonotypes with Tconv clusters (clusters 2, 7, 9, and 10), whereas the remaining Treg clusters displayed either minimal or no repertoire overlap with Tconv clusters (Supplementary Fig. 4 and Supplementary Data 3). Notably, the TCR repertoire of the liver-resident Treg (cluster 11) overlapped with the repertoire of other clusters containing circulating Tregs (clusters 3 and 8) but not with Tconv clusters (Supplementary Fig. 5). These findings suggest that the reduced proportion of Tregs observed in the liver is unlikely to be due to their de-differentiation into Tconv.

Next, we investigated the key features of liver tissue residency by performing a pseudo-bulk analysis comparing the transcriptome of liver and circulating Tregs (Supplementary Data 4). Among the top differentially expressed 25 genes we found: members of the NR4A family (*NR4A1, NR4A2, NR4A3*), tissue-residency markers (*CD69, CD177*), protein G1 regulators (*RGS1, RGS2*), and genes involved in the function of activated Tregs (*DUSP2, SERPINE2, PHLDA1*). Furthermore, intra-hepatic Tregs exhibited increased transcript levels of activated/effector markers such as *BATF*, *GATA3*, *MAF*, *PRDM1* (BLIMP1), *FOXP3*, *CTLA4*, *ICOS*, *TNFRSF9* (41-BB), and *TNFRSF18* (GITR), which is consistent with what has been described for skin and adipose tissue-resident Tregs. However, in contrast to skin Tregs^41^, liver-resident Tregs did not express *ARG2*, and differed from adipose tissue Tregs in the lack of up-regulation of lipid metabolism regulators such as *PPARG*^42^, *DGAT1*, *DGAT2* or *CD36*^43^. Furthermore, unlike gut Tregs, liver-resident Tregs lacked detectable *RORc*, *RORa*, and *IL10* expression. *IL1rL*1 (ST2) expression, one of the key transcriptional features of murine tissue-resident Tregs, was not detected in liver Tregs, in keeping with what has been observed in human adipose tissue^42^. Chemokine receptor analysis revealed increased expression of *CXCR3*, *CXCR6* and *CXCR4*, and decreased expression of *CCR7*, *CCR4*, *ITGB7*, and *CCR10*.

Gene Set Enrichment Analysis (GSEA) identified “TNF-α signalling via NFκβ” as the Hallmark pathway most highly enriched in the liver Treg transcriptome. Additional gene sets over-represented in liver Tregs corresponded to pathways activated by TCR engagement (Myc targets, glycolysis), TGF-β signalling, IL-2/STAT5, and IL-6/JAK/STAT3, all of which are involved in the maintenance of Treg function. Of note, the mTOR signalling pathway, whose downregulation is a key component of the tissue adaptation of skin-resident Tregs^41^, was significantly enriched in intra-hepatic Tregs. In addition, among the top-ranked pathways we also identified those related to apoptosis and DNA damage (p53 pathway and KRAS signalling; Supplementary Fig. 3e), in agreement with the reduced viability observed by flow cytometry.

Overall, our findings reveal that, under steady-state conditions, intra-hepatic Tregs share some of the characteristic tissue-resident markers described in Tregs isolated from other tissue compartments, most notably the activation of TNF-α/NFκβ signalling^14^, but also display pathways that are distinct and may account for their reduced viability and numbers.

### Under steady-state conditions intra-hepatic Tregs exhibit two distinct programs of tissue adaptation

**Fig. 2:**
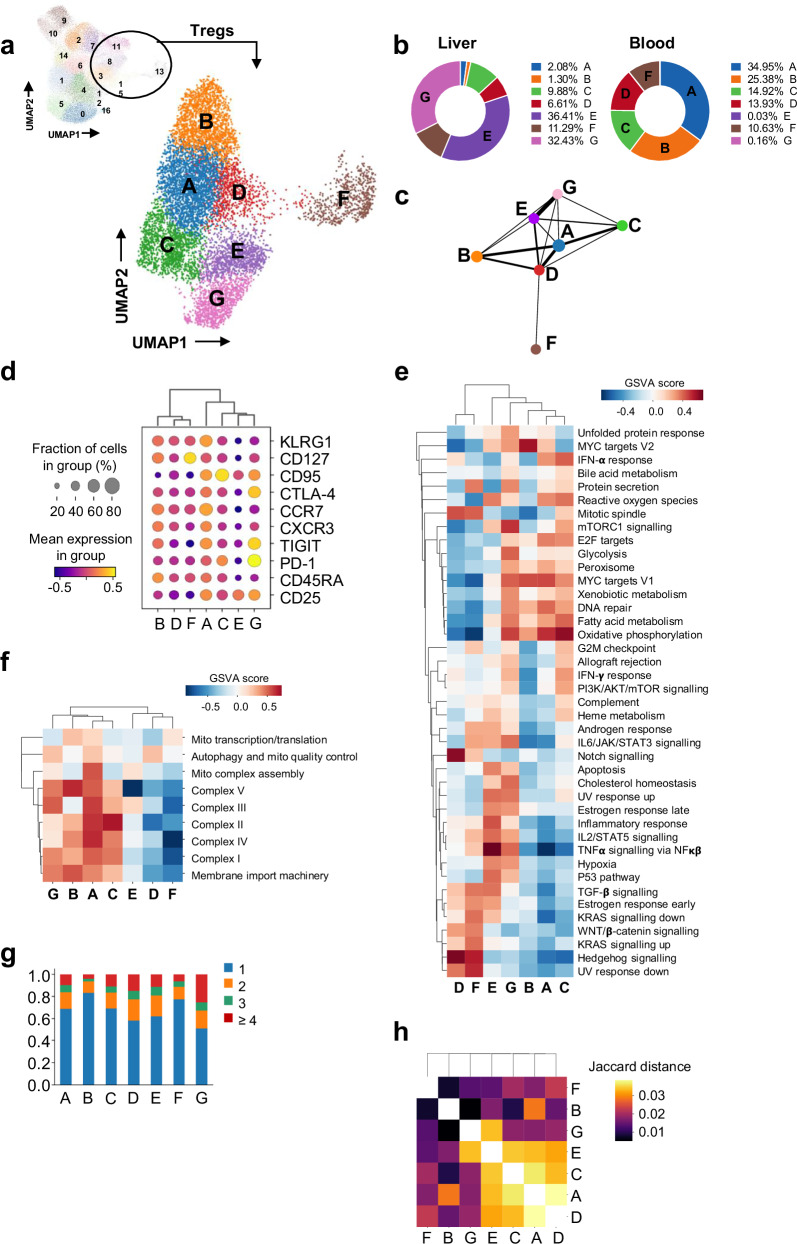
Comprehensive profiling of Treg subsets in liver and blood. **a** UMAP visualization of transcriptomes from Treg clusters 3, 8, 11, 13, and 15, identified from CD4+ T cells in healthy liver and paired PBMCs. **b** Cluster distribution across liver and blood compartments. **c** Partition graph abstraction analysis shows cluster connectivity, with thicker edges indicating stronger links and node size representing cell numbers. **d** Dot plot of surface protein expression in Treg clusters using scRNA-seq CITE-seq, with circle size proportional to marker expression frequency and color scale showing average expression levels (purple: low, yellow: high). Hierarchical clustering groups clusters based on similar protein profiles. **e**, **f** Heatmaps display GSVA enrichment scores of immune-related pathways and mitochondrial features for each cluster, with colors ranging from low (blue) to high (red). Hierarchical clustering organizes clusters and pathways by similarity. **g** Stacked bar plot represents TCR clonal expansion, showing normalized cluster size and clonotype categories: singleton (blue), 2 cells (orange), 3 cells (green), and ≥4 cells (red). **h** Heatmap demonstrates TCR repertoire overlap between clusters, with Jaccard distances visualized by color intensity (darker: lower overlap; lighter: higher overlap).

To further explore the heterogeneity of Tregs in blood and healthy liver tissue, we performed a sub-cluster analysis that yielded 7 Treg clusters (A to G). Clusters E and G were exclusive to liver tissue, clusters A and B mainly corresponded to circulating Tregs, and clusters C, D, and F contained Tregs from both compartments (Fig. 2a, b). We used partition-based graph abstraction (PAGA) mapping to assess the inter-cluster relationships, which showed high connectivity among the 2 liver resident clusters (E and G), and between the 4 largest clusters in blood (A–D) (Fig. 2c).

Cluster B corresponded to naïve Tregs (high expression of *CCR7* and *CD45RA*), while the rest of the clusters contained memory/activated Tregs (Supplementary Data 5). Clusters E and G, which included most liver resident Tregs, were markedly different to each other. Cluster G showed high expression of genes encoding for co-inhibitory receptors (*CTLA4* and *PDCD1*), TNF superfamily members (*TNFRSF18*, *TNFRSF4* and *TNFRSF9*), and common Treg molecules such as *FOXP3*, *CARD16*, *DUSP4* and *CD27*, as well as high levels of CD25, PD-1, TIGIT and CTLA-4 (Fig. 2d). In contrast, Tregs grouped within cluster E expressed high transcript levels of *CD69* and members of the NR4A family and low expression of PD-1, TIGIT, and CTLA-4 (Fig. 2d). Gene Set Variation Analysis (GSVA) revealed that, as compared to circulating Tregs, the transcriptomes of clusters E and G were enriched in TNF-α/NFκβ signalling, hypoxia, IL2/STAT5, IL6/JAK/STAT3 and apoptosis pathways (Fig. 2e). However, clusters E and G differed in their metabolic and signalling profiles. Cluster G demonstrated significant enrichment of TCR-mediated activation pathways, concurrent with elevated mTORC1 signalling and enhanced bioenergetic activity, as determined by the upregulation of oxidative phosphorylation, TCA cycle function, fatty acid metabolism (Fig. 2e and Supplementary Fig. 6) and mitochondrial gene expression (Fig. 2f). Conversely, cluster E displayed attenuated TCR signalling, increased markers of apoptosis, and markedly reduced metabolic activity. (Fig. 2e, f and Supplementary Fig. 6). Cluster G contained the largest number of expanded clonotypes (occurring ≥4 times) of all Treg clusters (Fig. 2g), and its repertoire substantially overlapped with cluster E, but not with circulating Tregs. Cluster E, in turn, showed repertoire overlap with clusters A, C, and D, which suggests trafficking between the blood and liver Treg compartments (Fig. 2h). To further investigate the differentiation states of intra-hepatic Tregs, we performed pseudotime trajectory analysis (Supplementary Fig. 7). We observed a clear point of divergence with two separate states of differentiation that corresponded to clusters E and G, with cluster E being closer to blood Tregs than cluster G.

Collectively, these data suggest that Tregs respond to the liver microenvironmental cues through two distinct but interconnected programs and that TCR engagement is likely to be required for their metabolic adaptation and survival.

### HCC promotes the expansion of Treg subpopulations with active metabolism and robust Nrf2 activity

**Fig. 3:**
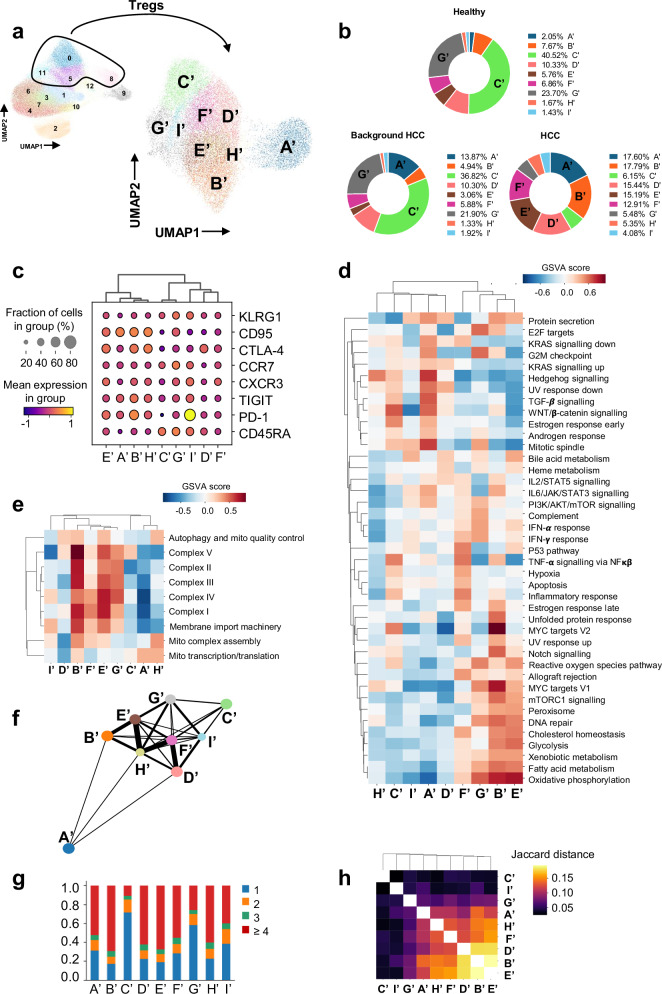
Heterogeneity of the Treg compartment in HCC. **a** UMAP projection of transcriptomes from Treg clusters 0, 5, 8, and 11, identified in CD4+ T cells from healthy liver, HCC, and paired background HCC tissues (*n* = 5 each). **b** Cluster distribution across liver compartments. **c** Dot plot of protein surface expression assessed by scRNA-seq CITE-seq, with circle size indicating expression frequency and color scale (purple: low, yellow: high) showing mean expression levels. Hierarchical clustering groups clusters with similar protein profiles. **d**, **e** Heatmaps display GSVA enrichment scores of immune-related pathways and mitochondrial features, respectively, with color ranges from low (blue) to high (red). Clusters and pathways are hierarchically grouped by similarity. **f** Partition graph abstraction analysis reveals cluster connectivity, where thicker edges show stronger links, and node size reflects cell count. **g** Stacked bar plot represents TCR clonal expansion normalized by cluster size, with clonotypes color-coded: singleton (blue), 2 cells (orange), 3 cells (green), ≥4 cells (red). **h** Heatmap shows TCR repertoire overlap between clusters, with Jaccard distances represented by color intensity (darker: lower overlap; lighter: higher overlap).

Having identified the key molecular differences between circulating and intra-hepatic Tregs under homeostatic conditions, we then investigated the impact of HCC on the molecular programmes of liver-resident Tregs. To this end, we conducted additional scRNA-seq experiments on Tregs and Tconv cells isolated from 5 HCC and 5 paired background HCC non-tumoral liver tissue specimens (which exhibited variable degrees of fibrosis/inflammation). Data was then integrated and analysed together with the healthy liver tissue scRNA-seq dataset outlined above. We identified 12 clusters, among which numbers 0, 5, 8, and 11 corresponded to Tregs and the remaining to Tconv (Supplementary Fig. 8a–e and Supplementary Tables 3 and 4). Tregs were then sub-clustered into 9 clusters (denoted by letters A’ to I’; Fig. 3a).

Clusters C’ and G’ (which highly resembled the E and G liver-resident clusters identified in our previous analysis, respectively) were the predominant Treg sub-populations in non-tumoral liver tissues (Fig. 3b). Cluster C’ was defined by the expression of the transcription factor *CRIP2*, known to downregulate NFκβ upon T cell activation^44^, and expressed central memory lymphoid tissue-associated genes (e.g. *CCR7*, *S1PR1*, *SELL* and *TCF7*^14^) and low protein levels of CD95, CTLA-4 and PD-1 (Fig. 3c and Supplementary Data 6). Functionally, cluster C’ was characterised by reduced metabolic activity and increased apoptosis, and exhibited the highest enrichment in TNF-α/NFκβ and Wnt/β-catenin signalling (Fig. 3d,e) and the lowest clonal expansion of all Treg clusters (Fig. 3g). Cluster G’, whose top differentially expressed gene was *HOPX*, previously shown to enhance peripheral Treg fitness under inflammatory conditions^45^, displayed a more activated and proliferative phenotype than cluster C’, as assessed by increased expression of suppressive/activation-related genes (*GZMA*, *GZMK, KLRG1*), proliferative markers (*MKI67, TOP2)*, and KLRG1 and CD95 proteins (Fig. 3c and Supplementary Data 6). Pathway analysis revealed that cluster G’ showed higher enrichment than cluster C’ in mTORC1 signalling, MYC targets, and essential metabolic pathways (glycolysis, oxidative phosphorylation, citrate cycle, purine and pyrimidine metabolism) (Fig. 3d and Supplementary Fig. 9a).

TIL Tregs were highly heterogeneous and assembled into 5 main clusters: A’ (18%), B’ (18%), E’ (15%), D’ (15%), and F’ (13%) (Fig. 3b). These 5 clusters showed high inter-connectivity (Fig. 3f). Furthermore, their respective TCR repertoires displayed considerable overlap and a much higher clonal expansion than Tregs residing in non-tumoral tissue, with whom they shared almost no repertoire overlap (Fig. 3g, h). Clusters B’ and E’ exhibited the highest transcript levels of TNF superfamily members (*TNFRSF1B*, *TNFRSF4* and *TNFRSF18*), and genes promoting Treg viability, stability, and function (*CD177*, *FOXP3*, *SOCS1* and *LGALS1*)^46^, as well as high protein expression of CD95, CTLA-4 and PD-1 (Fig. 3c and Supplementary Data 6). Pathway analysis indicated that these 2 clusters showed the highest degree of TCR activation and were the most metabolically active, as illustrated by their enrichment in gene sets related to mTORC1 signalling, MYC targets and a wide range of metabolic pathways (Fig. 3d, e, and Supplementary Fig. 9a).

Given that in chronic liver disease Tregs are dysfunctional and prone to apoptosis due to Nrf2 dysregulation (*20*), we hypothesized that the HCC microenvironment may promote Treg accumulation by activating Nrf2, thereby ensuring their metabolic fitness and survival. Clusters B’ and E’ showed significant enrichment in a 27-gene signature known to be regulated by Nrf2, with cluster B’ exhibiting the highest enrichment score (Supplementary Fig. 9b, c and Supplementary Table 5). To identify specific mediators promoting HCC Treg accumulation, we also performed bulk-RNA-seq and multiplex cytokine analyses on HCC and paired background tissue, but we observed no differences in growth factors known to support Treg proliferation (Supplementary Fig. 10a, b).

Finally, we investigated whether CD4+FoxP3- type 1 regulatory T (Tr1)-like cells, endowed with immunosuppressive properties and enriched in human colorectal liver metastases and non-small-cell lung cancers^47^, also contributed to the HCC TME. We interrogated the Tconv clusters using GSEA and a Tr1-like transcriptional signature. This signature was significantly enriched in cluster 2, which exhibited high expression of *EOMES*, *GZMK*, *GZMH*, *NKG7*, *CST7*, and the Tr1-like specific *CHI3L2* (Supplementary Fig. 8f). However, these cells constituted a minor fraction of the overall CD4+ T cell compartment in HCC (7.98%, as compared to 48.95% for Tregs).

Our data reveal that, although the TIL Treg compartment contains multiple clusters exhibiting a range of molecular and metabolic profiles, the largest Treg fraction corresponds to those with a highly active metabolic state, potent antioxidant activity, low apoptotic rate, and high TCR oligoclonal expansion.

### Highly metabolically active Tregs are enriched in immune exhausted HCC tumours and are associated with worse clinical outcomes

**Fig. 4:**
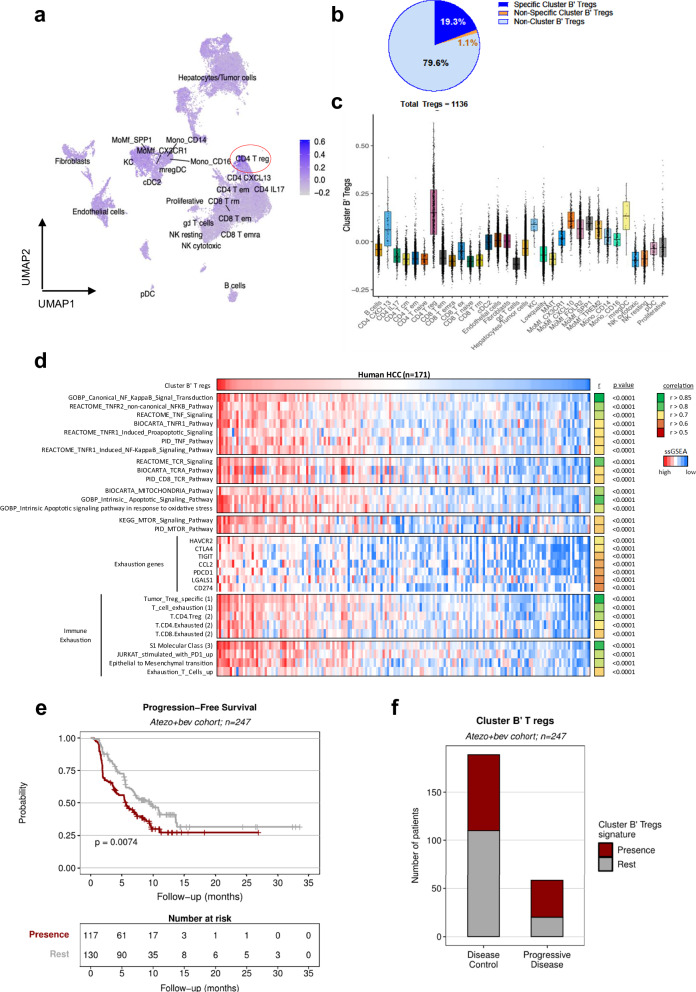
Tregs acquire intracellular molecular programs to adapt to the TME. **a** UMAP plot of scRNA-seq HCC data (GSE151530), annotated with cell-type-specific gene signatures from ref. ^99^, showing higher Cluster B’ Tregs scores in darker purple. **b** Specificity of Cluster B’ Tregs defined by gene score cut-off: specific Cluster B’ Tregs (annotated as Tregs) *vs* non-specific Cluster B’ Tregs (not annotated as Tregs but identified as Cluster B’ Tregs). **c** Box plots of Cluster B’ Tregs gene scores display cell types most representative of this signature. Box plots display the full range (minimum to maximum), with the center line indicating the mean. **d** Heatmap shows Spearman correlation between ssGSEA scores from a 64-gene Cluster B’ signature and key pathways/immune signatures from bulk RNA-seq in 171 HCC samples. **e** Kaplan Meier curve depicting progression free survival (PFS) according to the presence of the Cluster B’ Tregs signature. Enrichment in Cluster B’ transcriptional signature was significantly associated with shorter PFS [median PFS 5.65 *vs* 9.46 months; HR 1.55 (95% CI 1.12–2.15); *p* = 0.0074]. Hazard Ratio (HR), 95% Confidence Intervals (CIs) and *p*-values were calculated using a univariate Cox regression analysis. **f** Bar plot representation of the Cluster B’ Treg signature in atezo+bev-treated HCCs according to patients’ responses. The presence of Cluster B’ Treg in tumours from patients who achieved disease control (i.e. complete or partial response, or stable disease as best response) was significantly lower than in patients with progressive disease (42% *vs* 66%; *p* = 0.0026). Fisher’s exact test (two-sided).

Having identified cluster B’ as containing the Treg subset best equipped to survive in the HCC TME, we sought to determine the generalizability of our results by investigating the presence of cluster B’ Tregs within the overall immunogenomic landscape of HCC. First, we defined a 64-gene signature that differentiated cluster B’ from all other CD4^+^ T cells in our dataset (Supplementary Table 5). We confirmed the specificity of the signature by interrogating an independent, publicly available, scRNA-seq dataset containing both immune and cancer cells from 46 HCC specimens^48^. Among the Tregs identified in the independent dataset, 19% exhibited significant enrichment in the 64-gene signature (Fig. 4a–c), which closely matched the proportion of Tregs contained in cluster B’ in our study (18%). Next, we employed the signature to classify a very well characterised cohort of 171 HCC samples^49–52^. In line with the findings described above, tumours presenting high cluster B’ Treg scores exhibited significant enrichment in TNF-α/NFκβ signalling, as well as activation of TCR and mTOR pathways (Fig. 4d). This indicates that Tregs adapt to the TME by acquiring intracellular molecular programs shared by cancer cells. Furthermore, tumours with high abundance of cluster B’ Tregs were enriched in immune checkpoint molecules, gene signatures associated with exhausted immune phenotypes, and S1 molecular-class features^53^ (Fig. 4d). The use of previously defined HCC molecular and immune classifications^49,50^ revealed that cluster B’ Tregs were significantly enriched in *Inflamed HCC* tumours, with the highest enrichment score corresponding to the *Immune Exhausted* subclass, while they were not present in *Immune Excluded* tumours (Supplementary Fig. 11a). Further, cluster B’ Tregs were also enriched in S1/proliferation HCC subclasses and excluded from the non-proliferation/S3-CTNNB1 subtypes (Supplementary Fig. 11b–d).

To explore the impact of cluster B’ Tregs on clinical outcomes, we assessed their association with progression-free survival (PFS) in a cohort of 247 HCC patients treated with first-line atezolizumab/bevacizumab within two clinical trials^54–56^. Enrichment in cluster B’ transcriptional signature was associated with shorter PFS (Fig. 4e). Furthermore, the presence of cluster B’ Treg in tumours from patients who achieved disease control was lower than in patients with progressive disease (Fig. 4f). Collectively, these data suggest that the accumulation of highly activated Tregs: (i) may contribute to the immunosuppressive HCC TME by promoting T cell exhaustion; and (ii) could be implicated in the resistance to immune therapies of advanced-stage HCC.

### Lactate within the HCC microenvironment drives MCT-1 dependent Nrf2 activation in Tregs

**Fig. 5:**
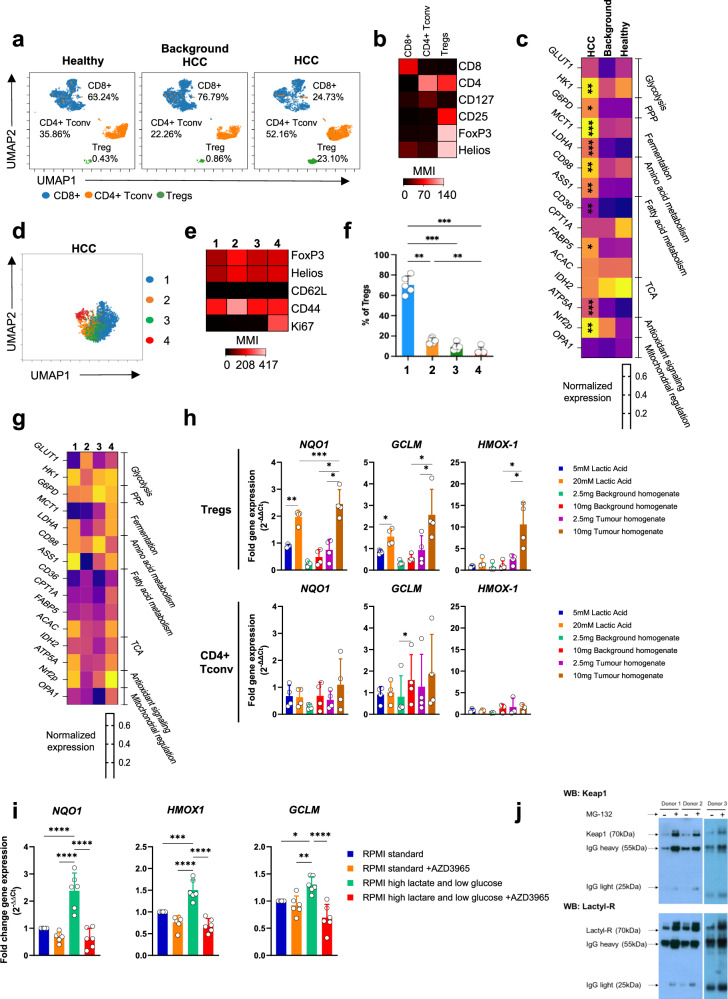
HCC Tregs display enhanced metabolic fitness and antioxidant capacity linked to lactate-induced MCT1/Nrf2 signaling. **a** UMAP plot shows three T cell lineages (Tregs, CD8+, and CD4+ Tconv) in healthy liver, HCC, and paired background HCC tissues (*n* = 5 each) using phenotypic markers (9 features). Cells are color-coded by lineage identity. **b** Heatmap displays median metal intensity (MMI) of phenotypic markers delineating the three T cell lineages. **c** Heatmap illustrates normalized expression of key metabolic regulators in Tregs from healthy, HCC, and background HCC liver tissue. Statistical significance values indicate comparisons between HCC and background Tregs. **d** UMAP plot identifies HCC-Treg populations based on metabolic feature expression. **e** Heatmap shows MMI of FoxP3, Helios, CD62L, CD44, and Ki67 in HCC Treg metabolic subsets. **f** Bar plot indicates frequency of Treg metabolic subsets in tumor-infiltrating Tregs across specimen. **g** Heatmap highlights normalized expression of metabolic regulators in HCC Treg subsets. **h**, **i** Fold expression of Nrf2-target genes is assessed by qPCR in human Tregs exposed to lactate, HCC and paired background HCC tissue homogenates, or MCT1 inhibition under defined conditions. **j** Representative western blot (WB) depicts Keap1 lactylation in Tregs exposed to lactate with or without proteasome inhibition. Uncropped WB scans are included in the Source Data. Data are presented as mean values + SD as appropriate. *P*-values were calculated as two-way ANOVA followed by Dunnett’s multiple comparisons post-hoc test versus HCC for **c**; or one-way ANOVA followed by Tukey’s multiple comparisons post-hoc test for (**f**, **h**, **i**). Statistical significance: *, *P* < 0.05; **, *P* < 0.01; ***, *P* < 0.001; ****, *P* < 0.0001.

To further elucidate the involvement of specific metabolic pathways in the functional adaptation of intra-tumoral Tregs, we used mass cytometry to assess the expression of key metabolic enzymes/transporters in liver-resident Tregs and Tconv (CD4+ and CD8+) isolated from HCC, paired non-tumoral background liver tissue, and healthy liver (Fig. 5a, b). TIL Tregs were characterised by increased expression of rate-limiting enzymes and transporters involved in glycolysis (HK1), PPP (pentose phosphate pathway, G6PD), amino acid (CD98 and ASS1) and lactate (MCT1 and LDHA) metabolism, fatty acid uptake (CD36), and ATP synthesis (ATP5A) (Fig. 5c). Furthermore, when compared to both Tregs isolated from non-tumoral tissues and intra-tumoral CD8+/CD4+ Tconv, TIL Tregs exhibited increased levels of phosphorylated Nrf2 (Nrf2p), suggestive of‘ Nrf2 accumulation (Supplementary Fig. 12a–c). Using unsupervised clustering analysis we identified 4 metabolic subpopulations among TIL Tregs (Fig. 5d–f). Subpopulation 1 (70%) exhibited the highest levels of Nrf2p, in addition to increased expression of CD36, CPT1A, FABP5 and MCT1, whilst subpopulation 3, exhibiting the lowest expression of Nrf2p, was highly glycolytic with elevated GLUT1 expression (Fig. 5g and Supplementary Data 7). These results confirm our scRNA-seq findings and are suggestive of a link between Nrf2 activation and the engagement of metabolic pathways known to be important for Treg metabolic adaptation in the TME^13,57,58^.

To elucidate if the activation of Nrf2 observed in TIL Tregs is the result of cues from the TME, we then conducted in vitro culture experiments in which healthy donor CD4+ Tconv and Tregs were exposed to lactic acid, a key HCC metabolite^59–65^, or tissue homogenates (derived from either HCC or background HCC liver tissues). Tregs exhibited increased expression of Nrf2 target genes (*NQO1*, *HMOX1* and *GCLM*) in response to both lactic acid and HCC homogenates, but not when exposed to background HCC tissue homogenates. In contrast, this was not observed when Tconv were cultured in the same conditions (Fig. 5h). MCT1-mediated intracellular lactate transport was required for Treg Nrf2 activation, given that the effect of lactic acid on Nrf2 target gene expression was reversed in the presence of the MCT1 inhibitor AZD3965 (Fig. 5i). Finally, we determined that the increase in Nrf2-dependent transcription resulting from lactate uptake was due to lactoylation of Keap1 (Fig. 5j), a post-translational modification that modifies Keap1 cysteine sensor residues and triggers Nrf2 translocation^66^. Additionally, using siRNA-mediated Nrf2 knockdown in human Tregs, we confirmed that Nrf2 does not regulate MCT1 expression (Supplementary Fig. 13). These findings establish a unidirectional signaling cascade in which MCT1-mediated lactate uptake drives Keap1 lactoylation, which in turn triggers Nrf2 activation.

### Nrf2 activation allows Tregs, but not Tconv, to overcome the metabolic constraints imposed by the TME

**Fig. 6:**
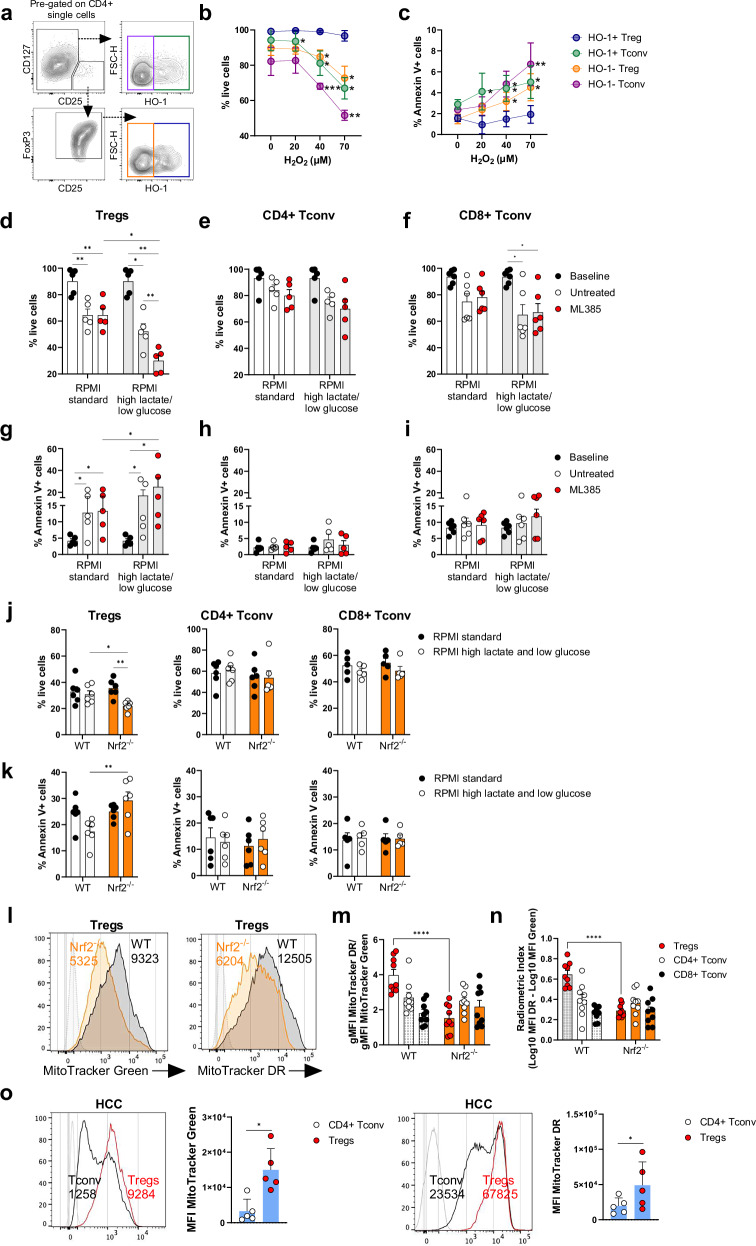
In vitro modulation of Nrf2 under TME conditions. **a** Representative gating strategy of Nrf2-activated cells (HO-1+) within Tregs (CD127^lo^CD25^hi^FoxP3+) and CD4+ Tconv (CD127+ CD25−). **b**, **c** Frequency of live and Annexin V+ cells within HO-1+ or HO-1-Tregs and CD4+ Tconv. **d**–**i** Frequency of live and Annexin V+ cells in donor Tregs (CD4+CD127^lo^CD25^hi^) and Tconv (CD4+ CD25− and CD8+) before (baseline) and after 24 h culture in RPMI standard or high lactate and low glucose with and without the NRF2 inhibitor ML385. **j**, **k** Frequency of live and Annexin V+ cells of Tregs (CD4+ CD25+) and Teff (CD4+ CD25− and CD8+) of WT and Nrf2^−/−^ mice after 24 h culture in RPMI standard or high lactate and low glucose. **l** Example histograms of the mean fluorescence intensity (MFI) of MtGreen and MtDR in freshly isolated Tregs (CD4+CD25+) of WT and Nrf2^−/−^ mice. **m** Ratio of the geometric MFI (gMFI) of MtGreen and MtDR and **n** radiometric index on freshly isolated Tregs, Tconv (CD4+ CD25− or CD8+) of WT and Nrf2^−/−^ mice. The radiometric index was calculated as the difference between the log₁₀-transformed MFI of MtDR and the log₁₀-transformed MFI of MtGreen. **o** Example histogram on Tregs and CD4+ Tconv (left, CD4+CD25+ and CD4+ CD25−) and mean MFI (right) of MtGreen and MtDR in HCC samples. Data are presented as mean values + SD or ±SD as appropriate. *P*-values were calculated by two-way ANOVA followed by Tukey’s multiple comparisons post-hoc test for (**b**–**n**); or paired t-test (two-sided) for (**o**). Only statistically significant *P*-values are displayed: *, *P* < 0.05; **, *P* < 0.01; ***, *P* < 0.001. MtDR, mitotracker deep red; MtGreen, mitotracker green. FACS sequential gating strategy for (**d**–**k**) is provided in Supplementary Fig. 26a.

Oxidative and metabolic stress are hallmarks of the TME^60^. While both Tregs and Tconv up-regulated Nrf2 in the TME, Tregs exhibited significantly higher levels of activation compared to Tconv (Supplementary Fig. 12c). To determine the functional consequences of different degrees of Nrf2 activation in the presence of environmental stressors, we conducted in vitro experiments in which cells were exposed to either H_2_O_2_ or high lactate and low glucose conditions. First, we cultured healthy donor CD4+ T cells with increasing concentrations of H_2_O_2_ in the presence/absence of the Nrf2-inducer dimethyl fumarate (DMF). DMF treatment induced the Nrf2 transcriptional target HO-1 (Fig. 6a) and preserved the viability of Tregs, but not Tconv, even when exposed to the highest concentration of H_2_O_2_ (Fig. 6b, c). Conversely, the Nrf2 inhibitor ML385^67^ (Supplementary Fig. 14) decreased the viability of Tregs when cultured in high lactate and low glucose conditions (Fig. 6d, g), but did not compromise Tconv survival (Fig. 6e, f, h, i). Similar results were observed when we compared Nrf2-deficient (Nrf2^−/−^) and wild type (WT) murine T cells (Fig. 6j, k).

Given previous data suggesting a role for Nrf2 in mitochondrial regulation and biogenesis^57,68^, we next assessed mitochondrial mass and membrane potential in WT and Nrf2^−/−^ T cells using flow cytometry. As compared to their WT counterparts, Nrf2-deficient Tregs exhibited significantly reduced mitochondrial mass (MitoTracker Green; MtGreen) and membrane potential (MitoTracker Deep Red; MtDR). In contrast, no such differences were observed when comparing WT and Nrf2^−/−^ CD4+ and CD8+ Tconv (Fig. 6l–n), further supporting the selective dependence of Tregs on Nrf2-mediated mitochondrial regulation. Similar differences between Tregs and Tconv were observed when analysing freshly isolated TILs (Fig. 6o). HCC Tregs were almost exclusively MtGreen^hi^ and MtDR^hi^ (Fig. 6o), consistent with high content of polarised mitochondria and suggestive of a high bioenergetic demand. In contrast, a large proportion of HCC CD4+ Tconv exhibited depolarised mitochondria, as depicted by the lower MFI of the MtDR and MtGreen compared to HCC Tregs (Fig. 6o), in keeping with a dysfunctional phenotype.

### Nrf2 inhibition decreases tumour burden and intra-tumoral Treg infiltration in murine models of HCC

**Fig. 7:**
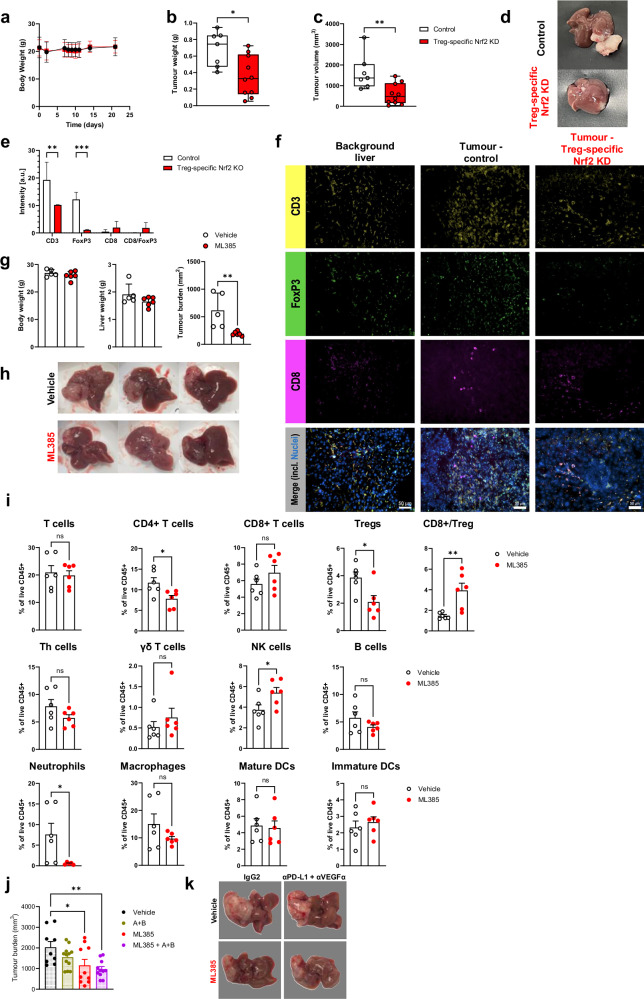
Inhibition of Nrf2 decreases tumour burden and intra-tumoral Treg infiltration in a murine model of HCC. Graphs showing **a** body weight, **b** tumour weight and **c** tumour volume of FoxP3^GFPCre^ERT2Nrf2^fl/-^(controls, *n* = 7) and FoxP3^GFPCre^ERT2Nrf2^fl/fl^ (Treg-specific Nrf2 knock-down, KD, *n* = 10) after HCC induction. Box and whiskers plots display the full range (minimum to maximum), with the center line indicating the mean. **d** Brightfield images of whole liver from a representative control and a Treg-specific Nrf2 KD mice. **e** Quantified fluorescence signal from the immunofluorescence staining of background liver and FFPE-embedded tumours of controls (*n* = 7) and Treg-specific Nrf2 KD (*n* = 10) after HCC induction. **f** Representative images captured by Zeiss Axio Observer Inverted Microscope, scale bars were 50 µm. CD3⁺ cells are shown in yellow, FoxP3⁺ in green, CD8⁺ in red, and nuclei in blue. Graphs showing **g** body weight, total liver weight and tumour burden in orthotopic HCC mice treated with vehicle or ML385. **h** Representative pictures of liver orthotopic model tumours in vehicle or ML385-treated mice. **i** Frequency of immune cell populations among live CD45+ cells in the tumours of mice treated with vehicle or ML385. FACS sequential gating strategy is provided in Supplementary Fig. 26b. **j** Tumour burden and **k** representative pictures of liver in MASH-HCC mice treated with vehicle, atezo+bev (αPD-L1 + αVEGFα) (A + B), ML385 or ML385 and atezo+bev. In all figures, *n* (or each dot in box plots) represents an independent biological replicate. Data are presented as mean values + SD as appropriate. *P*-values were calculated by unpaired *t*-test (two-sided) for (**b**, **c**, **e**, **g**, **i**); or 2-way ANOVA followed by Šídák’s multiple comparisons post-hoc test versus vehicle (control group) for (**j**). *, *P* < 0.05; **, *P* < 0.01; ns, not significant.

To confirm the relevance of Nrf2 expression in intra-tumoral Tregs in vivo, we generated a tamoxifen-inducible Treg-specific Nrf2-deficient C57BL/6 murine strain (FoxP3^GFPCre^ERT2Nrf2^fl/fl^) by crossing Nrf2^fl/fl^ with Foxp3GFP-cre mice (Supplementary Fig. 15), and employed a well-established orthotopic HCC model in which Hep-53.4 cells are implanted into the left hepatic lobe^69^. Hep-53.4 are high mutational burden cells and lead to hot tumours that respond to anti-PD-1 blockade therapy^69^. Under steady-state conditions, the knock-down of Nrf2 in Tregs following tamoxifen treatment had no noticeable immunological effects (mice maintained normal weight and CD4+/CD8+ T cell numbers in secondary lymphoid organs; Fig. 7a and Supplementary Fig. 16). In contrast, the Treg-specific deletion of Nrf2, 7 days after Hep-53.4 implantation, significantly reduced tumour burden (Fig. 7b–d). This was associated with decreased accumulation of Tregs within the tumours, and a trend towards an increased CD8+/Treg ratio, with no changes in Treg numbers in the non-tumoral background liver and the spleen (Fig. 7e, f and Supplementary Fig. 16). We next investigated whether similar results would be observed following the systemic inhibition of Nrf2 using ML385. Tumour burden in ML385 treated mice was substantially lower than in controls (Fig. 7g, h). While ML385 has been shown to directly inhibit cancer cell growth, we observed a drastic shift in the intra-tumoral balance between immunosuppressive (Tregs and neutrophils) and effector (CD8+ T and NK cells) immune cell subsets (Fig. 7i). Again, no obvious immunological changes in the non-tumoral liver were detected (Supplementary Fig. 17). These results indicate that inhibiting Nrf2, either in Tregs or systemically, has a strong and selective impact on the immune TME.

We conducted additional experiments using ML385 in a pre-clinical model of human metabolic dysfunction-associated steatotic liver disease (MASLD) in which C57BL/6 mice were fed a modified diet of high sugar and fat (Western diet) for 3 months prior to tumour implantation. This induces liver steatosis (Supplementary Fig. 18) and results in larger tumours that are refractory to PD-1 blockade^69^. As previously reported^69^, anti-PDL1/anti-VEGF blockade (atezolizumab/bevacizumab) did not influence tumour growth in this model. In contrast, ML385 significantly reduced tumour burden compared to vehicle-treated controls (Fig. 7j, k). Combining Nrf2 inhibition with anti-PDL1/anti-VEGF blockade provided stronger anti-tumoral effects (although no statistically significant differences were observed when compared with ML385 alone). Taken together, these results suggest that Nrf2 inhibition could constitute a therapeutic strategy for clinical HCC, particularly for patients with inflamed tumours and those with underlying MASLD^54^.

## Discussion

In contrast to effector T cells, Tregs exhibit a remarkable capacity to adapt their metabolic programmes to the constraints imposed by the TME, which allows them to survive, expand, and exert powerful immunosuppressive effects. Treg metabolic adaptation involves the upregulation of key transporters such as CD36^13^ and MCT1^58^, which facilitate the utilization of lipids and lactate, respectively, contribute to mitochondrial function, and are key to promote Treg fitness within the nutrient-depleted TME. However, the overarching tissue context-dependent mechanisms orchestrating these changes remain to be understood, particularly in humans. This limits the development of therapeutic attempts to selectively neutralize intra-tumoral Tregs without compromising local effector T cell function and/or systemic self-tolerance networks.

Our study delineates the heterogeneity and homeostatic properties of human Tregs residing within the non-tumoral liver and in HCC, and uncovers a pivotal role for Nrf2 activation, via lactate, in regulating Treg metabolic adaptation to the TME. We report here that circulating Tregs trafficking to the non-tumoral liver undergo two stages of adaptation. In response to the local liver microenvironment they strongly activate TNF-α/NFκB signalling but, in contrast to what has been observed in other tissues^14,16^, the majority become metabolically inert and prone to apoptosis. These findings suggest that under steady-state conditions Tregs are not an essential component of the liver immune microenvironment. Of note, the small subset of Tregs that receive TCR stimulation exhibit a different fate by turning on metabolic pathways known to promote their survival and function (e.g. TCA cycle, fatty acid and oxidative metabolism), which allows them to preferentially expand. The homeostatic features of intra-hepatic Tregs in the non-tumoral liver are drastically modified in HCC, in which Tregs accumulate in large numbers and exhibit a much greater degree of tissue adaptation. This is illustrated by their mature phenotype, low apoptotic rate, increased suppressive function, high TCR oligoclonality, and active bioenergetic metabolism. While the capacity of HCC TIL Tregs to simultaneously engage glycolytic, oxidative phosphorylation, and fatty acid metabolism has been previously reported^58,70^, we now show that their increased survival and functional fitness are critically dependent on Nrf2 signalling. Nrf2 activation, mediated by lactate uptake via MCT1, is coordinated with the expression of transporters for diverse nutrient sources, including lipids (CD36, FABP5, CPT1A), lactate (MCT1), and amino acids (CD98, ASS1). Our results indicating a key role for Nrf2 in regulating Treg intracellular metabolism are supported by previous studies in macrophages and cancer cells, demonstrating that Nrf2 controls metabolic pathways promoting cell proliferation^71^, maintains mitochondrial function^72^, and controls the expression of CD36^73–76^. We propose that the lactate-driven mechanism described here provides a proportionate, stress-responsive, Nrf2 activation that maintains Treg viability under hostile metabolic conditions, and that is fundamentally different from what is observed when Nrf2 signalling is constitutively or pharmacologically perturbed in non-cancer models^77,78^.

Importantly, we provide evidence that although all lymphocyte subsets activate Nrf2 in response to the TME, the downstream functional consequences of Nrf2 activation are drastically different for Tregs and Tconv. The differential effect of Nrf2 inhibition on intra-tumoral Tregs and Tconv explains the results of our in vivo murine model, in which inhibiting Nrf2 resulted in drastically reduced tumour progression even when the inhibitor was delivered systemically. Indeed, Nrf2 activation was recently shown to accelerate the terminal exhaustion of intra-tumoral CD8+ T cells^79^. Taken together, these findings indicate that Nrf2 contributes to the immunosuppressive nature of the TME both by enhancing Treg survival and by promoting effector T cell exhaustion.

The immunotherapeutic approaches currently available in HCC have limited efficacy. Our results revealing that TIL Tregs are the main regulatory lymphocyte subset in HCC and that their metabolic programming is crucially dependent on Nrf2 have, therefore, important clinical implications. This is clearly illustrated by our finding of a significant association between enrichment in Nrf2-activated Tregs and reduced PFS following first-line HCC treatment. Given the enhanced anti-HCC activity observed in MASLD mice when combining Nrf2 targeting with immune checkpoint inhibition, we anticipate that this approach may be particularly valuable for overcoming treatment resistance in this growing patient population. However, given the capacity of Nrf2 activation to ameliorate MASLD, clinical translation would require direct administration of Nrf2 inhibitors into HCC tumours or development of compounds capable of selectively inhibiting Nrf2 in the TME.

Our study has several limitations. Due to the limited yield of intra-hepatic Tregs obtained from clinical specimens, we could not sort the Tregs subpopulations identified by scRNA-seq to conduct functional assays, nor elucidate the specific mechanisms responsible for the increased apoptosis of intra-hepatic Tregs. Furthermore, our findings were restricted to HCC; whether they can be extrapolated to other cancers will require further research. This is exemplified by a previous study in ovarian cancer^80^ that employed a different experimental design to ours (i.e. comparison of TIL Tregs with TIL Tconv rather than analysis of Tregs across different tissue compartments), and reached divergent conclusions as to the role of Nrf2 in intra-tumoral Treg fitness.

In short, Nrf2 plays a key role in orchestrating the metabolic adaptation of Tregs to the HCC TME. We propose that targeting Nrf2, especially in HCC tumours exhibiting inflamed and immune-exhausted features, may provide a strategy to selectively disrupt intra-tumoral Treg function while preserving, and potentially enhancing, anti-tumour immunity.

## Methods

### Study design

The overarching aim of this study was to explore the molecular and metabolic programs of liver-resident Tregs in different compartments and understand the mechanisms that define their survival and fitness in HCC. We hypothesized the HCC microenvironment would promote Treg accumulation by activating Nrf2, thereby ensuring their metabolic fitness and survival. Our objectives were: (1) to identify the key determinants of Treg liver tissue residency; (2) to investigate how these processes are impacted by HCC development; and (3) to elucidate the contribution of Nrf2 signalling to the homeostasis of intra-tumoral Tregs and to the progression of HCC.

We conducted a cross-sectional study that included phenotypic, transcriptomic, metabolic, and functional analyses of human HCC Tregs and those isolated from paired background non-tumor and healthy liver tissues. We first used scRNA-seq to explore the molecular determinants of intra-hepatic Treg tissue residency by comparing healthy liver tissue and paired PBMC samples. Next, we re-analyzed the scRNA-seq data from healthy liver tissue Tregs together with data derived from HCC and paired background HCC tissue specimens to elucidate the key features of HCC TIL Tregs. The preferential activation of Nrf2 by HCC TIL Tregs was confirmed employing CyTOF analyses and in vitroassays. The effects of Treg Nrf2 activation in vivo were then investigated using two HCC murine models. Finally, we confirmed the generalizability and clinical relevance of our findings employing gene expression, histology, and survival data from large cohorts of patients with HCC.

All flow and mass cytometry, scRNA-seq, in vitro and in vivo studies were conducted using biological replicates (*n* numbers described in the main manuscript or figure legends). qPCR assays were carried out with two technical replicates, while human Treg microsuppression assays were performed with a minimum of three technical replicates. To prevent any technical bias, each CyTOF staining batch included an internal reference consisting in PBMCs from the same healthy donor.

For studies involving HCC clinical specimens no prior sample size estimation was performed. For studies involving mice, sample sizes were based on previous studies in the laboratory. We did not exclude data from the analysis.

### Human Samples

Human liver and blood samples were obtained from patients undergoing liver resection surgeries or liver transplant. Informed written consent was obtained from all donors (REC approval number: 15/NS/0062). All samples were obtained under the remit of the King’s College Hospital Liver Tissue Bank (UK Research Ethics Committee approval ref. 23/LO/0708; IRAS ID: 332608). Demographic and clinical characteristics of study participants are included in Supplementary Data 8.

### Mice

All procedures were conducted in accordance with the UK Home Office Animals (Scientific Procedures) Act 1986 and ARRIVE guidelines and approved by King’s College London’s AWERB. All procedures were performed in accordance with all legal, ethical, and institutional requirements (PP4067431 and PP8854860) as dictated by UK legislation and ethical review committees at King’s College London and Newcastle University.

All mice were housed under standard specific pathogen–free conditions, with a 12 h light/12 h dark cycle, an ambient temperature of 20–24 °C, and relative humidity maintained between 45 and 65%. Food and water were provided ad libitum. Experimental and control mice were co-housed.

#### Wild type and global Nrf2 knockout mice

C57BL/6JOlaHsd wild-type mice (6–9 weeks old) were obtained from Envigo and NRF2 knock-out (KO) mice (6–9 weeks old) were provided by Dr. Sarah Chapple and Prof. Giovanni E. Mann (King’s College London), with the original source of mice obtained from Prof Masayuki Yamamoto, Division of Medical Biochemistry, Tohoku University Graduate School of Medicine, Tohoku University, Japan. Both mice were housed together in a specific pathogen-free condition in accordance with institutional guidelines and ethical regulations. Equal numbers of female and male mice were used, and the number of animals included in each experiment is indicated in the corresponding figure.

#### Murine tumour model in a tamoxifen-inducible Treg-specific Nrf2-knock-down (FoxP3^GFPCre^ERT2Nrf2^fl/fl^)

We generated the tamoxifen-inducible Treg-specific Nrf2-knock-down mice model (FoxP3^GFPCre^ERT2Nrf2^fl/fl^; Supplementary Fig. 15). The FoxP3CreERT2^+/+^Nrf2^−/−^ mice (gift provided by Dr. Ali/King’s College London) were crossed with FoxP3CreERT2^−/−^Nrf2^fl/fl^ mouse (gift provided by Prof. Shah/King’s College London) to obtain the FoxP3CreERT2^+/-^Nrf2^fl/−^ intermediate offsprings that were heterozygous both in the FoxP3CreERT2 and Nrf2 alleles. The FoxP3CreERT2^+/−^Nrf2^fl/−^ mice were subsequently cross-bred to select control (FoxP3CreERT2^+/+^Nrf2^fl/−^) and Treg-Nrf2 KD (FoxP3CreERT2^+/+^Nrf2^fl/fl^) animals, respectively.

Female adult mice (10–15 weeks old, 20.8 ± 3.6 g) were used for the animal experiments (Control: FoxP3-Cre^+/+^ Nrf2^fl/−^; Treg-Nrf2 KD: FoxP3-Cre^+/+^ Nrf2^fl/fl^). The number of animals included in each experiment is indicated in the corresponding figure.

All mice were maintained within the King’s College London Biological Services Unit under specific pathogen-free conditions in a dedicated and licensed air-conditioned animal room. Female mice were used to establish the orthotopic liver tumour models with mycoplasma-free Hep53.4 cells. After a week of acclimatisation, mice with correct genotype (Supplementary Figs. 19, 20 and Supplementary Table 6) were randomly allocated into two cohorts with five individuals each, anaesthetised with 2% (v/v) isoflurane/O_2_, shaved on their abdomen, subcutaneously given 0.3 mg/kg of buprenorphine, and after 30 min, each mouse received an injection of 1 × 10^6^ Hep53.4 cells suspended in 20 μL of 30% Matrigel/PBS solution into the left lobe of the liver. Following 7-day of tumour cell implantation, each mouse was intraperitoneally injected with tamoxifen at 100 µg/g prepared in corn oil for five consecutive days. At day 21 after tumour inoculation, mice were euthanized by cardiac punction exsanguination followed by cervical dislocation. The spleen, liver and tumour tissues were harvested for downstream analysis. Tumours were weighed and measured by callipers to evaluate the tumour burden using the formula: 0.5 × *L* × W2, wherein *L* represents tumour length and *W* its width.

#### Lean orthotopic HCC murine model

For the orthotopic HCC murine model, intrahepatic injection of 1 x 10^6^ Hep-53.4 cells into the left lobe of normal chow diet-fed C57BL/6 male mice was performed under isoflurane general anaesthesia. ML385 therapeutic intervention was initiated 14 days post-implantation, at which point small macroscopic tumors were established. The treatment regimen consisted of daily intraperitoneal injections (IP) of either ML385 (30 mg/kg dissolved in a vehicle solution containing 10% DMSO, 40% PEG300, and 5% Tween 80) or vehicle control. Mice were euthanized at 28 days post-implantation, and tissues were harvested for subsequent analyses. Tumor burden was quantified using digital calipers, with measurements taken in three dimensions to calculate tumor volume (mm³).

#### Orthotopic MASH-HCC murine model

For the MASH-HCC murine model, C57BL/6 male mice were fed ad libitum a modified western diet (Envigo -TD.120528) plus sugar water (23.1 g/L fructose and 18.9 g/L glucose) for 3 months prior to Hep53.4 cells implantation in the liver. Tumours were left growing for 3 weeks before applying the treatments, consisting of either daily IP injection of ML385 (30 mg/kg), twice a week IP injection of anti-PD-L1 (150 μg per mouse, BE0101, BioXCell, atezolizumab) plus anti-VEGFα (150 μg per mouse, BE0399, BioXCell, bevacizumab), or a combination of both, for the last 14 days prior to harvest. MAFLD diagnosis was confirmed through histopathological assessment using the MASH scoring system, which evaluates key features including steatosis, lobular inflammation, and hepatocellular ballooning (Supplementary Fig. 18 and Supplementary Table 7).

Mice were monitored daily and were humanely euthanised if predefined welfare criteria were reached, including tumour burden limits (≥15 mm by imaging or ≥1 cm palpable mass), excessive body weight change (>25% loss or >20% gain associated with abdominal distension), or evidence of metastatic disease. Animals were also culled if they exhibited persistent clinical signs of distress (e.g., hunched posture, abnormal locomotion, ruffled fur, diarrhoea, anaemia, or inability to eat or drink) that did not improve with supportive care within 24 h, or if surgical complications occurred following intrahepatic injection.

### Cell isolation and separation

Peripheral blood mononuclear cells (PBMCs) were isolated from heparinised blood by density centrifugation using Ficoll-Paque PLUS (Sigma-Aldrich). PBMCs were used fresh or were frozen in CryoStor® cell preservation media (Sigma-Aldrich) and stored until the moment of analysis.

Cells from human spleen and lymph nodes obtained from healthy liver donors were isolated by mechanic tissue disruption, and red blood cells were lysed with RBC lysis buffer (Biolegend).

Tissue samples obtained from different surgical liver resections (HCC and paired non-tumoral background tissues from HCC resections; and histologically healthy liver tissue from colorectal liver metastasis resections) were collected in cold DMEM medium (Life Technologies) and processed within 2 h. Tumour and tissue single cell suspensions were obtained by enzymatic digestion with the BD Horizon^TM^ Dri Tumour & Tissue Dissociation Reagent (TTDR) (BD Biosciences) as per manufacturer instructions. Briefly, tissue was minced in small pieces using scalpels, extensively washed, and then incubated with 1XTTDR in a rocker for 30 min at 37 °C and 5% CO_2_. After full digestion, the dissociation reaction was filtered through a 70 µM strainer and centrifuged at 250 × *g* for 8 min. Intrahepatic or intra-tumoral lymphocytes were isolated as described in ref. ^81^. First, dead hepatocytes were removed by centrifugation at 800 × *g* for 15 min on a 30% Percoll gradient (GE Healthcare). Leukocytes were then isolated by density centrifugation using Ficoll-Paque PLUS and used fresh or stored in CryoStor® cell preservation media (Sigma-Aldrich).

Human CD8+ and CD4+ T cells were isolated from buffy coats using the Rosette-Sep Human CD8+ T cell and CD4+ T cell enrichment cocktails, respectively (Stem Cell Technologies). CD4+CD25+ (Tregs) were enriched by magnetic separation using the Human CD25 Microbeads II and LS columns (Miltenyi Biotec), according to manufacturer’s instructions. CD4+CD25- (Tconv) were recovered as the negative fraction from the CD25+ cell enrichment.

Murine cells were isolated from the spleen and lymph nodes: CD8+ Tconv were enriched using the EasySep^TM^ Mouse CD8a Positive Selection Kit II (Stem Cell Technologies), CD4+CD25- (Tconv) and CD4+CD25+ (Tregs) were enriched using the EasySep^TM^ Mouse CD4+CD25+ Regulatory T Cell Isolation Kit II (Stem Cell Technologies) as per manufacturer’s protocols.

### Flow cytometry

All flow cytometry samples were acquired on a BD LSR Fortessa flow cytometer (BD Biosciences) and analysed using the Flowjo software (v10.7.1). A complete list of all commercially available fluorochrome-conjugated anti-human and anti-mouse monoclonal antibodies used in the study is summarised in Supplementary Table 8.

Cells were first labelled with Live/Dead^TM^ Fixable Dead Cell Aqua or Violet Stain kits (Thermo Fisher) in Phosphate-Buffered Saline (PBS, Gibco) for 20 min at room temperature or 10 min at 4 °C, respectively. Extracellular marker staining was performed in staining buffer (PBS + 0.9% Bovine Serum Albumin, BSA, Sigma-Aldrich) for 30 min at 4 °C. For intracellular antigen labelling, the cells were fixed and permeabilized using the eBioscience Fixation/Permeabilization reagents (Thermo Fisher) according to manufacturer’s instructions. Human Tregs were identified following the gating strategy depicted in Supplementary Fig. 1. Within the CD4+ gate, three Treg subsets were distinguished based on CD45RA and FoxP3 expression as described by ref. ^82^: (1) CD45RA⁺ FoxP3^lo^, naïve/resting Tregs; (2) CD45RA⁻ FoxP3^lo^, cytokine-secreting Tregs; (3) CD45RA⁻ FoxP3^hi^, activated/effector Tregs.

To detect apoptosis, cells were stained with Annexin V-FITC in Annexin V binding buffer (all from Biolegend) for 10 min at room temperature.

MitoTrackers’ staining was performed as described in ref. ^83^ with slight modifications. 1 × 10^6^ freshly isolated cells were resuspended in pre-warmed RPMI 1640 medium and stained with MitoTracker Deep Red FM and MitoTracker Green FM (Thermo Fisher) at a final concentration of 12.5 nM and 20 nM, respectively, for 20 min at 37 °C in a humidified atmosphere with 5% CO_2_. Cells were then washed in PBS and stained with LIVE/DEAD^TM^ Fixable Violet dye (Thermo Fisher) for 10 min at 4 °C. Cells were washed again in cell staining buffer and incubated with surface antibodies for 30 min at 4 °C. Cells were left unfixed for flow cytometric analysis.

### Single-cell RNA sequencing

#### Isolation of human CD4+ T cells

Cryopreserved samples were thawed in RPMI 1640 medium (Thermo Fisher) supplemented with 10% foetal bovine serum (FBS, Gibco), 1% penicillin/streptomycin (P/S, Gibco) and 1% L-Glutamine (Sigma-Aldrich) (RPMI 1640 complete medium), and washed twice with PBS. CD4+ cells were enriched by using the human CD4+ T Cell Isolation Kit (Miltenyi Biotec) prior to CITE-seq staining and cell sorting.

#### CITE-seq staining and selection of CD4+CD25+ (Tregs) and CD4+CD25- (Tconv) cells in blood and healthy liver

Cells were first incubated with Human TruStain Fc Blocking Reagent (Biolegend) for 10 min at 4 °C. After incubation, biotin CD25 antibody was immediately added for 20 min at 4 °C. To ensure unbound biotin antibody was removed, cells were washed three times with Cell Staining Buffer (Biolegend) and then incubated with Total Seq-C APC SAV antibody for 20 min at 4 °C. Cells were washed twice with Cell Staining Buffer. In the last staining step, cells were incubated with an antibody cocktail containing flow, CITE-seq and cell hashing antibodies for 20 min at 4 °C. Cells were washed twice with Cell Staining Buffer and resuspended in 2% BSA in PBS. For the detection of dead cells, DAPI (Biolegend) was added before cell sorting. Finally, cells were sorted on a BD FACSAria™ Cell Sorter by gating on two populations: a CD25+ population to enrich for CD4+ regulatory T cells and a CD25- fraction (Supplementary Fig. 21, 22a). All flow, CITE-seq and cell hashing antibodies are detailed in Supplementary Table 9.

#### CITE-seq staining and selection of CD4+CD127^lo^CD25^hi^ (Tregs) and CD4+CD25- (Tconv) cells in tumour and paired background tissues

Cells were first incubated with Human TruStain Fc Blocking Reagent (Biolegend) for 10 min at 4 °C. After incubation, an antibody cocktail containing flow, CITE-seq and cell hashing antibodies was added for 30 min at 4 °C. Cells were washed twice with Cell Staining Buffer and resuspended in 2% BSA in PBS. For the detection of dead cells, DAPI (Biolegend) was added before cell sorting. Finally, cells were sorted on a BD FACSAria™ Cell Sorter by gating on two populations: a CD127^lo^ CD25^hi^ population to identify CD4+ regulatory T cells and a CD25- fraction (Supplementary Figs. 21 and 22b). All flow, CITE-seq and cell hashing antibodies are detailed in Supplementary Table 9.

#### Single cell immune profiling

Cell count and viability were assessed before loading the samples in the Chromium Controller (10x Genomics). Treg and Tconv cells from the 2 different compartments, either healthy liver and blood or tumour and background tissues, of the same patient were multiplex in a single run. scRNAseq libraries (5´ gene expression library GEX, T cell receptor enriched library VDJ, and surface protein library containing the TotalSeq-C CITE-seq antibodies) were prepared using the Chromium Single Cell Immune Profiling kit (10x Genomics, v1.1) according to the manufacturer’s instructions. The generated scRNAseq libraries were sequenced using a total of 100 cycles (paired-end reads) with a NovaSeq 6000 S2 flow cell (Illumina).

#### Data processing and quality control

Sequence reads from all samples were processed and aggregated using Cell Ranger (10X Genomics, v7.2). To clean the data, we filtered out cells that had (i) less than 500 and more than 15,000 counts; (ii) less than 500 and more than 2000 genes expressed; and (iii) mitochondrial counts above 10%. In addition, genes detected in less than 10 cells were removed, and only 10,000 most variable genes were kept. Proteins were normalised in log2, and RNA was library-size corrected using the “*normalize_total*“ function and normalised to natural logarithm by using the function “*log1p*” function from the “*scanpy*” package^84^. To account for batch effect among different samples in each data set, we used BBKNN integration method^85^, available in “*scanpy*” with the “*bbknn*” function^84^.

#### Dimensionality reduction methods and cell clustering

The "*scanpy”* package (v1.9.6) implemented in Python (v3.9.13) was used to perform dimensional reduction methods. To obtain an overview of the datasets showing the relationship between cell population clusters, UMAP was used with the default settings. To find clusters in the data, we used the Leiden algorithm with different resolutions parameters. To identify unique differentially expressed genes (DEGs) among each cluster, the “*rank_genes_groups*” function from “*scanpy*” was used, and non-parametric Wilcoxon rank sum tests were set to evaluate the significance of each individual differential expressed gene (DEG).

#### Tissue digestion effect

To remove the confounding effect of the liver tissue dissociation protocol when comparing the transcriptional profile of blood and intra-hepatic Tregs, we first compiled from the literature a list of the top 138 genes whose expression is significantly increased across various cell populations following tissue dissociation at 37 °C^14,86–88^ (Supplementary Data 9).

CD4+ cells from the healthy liver underwent UMAP analysis (Supplementary Fig. 23), revealing the identification of predominantly affected cells within a unique cluster (cluster 1). The transcriptional profile of cluster 1 showed a high overlap with the dissociation-induced gene expression (Supplementary Table 10 and Supplementary Data 9). Moreover, examination of the top 30 upregulated genes in cluster 1 revealed high levels of immediate early genes and heat-shock proteins. Despite the presence of cells expressing these genes in other clusters, they were grouped with cells showing no discernible digestion effect on their transcriptomes. This grouping suggests that the transcriptional profile of these cells is minimally affected, justifying their retention in subsequent analyses. Subsequently, cluster 1, comprising 3223 cells (19% of the total), was excluded from further analysis, demonstrating a more stringent criterion compared to prior investigations^14,35,89^.

#### Dataset integration

To mitigate the batch effect between patients, we performed an integration step for each data set by applying the BBKNN method^85^ available in “scanpy” with the “bbknn” function. No additional integration step was needed when subsetting Treg and Tconv populations, as no clear batch effects were visible.

For the analysis of all grouped compartments, cells from blood had to be discarded as this compartment hindered the integration process (Supplementary Fig. 24), making the subsequent analyses complicated as the various clusters identified would be compartment specific. When keeping cells from all compartments, no integration approach managed to mitigate batch effects between samples and tissues efficiently without losing too much of the cell heterogeneity (Supplementary Fig. 24, middle). This effect if most probably due to the preparation protocols, which are quite different for blood samples. Thus, we opted to remove the blood compartment and repeated the integration process above with more success, with both the patient and compartment batch effects being mitigated (Supplementary Fig. 24, bottom).

#### Cluster cell type identification

Cluster cell types were annotated by using selected canonical markers for CD4+ T cell subsets from publicly available data sets: Tregs, Tconv, naïve, CM, EM and TEMRA (Supplementary Data 1 and 2). Pre-ranked GSEA was used to delineate the cell type for each cluster based on the enrichment score (Supplementary Tables 1–4). These analysis were performed by using the GSEA software (v4.3.2) from UC San Diego and Broad Institute^90^. DEGs, obtained by comparing one cluster vs the rest, ranked by fold-change, were used as input.

Clusters that did not share the core CD4+ T cell transcripts and lacked CD4 protein expression were deemed as contamination and excluded from further analysis: cluster 17 from the analysis of blood and paired healthy tissue, and cluster 12 from the analysis of healthy liver tissue, background non-tumoral and paired HCC tissues.

Treg clusters’ identity was further justified by plotting the expression of key Treg genes (*FOXP3*, *IL2RA*, *IKZF2* and *CTLA4*) in the UMAP (Supplementary Fig. 25).

#### Pathway analysis: GSEA and GSVA

GSVA was performed through the *‘GSVA’* package (https://github.com/jason-weirather/GSVA) (v1.0.6)^91^. GSEA analyses were performed using the "*GSEApy”* package (v1.1.0) with removing pathways that contained less than 5 or more than 1000 genes^92^. For the pre-ranked analysis, the list of genes was first generated by ranking the DEGs, obtained by comparing one cluster *vs* the rest, by fold-change and then using in the "*prerank”* function. For a standard GSEA analysis, differentially genes obtained with the "*rank_genes_groups”* function in *‘scanpy’* were used as an input^84^.

#### TCR repertoire analysis

TCR repertoire analysis was conducted using the “*scirpy*“ package (v0.13.1) in Python^93^. Cells falling into the categories of orphan chains (those with either a single *α* or *β* receptor chain), extra chains (cells possessing a complete *α*/*β* receptor pair along with an additional chain), and multi-chains (cells with more than two detected receptor pairs) were excluded from subsequent analyses.

#### Pseudotime trajectory analysis

Pseudotime and bifurcation analysis were conducted using “scFates v1.1.1” in Python. This package is part of the “*scverse*” ecosystem, which makes it fully compatible with the other Python packages used in our analysis pipeline.

### Transcriptomic analysis of publicly available scRNA-seq and bulk RNA-seq HCC datasets

To generate the most clinically relevant signature to define cluster B’ we first computed the list of genes differentially expressed between cluster B’ Tregs and all other CD4+ T cells. Next, we use the differentially expressed genes to (a) characterize cluster B’ Tregs and their association with molecular and immune features of HCC TME (64-gene signature of upregulated genes), and (b) characterize Cluster B’ Tregs for clinical outcome purposes, with a 378- gene signature (180 up and 199 down).

Therefore, first for molecular and immune characterization, among the 179 genes with FDR < 0.01 and log2FC > 2, we selected 64 that were significantly associated with reduced overall survival in the TCGA database (*n* = 365) using Cox regression (LogRank *p*-value < 0.05; Hazard Ratio >1) (Supplementary Table 5). The specificity of the 64-gene signature for cluster B’ Tregs was then confirmed using an independent 170 scRNASeq HCC dataset (GSE151530). To this end, we analysed the raw expression matrices using Seurat 488 and annotated the resulting clusters into cancer cells, immune, and stromal cell types based on the expression of known marker genes89. Cluster B’ gene scores based on the above mentioned 64-gene signature were then computed per cell using the AddModuleScore function, Seurat R package. The computed gene scores were plotted within a UMAP, visualized in boxplots to assess the specificity of the gene signature among HCC cell types, and subsequently compared to our previously annotated fractions of Tregs. Once the specificity of the 64-gene signature was confirmed, it was used to evaluate presence of Cluster B’ Tregs within a cohort of 171 surgically resected fresh-frozen HCCs. Specifically, the ssGSEA module from GenePattern was used to calculate enrichment scores per sample for the Cluster B’ gene signature. Scores were then correlated with signatures derived from specific molecular and immune HCC subtypes^47,49,53,94–96^.

Second, to evaluate the impact of cluster B’ Tregs in the outcome of HCC patients treated with atezolizumab +bevacizumab from the phase Ib (NCT: GO30140) and the phase III IMbrave150 clinical trial we selected the 378 genes, 180 up-regulated genes (FDR < 0.01 and log2FC > 2) and the 199 downregulated genes (FDR < 0.01 and log2FC > 2) of genes differentially expressed between cluster B’ Tregs and all other CD4+ T cells.

#### Analysis of RNA sequencing data of atezo+bev-treated HCCs using a scRNAseq-derived Treg gene signature

RNA-seq data from 247 advanced HCC patients treated with atezolizumab +bevacizumab from the phase Ib (NCT: GO30140) and the phase III IMbrave150 clinical trials^54^ were used to assess the presence of Tregs. The positivity for the Treg signature was evaluated as reported in ref. ^55^. In brief, the Nearest Template Prediction (NTP) module from GenePattern^56^, using fold change values as weight) was used to categorize each sample according to the presence of the Cluster B’ Treg cell population. Significant prediction was defined using Benjamini-Hochberg-adjusted FDR < 0.05. NTP-predicted classes were then correlated with clinical outcome data, including progression-free survival (PFS) and response to atezo+bev based on RECIST criteria.

### Mass cytometry (CyTOF)

#### Heavy-metal conjugation of antibodies

Antibodies were conjugated to heavy-metal ions with commercially available MaxPar reagents (Fluidigm) following manufacturer instructions.

#### CD45 barcoding and staining with heavy-metal conjugated antibodies

All antibodies used can be found in Supplementary Table 11. Cryopreserved samples were thawed in RPMI 1640 complete medium (as described before). Cells (4 × 10^6^) were washed twice with cell staining medium (CSM: 1X PBS Ca^2+^/ Mg^2+^ free, Invitrogen, + 0.5% BSA + 0.02% sodium azide, Sigma-Aldrich, + 2 mM EDTA, Gibco) and immediately stained for viability with 1x Rh103-intercalator (Fluidigm) and Human TruStain Fc block reagent (Biolegend) for 15 min at room temperature. Cells were washed twice with CSM and incubated for 30 min at 4 °C with the relevant CD45 barcode and cell-surface antibody master-mix. Cells were washed twice with CSM and permeabilized with the FoxP3 Fixation/Permeabilization solution (eBioscience) following manufacturer instructions. Intracellular antibody master mix in 1X Permeabilization buffer was added to the samples and incubated for 45 min at room temperature. Cells were washed once with 1X Permeabilization buffer and resuspended in CSM. To eliminate technical variability during acquisition, individual samples within one specific patient were combined into a composite sample before further processing. After one wash with CSM, cells were resuspended in intercalation solution (1.6% methanol-free paraformaldehyde, Invitrogen, in PBS and 0.5 µM rhodium-intercalator, Fluidigm) and incubated overnight at 4 °C. Before acquisition, samples were washed once in CSM and twice in cell acquisition solution (CAS, Thermo Fisher) with 2 mM EDTA solution. Cells were then resuspended at 5 × 10^5^ cells/ml in CAS with 1:10 EQ™ four element calibration beads (Fluidigm) and acquired on a Helios mass cytometer (Fluidigm).

#### Data processing

Flow cytometry standard (FCS) files were normalized with EQ beads. Samples acquired in multiple recordings were concatenated using the CyTOF software (v7.0.8493.0). Normalized FCS files were exported and cleaned up by manually gating in Cytobank as described in ref. ^97^. Barcoded cells were assigned back to their initial samples using their unique CD45 barcode combination. Data was asinh transformed (cofactor 5) and normalized to the 99.9^th^ percentile of each respective channel. Unsupervised analysis of single cell data was conducting in Cytobank (v10.3) by using self-organising map (FlowSOM) projected onto uniform manifold approximation and projection (UMAP).

### In vitro Treg micro-suppression assay

Treg suppression ability was assessed as described in ref. ^98^. Shortly, cryopreserved samples were thawed and stained with a cocktail of monoclonal antibodies (Supplementary Table 12) for cell sorting. Suppression assays were established in V-bottom 96-well plates by sorting 500 CD4+CD25^−/lo^CD127+ effector T cells (Teffs) in the presence or absence of CD4^+^CD25^high^CD127^low^ Tregs at various ratios (Treg:Teff 0:1, 1:4, 1:2 and 1:1) with 1 × 10^3^ CD19+ B cells, using the BD FACSAria III flow cytometer automated cell deposition unit (BD Biosciences). Cells were sorted into 150 μl X-VIVO 15 (Lonza) supplemented with Penicillin-Streptomycin-Fungizone (Gibco) and 10% heat-inactivated, filtered human AB sera (Sigma-Aldrich), stimulated with PHA (4 μg/ml; Alere) and incubated at 37 °C, 5% CO_2_, for 5 days. Proliferation was assessed by the addition of 0.5 μCi/well [3H] thymidine (PerkinElmer) for the final 20 h of co-culture. Conditions were run in at least 3 replicates, and proliferation readings (counts per minute [CPM]) averaged. The percentage suppression in each culture was calculated using the following formula: percent suppression = 100 − [(CPM in the presence of Tregs ÷ CPM in the absence of Tregs) × 100]. All samples from a participant were analysed concurrently.

### In vitro modelling of tumour microenvironment conditions

To replicate the conditions of the tumour microenvironment, we sought to emulate oxidative and metabolic stress in vitro. To induce oxidative stress, healthy human CD4+ T cells were cultured in CTS^TM^ AIM-V^TM^ medium, without phenol red (Thermo Fisher), in the presence of H_2_O_2_ (Sigma-Aldrich) at 3 different concentrations (20, 40 and 70 µM) and low-dose recombinant human IL-2 (rhIL-2, 50 IU/ml, Gibco) for 18 h.

To induce metabolic stress, healthy human CD8+, CD4+CD25- (Tconv) and CD4+CD25+ (Tregs) were cultured in RPMI high lactate & low glucose as adapted from^60^: RPMI 1640 medium, no glucose (Thermo Fisher) + 30 mg/dL D-(+)-Glucose (Sigma-Aldrich) + 20mM L-(+)-Lactic Acid (Sigma-Aldrich) + 1% P/S (Gibco)+ 10% FBS dialyzed (Thermo Fisher) + low-dose rhIL-2 (Gibco). As a control, RPMI standard was used: RPMI 1640 medium, no glutamine (Thermo Fisher) + 10% FBS (Gibco) + 1%P/S/Glutamine (Gibco) + 1% non-essential amino acids (Gibco). For apoptosis and viability assessments (outlined in the Flow Cytometry section), cells were cultured for 24 h.

A similar approach was used for the culture of WT and Nrf2^−/−^ T cells, although RPMI standard included sodium pyruvate (1%, Gibco), HEPES buffer (1%, Gibco) and 2-β-Mercaptoethanol (0.001%, Sigma-Aldrich). RPMI high lactate and low glucose also incorporated 2-β-Mercaptoethanol (0.001%, Sigma-Aldrich).

### In vitro evaluation of Nrf2-target gene expression in healthy human T cells cultured in lactic acid conditions, HCC and paired background liver homogenates

HCC and paired background HCC liver tissues were obtained from liver resections of treatment naïve HCC patients (*n* = 4 each). These tissues were sectioned into smaller fragments and immediately cryopreserved at −80 °C. Upon initiation of cell culture, the liver tissues were thawed and fully homogenized in the TissueLyser II using a single stainless-steel bead (Qiagen) along with RPMI 1640, no glutamine (Thermo Fisher). Subsequently, the homogenized tissue was passed through a 70 µM strainer and then appropriately diluted in RPMI 1640 medium (2.5 and 10 mg/ml).

For experiments conducted under lactic acid conditions, L-(+)-Lactic Acid was added at concentrations of 5 mM and 20 mM to the RPMI standard (as described above). To determine the gene expression of Nrf2-targets, human healthy T cells (CD8+, CD4+CD25- Tconv, CD4+CD25+ Tregs) were cultured for 4 h with anti-CD3/28 (1:5) and low-dose rhIL-2 (Gibco).

Each healthy human donor was cultured separately in distinct HCC and paired background homogenates to facilitate a comprehensive evaluation of the experimental conditions.

### MCT1 inhibition experiments

Freshly isolated human healthy CD4+CD25+ Tregs were pre-incubated in the presence or absence of 100 nM of the MCT1 inhibitor AZD3965 (MedChemExpress; HY-12750) for 2 h and then cultured with low dose IL-2 (50IU/ml) and 1:5 aCD3/CD28 beads in RPMI standard or RPMI high lactate and low glucose (as described above) for 30 min.

### Immunoprecipitation experiments

For experiments to define the molecular mechanisms involved in Nrf2 activation, 2 × 10^6^ cells were cultured in RPMI standard or RPMI high lactate and low glucose for 30 min, followed by 20 min in presence or absence of 2 μg/ml MG-132 (Sigma-Aldrich). Samples were lysed in a buffer solution of 10 mM HEPES (pH 7.9), 50 mM NaCl, 0.5 M Sucrose, 0.1 mM EDTA, 0.5% triton X-100, plus 1% fast protease inhibitor (S8820; Sigma-Aldrich) and 1% phosphatase inhibitor (P5726; Sigma-Aldrich). Protein lysate was precleared with Protein A/G PLUS-Agarose (SC-2003; Santa Cruz Biotechnology) for 2 h at 4 °C. Lysate and beads were separated by centrifugation, and the supernatant was incubated with KEAP1 Rabbit Polyclonal antibody (10503-2-AP, Proteintech) overnight at 4 °C. Then, 25% slurry of Protein A/G beads was added and incubated for 2 h at 4 °C. The antibody-bead conjugate was washed three times with wash buffer and boiled in lysing buffer plus Coomassie blue for 5 min at 81 °C.

### Western blotting experiments

Lysates were treated with 5 mM DL-Dithiothreitol (43819-5 g; Merck) for 5 min at RT. 10% of total lysate was loaded onto a Bolt 10% Bis-Tris Plus 15-well gel (NW00105BOX; Thermofisher Scientific), first resolved by gel electrophoresis and transferred to a nitrocellulose membrane. Membranes were blocked with 10% milk powder for 1 h at room temperature, then incubated with KEAP1 Rabbit Polyclonal Antibody (10503-2-AP, Proteintech) or Lactic acid Lysine Polyclonal Antibody (PA5-116901; Thermofisher Scientific) overnight at 4 °C. Then, membranes were washed three times with 0.1% tween20-TBS, incubated with goat anti-rabbit secondary antibody (401393-2 ML; Sigma-Aldrich) for 1 h at room temperature and washed three times with 0.1% tween 20-TBS. The film was developed manually by incubating for 2 min at room temperature with an enhanced chemiluminescence solution of milli-Q water, 1.25 mM luminol (A8511; Sigma-Aldrich), 0.2 mM p-Coumaric acid (C9008; Sigma-Aldrich), 100 mM Tris-HCl (pH 8.5), 7.5 mM H_2_O_2_ (386790-100 ML; Calbiochem) and dipped into trays containing XR D-6 NDT/XR D-1.5 NDT developer (25001180; Dürr NDT) and XR F-6 NDT/XR F-1.5 NDT fixer (25001181; Dürr NDT) for up to 2 min.

### Real-time PCR

Total RNA was isolated from Trizol-lysed samples by using the Direct-zol RNA MiniPrep or MicroPrep kits (ZymoResearch) according to the manufacturer’s instructions. A reverse-transcriptase reaction was performed using the High-Capacity cDNA Reverse Transcription Kit with RNase inhibitor (Applied Biosystems). Real-time PCR reactions were carried out with the TaqMan Universal MasterMix II, no UNG (Applied Biosystems) and TaqMan Gene Expression Assay Probes (Applied Biosystems; *ACTB* Hs01060665_g1 VIC, *NQO1* Hs01045993_g1 FAM, *GCLM* Hs00978072_m1 FAM, *HMOX1* Hs01110250_m1 FAM), *NFE2L2* Hs00975961_g1 FAM; *SLC16A1* (MCT1) Hs01560299_m1 FAM). To quantify the mRNA transcript levels, the expression of target genes was normalised to the housekeeping gene *ACTB*, and the results were shown as relative expression between cDNA of the treated sample and the paired untreated one. All qPCR experiments were performed in technical duplicates.

### Immunophenotyping of murine intra-tumoral leukocytes

Tissues were collected in ice-cold PBS. Tumours were digested using a mouse tumour dissociation kit in GentleMACS C digestion tubes with a GentleMACS tissue dissociator (Miltenyi Biotec) and single cell suspension generated by passing through a 70 μm filter. Cells were layered onto 30% Percoll gradient, and pelleted RBCs lysed. Cells were Live/Dead stained (L34962, ThermoFisher), Fc blocked (Clone 93) and then stained with the following fluorochrome-conjugated antibody panels: 1) Myeloid panel [Ly6C (Clone HK1.4), SIRPα (Clone P84), CD103 (Clone 2E7), CD45 (Clone 30-F11), CD64 (Clone X54-5/7.1), CD11b (Clone M1/70), CD11c (Clone N418), PD-L1 (Clone 10 F.9G2), XCR1 (Clone ZET), Ly6G (Clone 1A8), CD86 (Clone PO3), MHC-II (Clone M5/114.15.2), F4/80 (Clone T45-2342), VLA4 (Clone GC10 (MFR4.B))]; 2). Lymphoid panel [LAG3 (Clone C9B7W), EOMES (Clone W17001A), PD-1 (Clone RMP1-30), CTLA-4 (Clone UC10-4F10-11), CD8 (Clone 53-6.7), Granzyme B (Clone QA16A02), CD4 (Clone RM4-5), FoxP3 (Clone MF-14), TIM3 (Clone RMT3-23), CD62L (Clone MEL-14), CXCR6 (Clone SA051D1), NK1.1 (Clone PK136), CD3 (Clone 17A2), CD44 (Clone IM7), gdTCR (Clone GL3), CD28 (Clone 37.51), CD25 (Clone PC61), CD19 (Clone 1D3)]. Intracellular stainings were performed using the eBioscience™ Foxp3/Transcription Factor Staining Buffer Set (00-5523-00, Invitrogen).

All experiments were performed using a BD Symphony A5 flow cytometer using BD FACSDiveTM Diva software. Data were analysed using FlowJo software version 10.7.1.

### siRNA-mediated Nrf2 gene silencing in human Tregs

Human Tregs were transfected with siRNA using the Accell delivery system (Dharmacon, Horizon Discovery) to achieve targeted Nrf2 knockdown. Cells were seeded at 1 × 10⁶ cells per well in a 24-well plate and cultured in Accell Delivery Media supplemented with low-dose recombinant human IL-2 (50 U/ml) and anti-CD3/CD28 activation beads (1:5 bead-to-cell ratio, Gibco). SMARTpool siRNAs targeting human *NFE2L2* (Nrf2) and control siRNAs from Accell (see the Source Data file for detailed information) were added at a final concentration of 1 μM. Transfection was performed using debeaded cells, and siRNAs were added directly to the culture without the need for transfection reagents. After 48 h, cells were harvested and split into treatment conditions using RPMI medium supplemented with low dose IL-2 (50 U/ml), with or without L-(+)-Lactic Acid (20 mM, Sigma-Aldrich). Cells were cultured for an additional 24 h before downstream analyses.

### HCC tissue cytokine measurements

We employed MSD Multi-spot V-Plex sandwich immunoassay kits with the following panels: Proinflammatory Panel 1 Human Kit [granulocyte-macrophage colony-stimulating factor (GM-CSF), IL-1a, IL-5, IL-7, IL-12/23p40, IL-15, IL-16, IL-17a, Tumour necrosis factor-b (TNFb) and vascular endothelial growth factor (VEGFa)]; Cytokine Panel 1 Human Kit [Interferon-g (IFNg), IL-1b, IL-2, IL-4, IL-6, IL-8 (CXCL8), IL-10, IL-12p70, IL-13 and TNFa]. 96-well plates were pre-coated with capture antibodies on independent well-defined spots according to the manufacturer’s instructions and read using MESO QuickPlex SQ 120.

### HCC tissue gene expression (RNA-Seq)

RNA samples from cryopreserved liver biopsies were prepared by TRIzol extraction (Thermo Fisher), and the quantity, purity, and integrity of the samples were measured with Nanodrop (Thermo Scientific) and Bioanalyzer (Agilent). RNA libraries were prepared using the NEBNext Ultra II Directional RNA Library Prep kit for Illumina (NEB) and sequenced using Illumina NovaSeq 6000 System by Azenta L.T.D.

### Ethics

The study was conducted in accordance with the Declaration of Helsinki. The study involved animals, and all procedures related to animal work were performed in accordance with all legal, ethical, and institutional requirements (PP4067431 and PP8854860) as dictated by UK legislation and ethical review committees at King’s College London and Newcastle University. Sample sizes of in vivo experiments were based on prior work from our laboratories and kept to a minimum in line with law and ethical guidelines for animal research in the UK, as were the in vivo endpoints. For reporting, we apply ARRIVE 2.0 guidelines. (https://www.nc3rs.org.uk/arrive-guidelines).

All mice were housed in specific pathogen free conditions with unrestricted access to food and water and maintained on a constant 12 h light-dark cycle under controlled climate.

### Statistics and reproducibility

Statistical significance was determined using GraphPad Prism 8 (GraphPad Software). The statistical tests performed for each experiment are indicated in the figure legends. Normally distributed data were summarised as mean and standard deviation (SD). For the statistical analysis of two groups, a two-sample unpaired or paired *t*-test was performed. For analysis of multiple groups, one-way analysis of variance (ANOVA) with Tukey’s multiple comparisons test was applied. For multiple testing, a two-way ANOVA with Bonferroni’s multiple comparisons test was applied.

Phenotyping of clinical samples was performed as samples were received. Microsuppression assays were conducted in a single experiment, with at least three technical replicates per biological sample. ScRNA-seq was performed in batches of four biological replicates, ensuring that paired samples were always processed together. CyTOF analyses were carried out in batches of six biological replicates, with a control PBMC sample included in each batch. In vitro assays were performed in batches of four biological replicates, and in vivo studies were conducted as a single experiment.

### Reporting summary

Further information on research design is available in the Nature Portfolio Reporting Summary linked to this article.

## Supplementary information

Supplementary Information

Description of Additional Supplementary Files

Supplementary Data 1-9

Reporting Summary

Transparent Peer Review file

## Source data

Source data

## Data Availability

All data supporting the findings of this study are available within the paper and its supplementary information. Multi-omics single-cell data generated in this study has been deposited in the NCBI Gene Expression Omnibus (GEO) under accession number GSE320155. Processed and annotated scanpy objects can be downloaded from a Zenodo repository. GSE link: https://www.ncbi.nlm.nih.gov/geo/query/acc.cgi?acc=GSE320155; Zenodo link: 10.5281/zenodo.15471231. Source data are provided with this paper.
