# Supplementary material for: Nrf2-mediated metabolic reprogramming drives regulatory T cell accumulation in hepatocellular carcinoma
Source: Nat Commun. 2026 Jul 13;17:8639. doi: 10.1038/s41467-026-73485-3 (PMC13487165; doi:10.1038/s41467-026-73485-3)
Supplement: Supplementary file 1 [file 41467_2026_73485_MOESM1_ESM.pdf]

Supplementary Information

TITLE: Nrf2-mediated metabolic reprogramming drives regulatory T cell accumulation in hepatocellular carcinoma

AUTHOR LIST: Perpiñán E[1], Sompairac N[2,3+], Marin Correa D[1+], Ramon-Gil E[4,5], Man RCH[6], Camell-Raventos D[7], Savoldelli R[1], Fleming M[1], Bellersheim L[1], Santos CX[8], Elgosbi M[1], Lozano JJ[9], Maddaloni M[10], Schmidl C[10], Kodela E[1], Piqué-Gili M[7], de Sande AH[7], Pinyol R[7], Higginbotham S[4,5], Peng Q[1], Landman E[1], Torres-Yaguana J[1], Sharma V[1], Zhang C[1], Kurt A[1], Liao X[11], Dowe T[1], Habarwaa M[1], Liu Z[6], Zen Y[1], Miquel R[1], Sarker D[2,12], Kordasti S[2,3], Tree T[13], Shah AM[8], Fruhwirth GO[6], Dinkova-Kostova AT[14], Pearce EL[15,16], Leslie J[4,5], Llovet JM[7,17,18], Sánchez-Fueyo A[1†], Safinia N[1†*]

*Corresponding author. Email: niloufar.1.safinia@kcl.ac.uk

+ These authors contributed equally. † These authors jointly supervised this work.

AFFILIATIONS:

[1]Roger Williams Institute of Liver Studies, School of Immunology & Microbial Sciences, Faculty of Life Sciences and Medicine, King's College London University, and King's College Hospital, London, UK.

[2]Comprehensive Cancer Centre, School of Cancer and Pharmaceutical Sciences, Faculty of Life Sciences and Medicine, King's College London, London, UK.

[3]Haematology Department, Guy's Hospital, London, UK.

[4]Newcastle Fibrosis Research Group, Biosciences Institute, Faculty of Medical Sciences, Newcastle University, Newcastle Upon Tyne, UK.

[5]The Newcastle University Centre for Cancer, Newcastle University, Newcastle upon Tyne, UK.

[6]Imaging Therapies and Cancer Group, Comprehensive Cancer Centre, School of Cancer and Pharmaceutical Sciences, King's College London, London, UK.

[7]Liver Cancer Translational Research Group, Institut d'Investigacions Biomèdiques August Pi i Sunyer (IDIBAPS), Liver Unit. Hospital Clínic, Universitat de Barcelona, Barcelona, Catalonia, Spain.

[8]King's College London British Heart Foundation Centre, School of Cardiovascular & Metabolic Medicine and Sciences, London, UK.

[9]CIBER of Hepatic and Digestive Diseases (CIBERehd), Barcelona, Spain.

[10]Leibniz Institute for Immunotherapy (LIT), Regensburg, Germany.

[11]Department of Protein Science, Science for Life Laboratory, KTH-Royal Institute of Technology, Stockholm, Sweden.

[12]Department of Medical Oncology, King's College Hospital, London, UK.

[13]Peter Gorer Department of Immunobiology, King's College London, London, UK.

[14]Jacqui Wood Cancer Centre, Division of Cancer Research, School of Medicine, University of Dundee, Dundee, UK.

[15]Bloomberg Kimmel Institute of Cancer Immunotherapy, Department of Oncology, Johns Hopkins University School of Medicine, Baltimore, MD, USA.

[16]Department of Biochemistry and Molecular Biology, Johns Hopkins Bloomberg School of Public Health, Baltimore, MD, USA.

[17]Mount Sinai Liver Cancer Program, Division of Liver Diseases, Department of Medicine, Tisch Cancer Institute, Icahn School of Medicine at Mount Sinai, New York, USA.

[18]Institució Catalana de Recerca i Estudis Avançats (ICREA), Barcelona, Catalonia, Spain.

Supplementary Figures

Supplementary Fig. 1

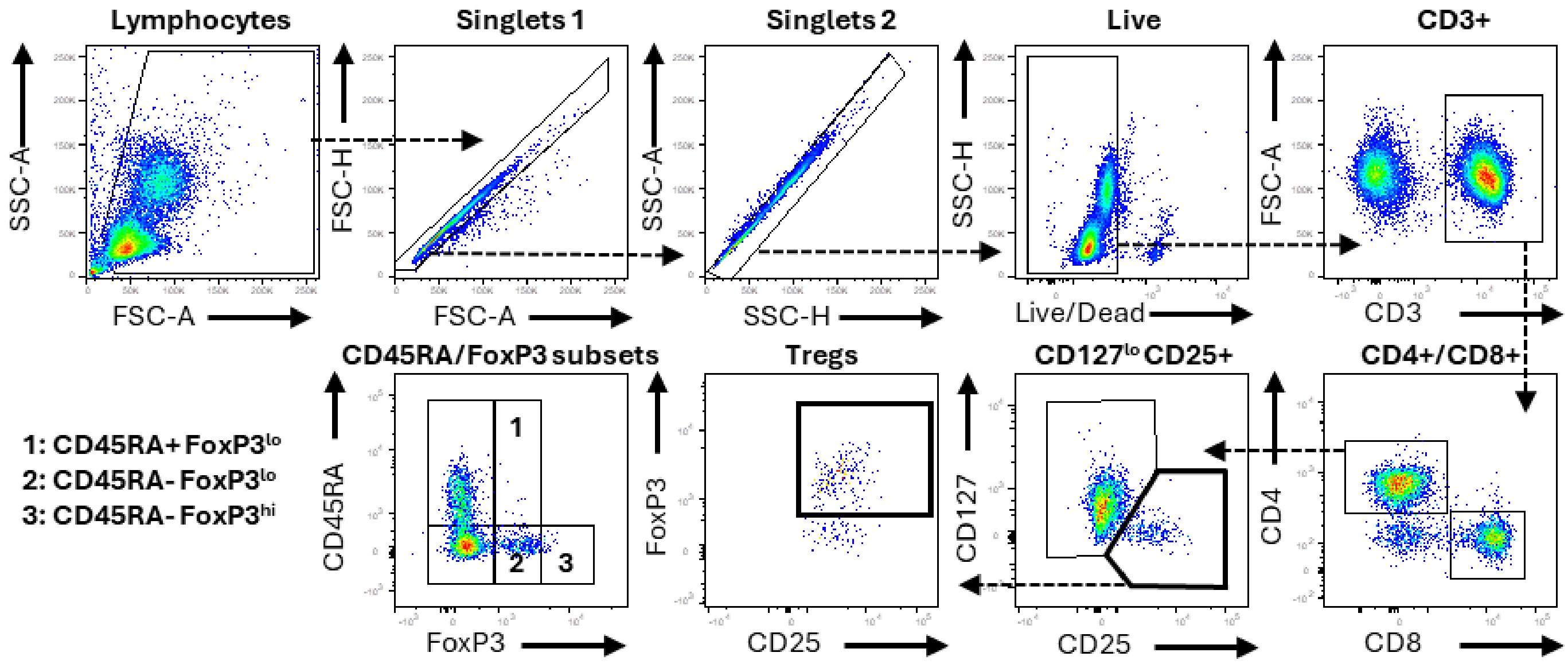

Supplementary Fig. 1. Flow cytometry gating strategy for the identification and characterization of human Tregs. Gating strategy used for Figures 1, 5h-i, 6d-i and 6o. lymphocytes were first gated based on forward scatter area (FSC-A) and side scatter area (SSC-A). Singlets were selected using FSC-A vs. FSC-H and SSC-H vs. SSC-A plots. Live cells were identified using viability dye. $CD3^+$ T cells were gated, followed by selection of $CD4^+$ and $CD8^+$ subsets. Tregs were defined as $CD4^+CD127^{lo}CD25^+$ cells. Within the CD4+ gate, three subsets were distinguished based on CD45RA and FoxP3 expression: (1) $CD45RA^+$ $FoxP3^{lo}$, naïve/resting Tregs; (2) $CD45RA^-$ $FoxP3^{lo}$, cytokine-secreting Tregs; (3) $CD45RA^-$ $FoxP3^{hi}$, activated/effector Tregs. All gates were set based on fluorescence minus one controls. The antibodies used are listed in Supplementary Table 16 (Human Treg count).

Supplementary Fig. 2

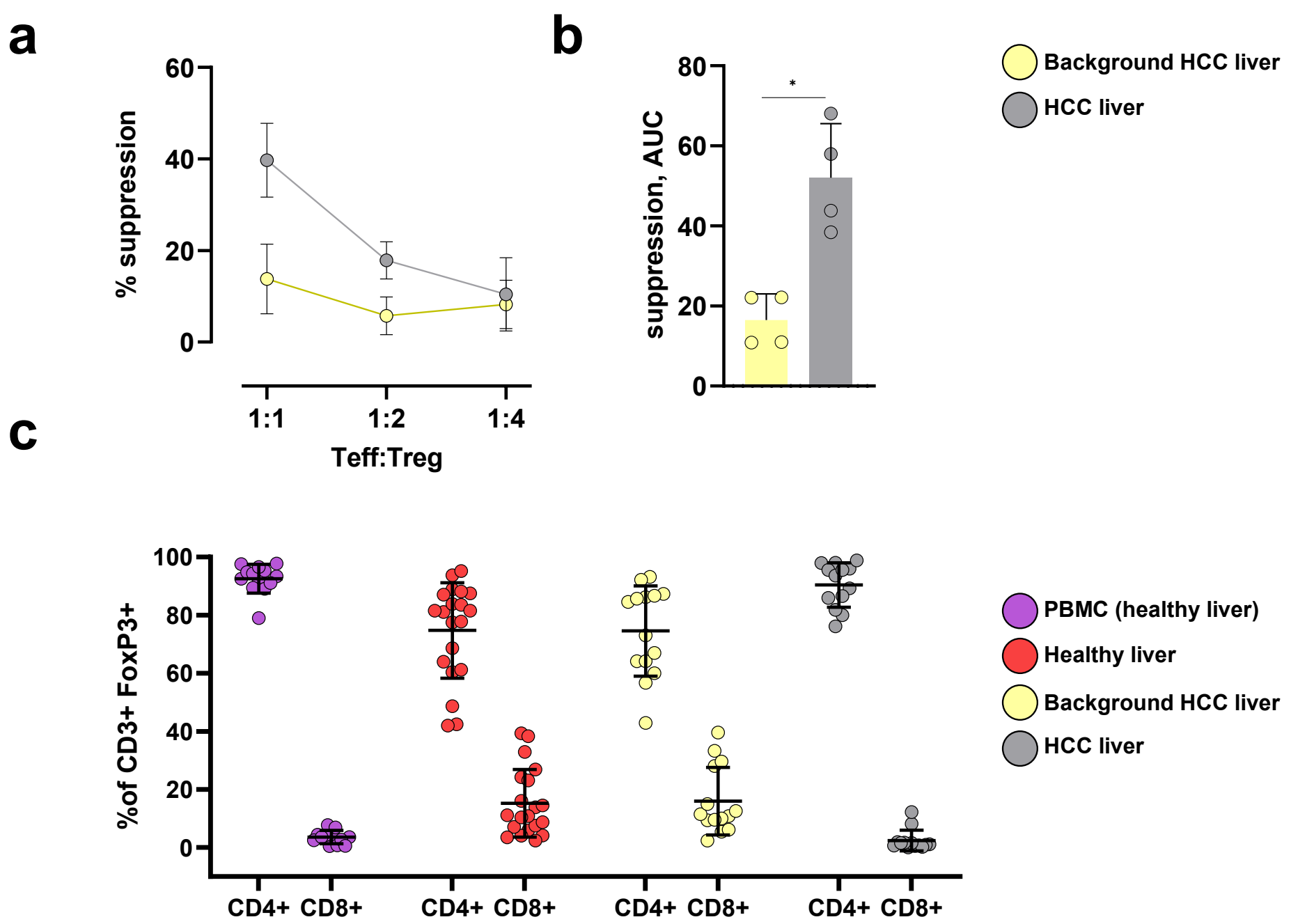

Supplementary Fig. 2. Functional characterization of Tregs in an autologous system and analysis of CD8^{+} FoxP3^{+} cells across liver compartments and peripheral blood

(a) Determination of Treg suppression, represented as the percentage of suppression of third-party CD4+ Teff, for each Treg:Teff ratio (n=4 samples for each ratio), and **(b)** Treg suppressive capacity calculated by integrating the percentage of suppression for each ratio in HCC and paired background tissues. **(c)** Frequency of CD4+ and CD8+ cells within the CD3+ FoxP3+ subset in different compartments. P-value was calculated by One-way ANOVA with Tukey's multiple comparisons test. Mean ±SD are shown. Only statistically significant P-values are displayed: *, P<0.05; **, P<0.01; ***, P<0.001; ***, P<0.0001. AUC, area under the curve.

Supplementary Fig. 3

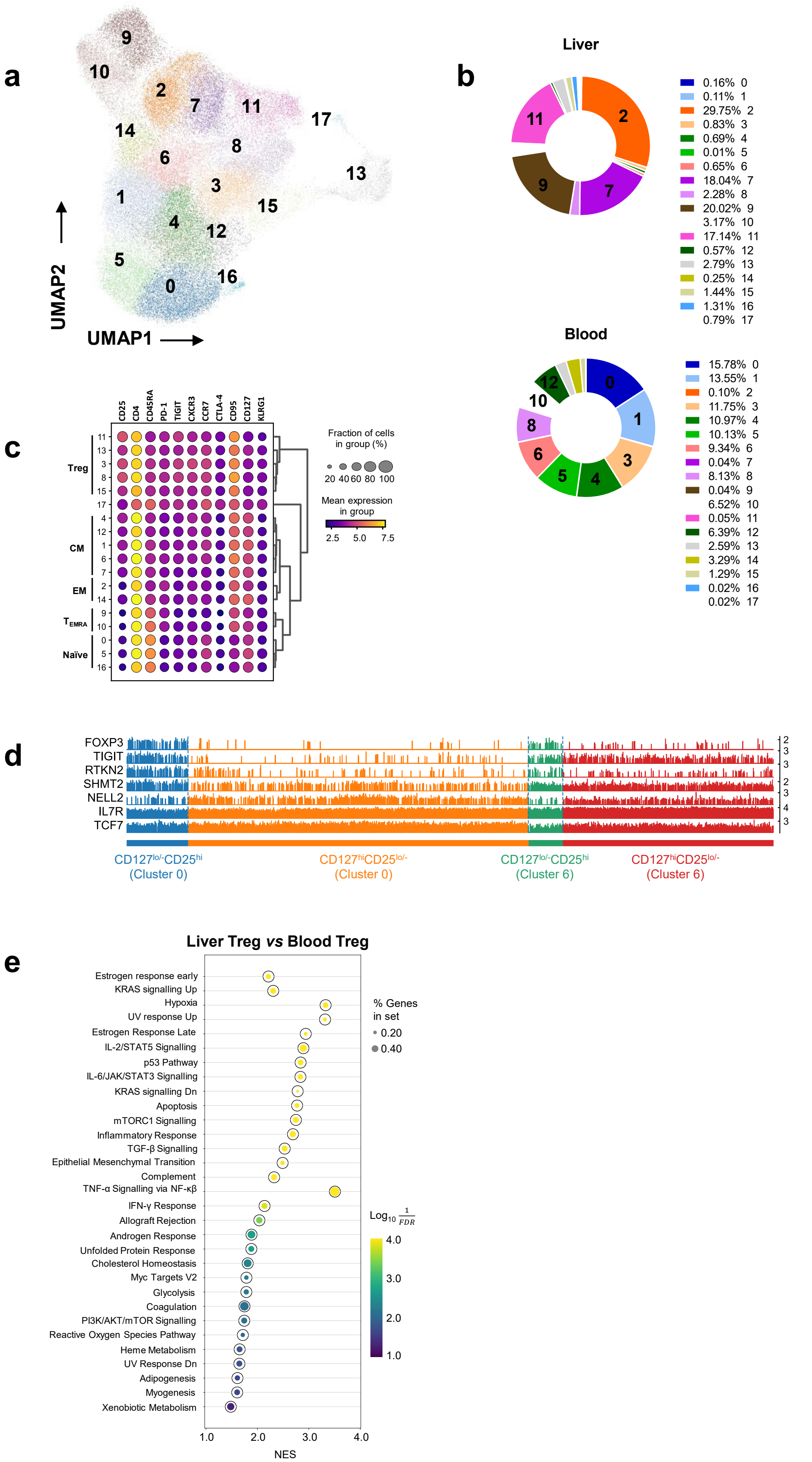

Supplementary Fig. 3. ScRNA-seq analysis of Treg and Tconv CD4+ T cells from healthy liver tissue and paired PBMCs. (a) UMAP of single cell transcriptomes of CD4+ T cells from liver and PBMCs (n=5 each), colored by subsets. **(b)** Distribution of cell clusters within CD4+ T cells from liver and PBMCs. **(c)** Mean protein surface expression on CD4+ T cell clusters assessed by scRNA-seq CITE-seq technology. **(d)** Track plot showing expression of *FOXP3*, *TIGIT*, *RTKN2*, *SHMT2*, *NELL2, IL7R*, and *TCF7* in $CD127^{lo/-}CD25^{hi}$ and $CD127^{hi}CD25^{lo/-}$ cells from CD4+ Tconv clusters 0 and 6. $CD127^{lo/-}CD25^{hi}$ cells, termed "furtive" Tregs, constituted 15% and 6% of blood-derived Tconv clusters 0 and 6, and were absent in the liver. **(e)** Gene set enrichment analysis (GSEA) of key Hallmark pathways comparing liver Tregs and circulating Tregs. The horizontal axis represents the normalized enrichment score (NES), while the vertical axis represents the enriched pathway name. The color scale indicates different false discovery rate (FDR) thresholds, and circle size denotes the number of genes per pathway.

Supplementary Fig. 4

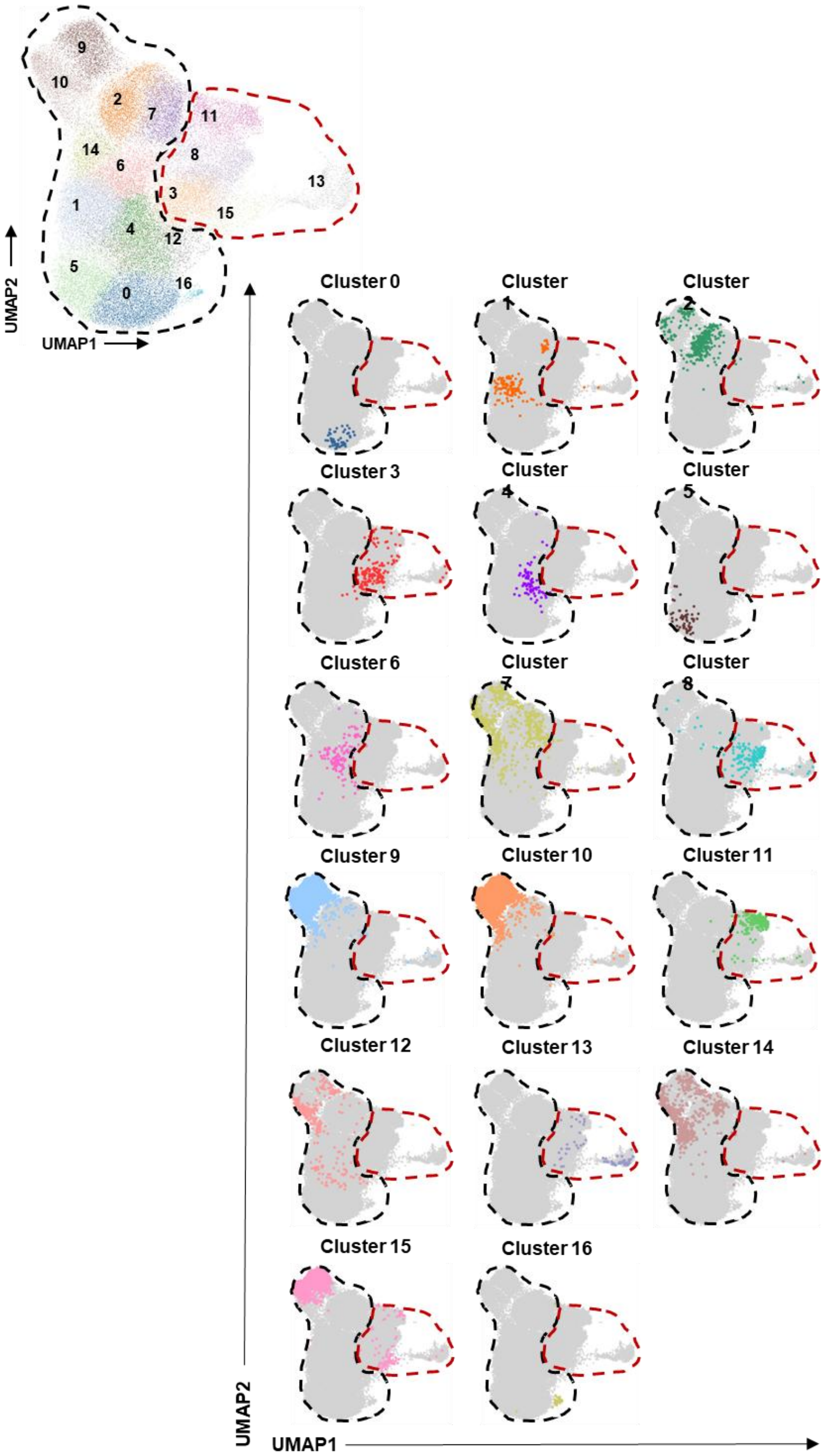

Supplementary Fig. 4. The origin of liver resident Tregs does not involve the differentiation of intra-hepatic Tconv cells. UMAP visualizations depicting the top 25 most prevalent TCR clonotypes within each CD4+ T cell cluster. The inset UMAP displays the spatial distribution of Tconv clusters (0, 1, 2, 4, 5, 6, 7, 9, 10, 12, 14, and 16) and Treg clusters (3, 8, 11, 13, and 15), with dashed lines demarcating Tconv (black) and Tregs (red) zones.

Supplementary Fig. 5

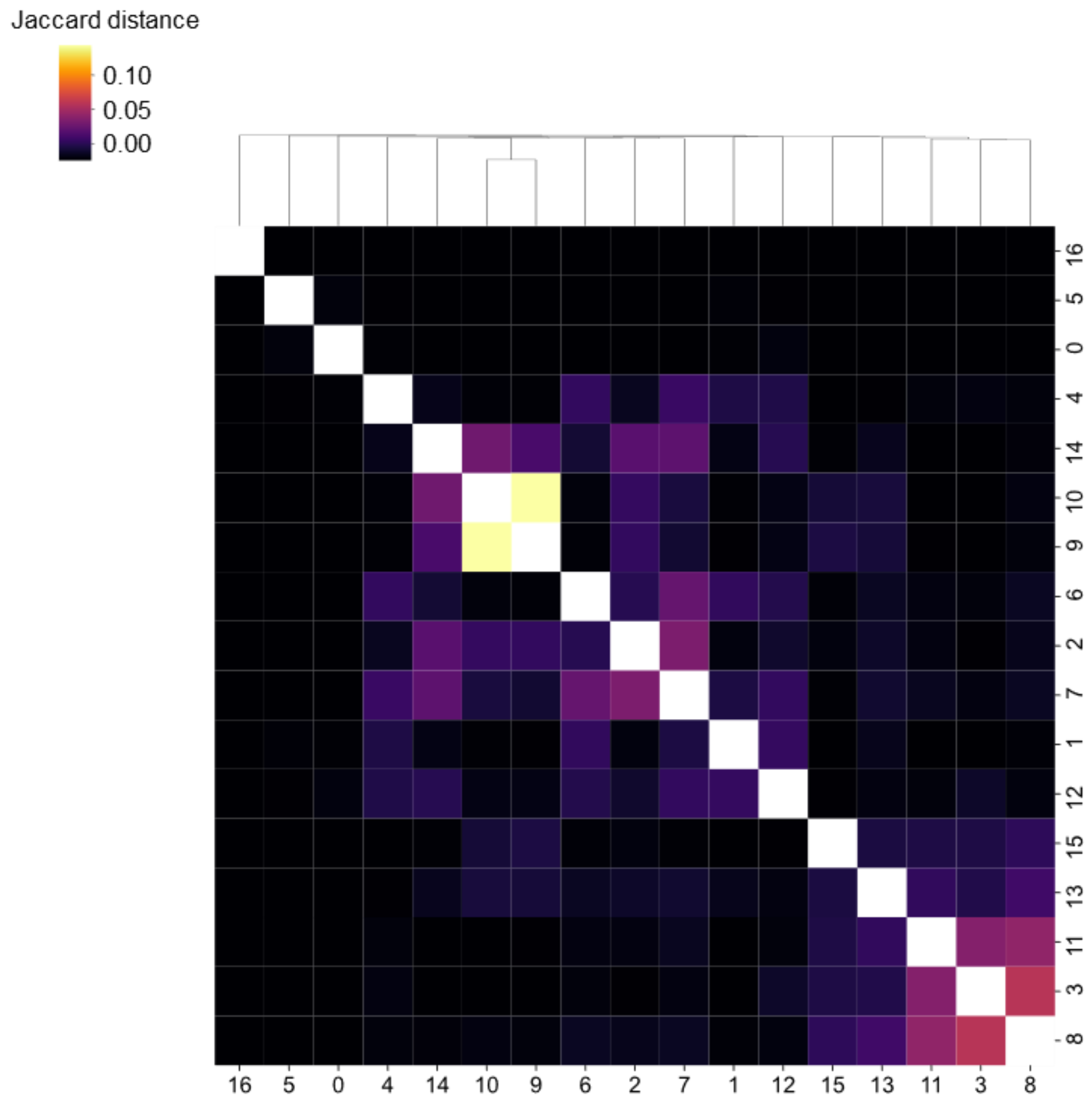

Supplementary Fig. 5. TCR repertoire overlap between the different CD4+ T cell clusters. Heatmap showing the Jaccard distance matrix of CD4+ T cell clusters as well as their linkage by hierarchical clustering. The Jaccard distance assesses TCR clonal overlap between CD4+ clusters via pairwise comparisons. Colour intensity reflects Jaccard distance values, with darker shades indicating lower and lighter shades indicating higher TCR repertoire overlap.

Supplementary Fig. 6

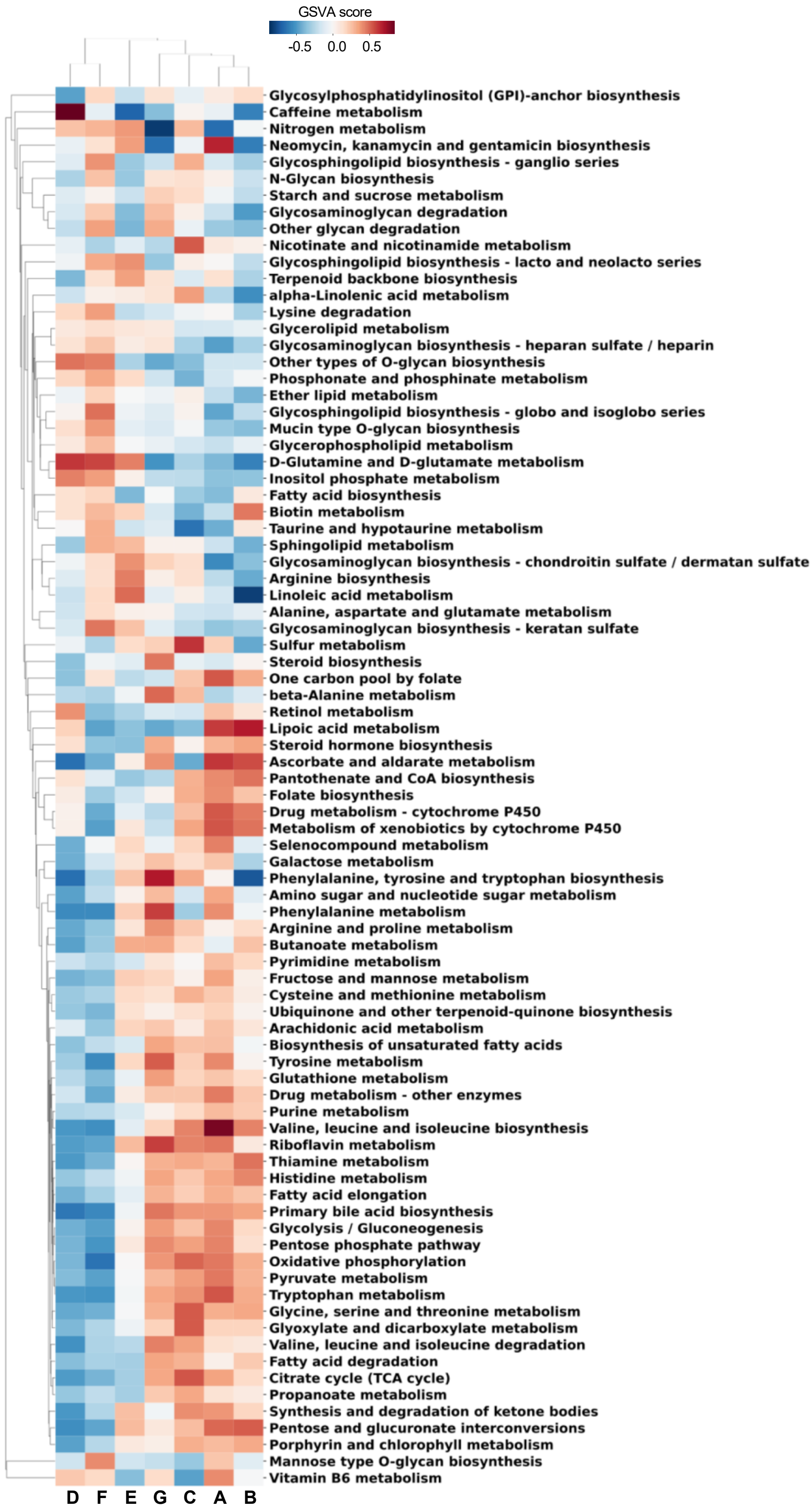

Supplementary Fig. 6. Metabolic characterization of Treg subsets in blood and healthy liver. Heatmap showing the GSVA enrichment score of KEGG metabolic pathways for each Treg cluster. The colour scale depicts the GSVA scores ranging from low (blue) to high (red) for each pathway within each cluster. Hierarchical clustering illustrates clusters with similar GSVA scores and their corresponding pathways.

Supplementary Fig. 7

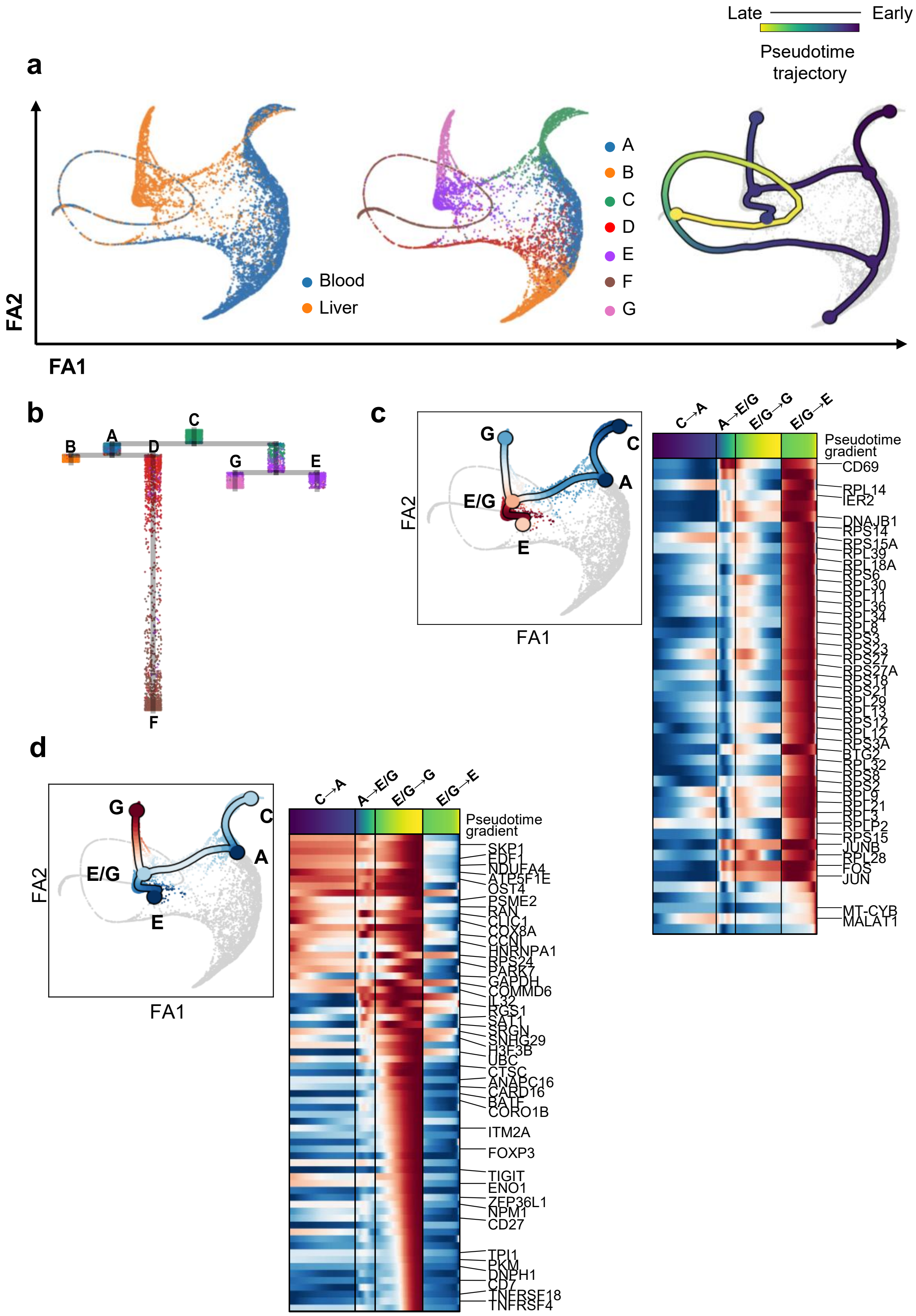

Supplementary Fig. 7. Pseudotime analysis of Treg-like cells from paired healthy liver and blood compartments, integrated using Harmony. (a) Diffusion maps of Treg-like cells integrated using Harmony, visualized along FA1 and FA2 axes. Cells are colored by tissue compartment (blood in blue, liver in orange; left), by originally identified Treg clusters (A–G; middle), and by pseudotime trajectory inferred using cluster C as the root (right). Pseudotime is projected onto the learned tree structure and visualized using the viridis color scale. **(b)** Dendrogram representation of the pseudotime trajectory, with cells colored according to their original Treg cluster identity, illustrating the inferred progression across cellular states. (**c**) Pseudotime bifurcation analysis depicting the average expression of the differentially expressed genes in cluster E along the tree (left) and a heatmap of the differentially expressed genes in cluster E [top 40 displaying the strongest amplitude] (right). (**d**) Pseudotime bifurcation analysis depicting the average expression of the differentially expressed genes in cluster G along the tree (left) and heatmap of the differentially expressed genes in cluster G [top 40 displaying the strongest amplitude] (left).

Supplementary Fig. 8

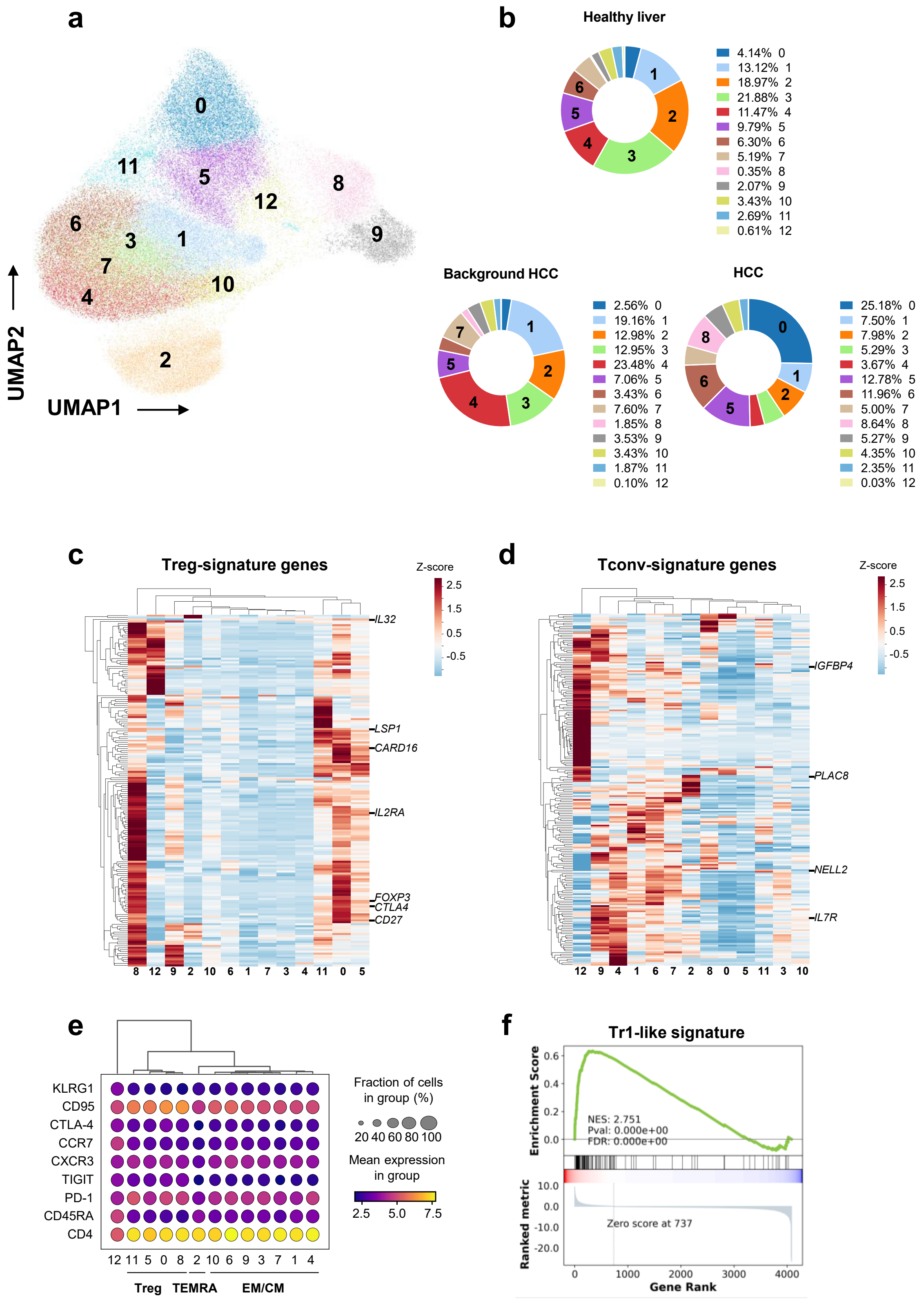

Supplementary Fig. 8. Characterization of CD4+ T cell subsets and identification of Tregs by scRNA-seq in different liver compartments. (a) UMAP of single cell transcriptomes of CD4+ T cells isolated from healthy liver, HCC and paired background HCC tissues (n=5 of each), coloured by subsets. **(b)** Distribution of cell clusters within the CD4+ T cell population isolated from healthy liver, HCC and paired background HCC tissues. **(c, d)** Heatmaps showing the expression of the Treg- and T-conv gene signatures, respectively, within each CD4+ T cell cluster. The colour scale indicates the Z-score level from low (blue) to high (red) expression. Hierarchical clustering illustrates clusters with similar gene expression and their corresponding genes. **(e)** Dot plot showing the mean protein surface expression on the different CD4+ T cell clusters assessed by scRNA-seq CITE-seq technology. The size of each circle is proportional to the percentage of cells expressing the maker, and the colour scale depicts the mean expression level of the marker from low (purple) to high (yellow). Hierarchical clustering shows clusters with similar protein surface expression. **(f)** Gene Set Enrichment Analysis (GSEA) plot corresponding to the Tr1-like reference gene signature for cluster 2 (NES, normalized enrichment score; FDR, false discovery rate; Pval, P-value).

Supplementary Fig. 9

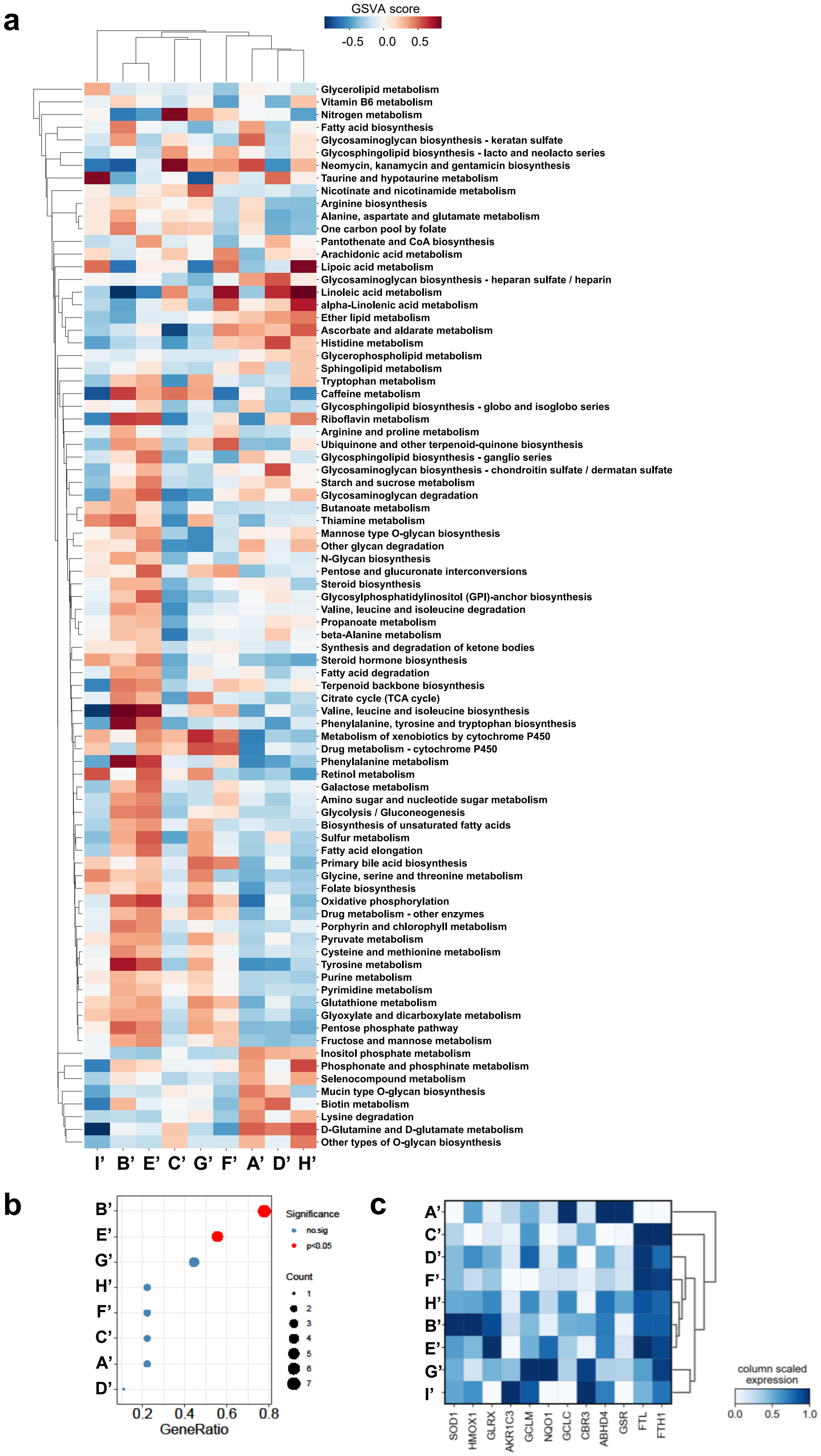

Supplementary Fig. 9. Metabolic characterization of Treg subsets in different liver compartments (a) Heatmap showing the GSVA enrichment score of KEGG metabolic pathways for each Treg cluster in healthy liver, HCC and paired background HCC tissues. The colour scale depicts the GSVA scores ranging from low (blue) to high (red) for each pathway within each cluster. Hierarchical clustering illustrates clusters with similar GSVA scores and their corresponding pathways. **(b)** Dot plot showing the ORA enrichment analysis of the Nrf2-target genes (*AKR1C3*, *OSGIN1*, *SLC7A11*, *GCLM*, *SRXN1*, *NQO1*, *GCLC*, *CBR3*, *ABHD4*, *PIR*, *GSR*, *FTL*, *ME1*, *FTH1*, *EPHX1*, *G6PD*, *GLRX*, *GPX2*, *HDK1*, *HMOX1*, *PRDX1*, *PRDX6*, *TXN1*, *TXNRD1* and *PPARG*) across Treg clusters. The dot size indicates the percentage of cells within each cluster expressing these genes, while the colour intensity denotes statistical significance. **(c)** Heatmap showing the expression of key Nrf2-target genes in each Treg cluster. The expression of each gene is scaled by the average and depicted by the colour intensity from low (light) to high (dark) expression.

Supplementary Fig. 10

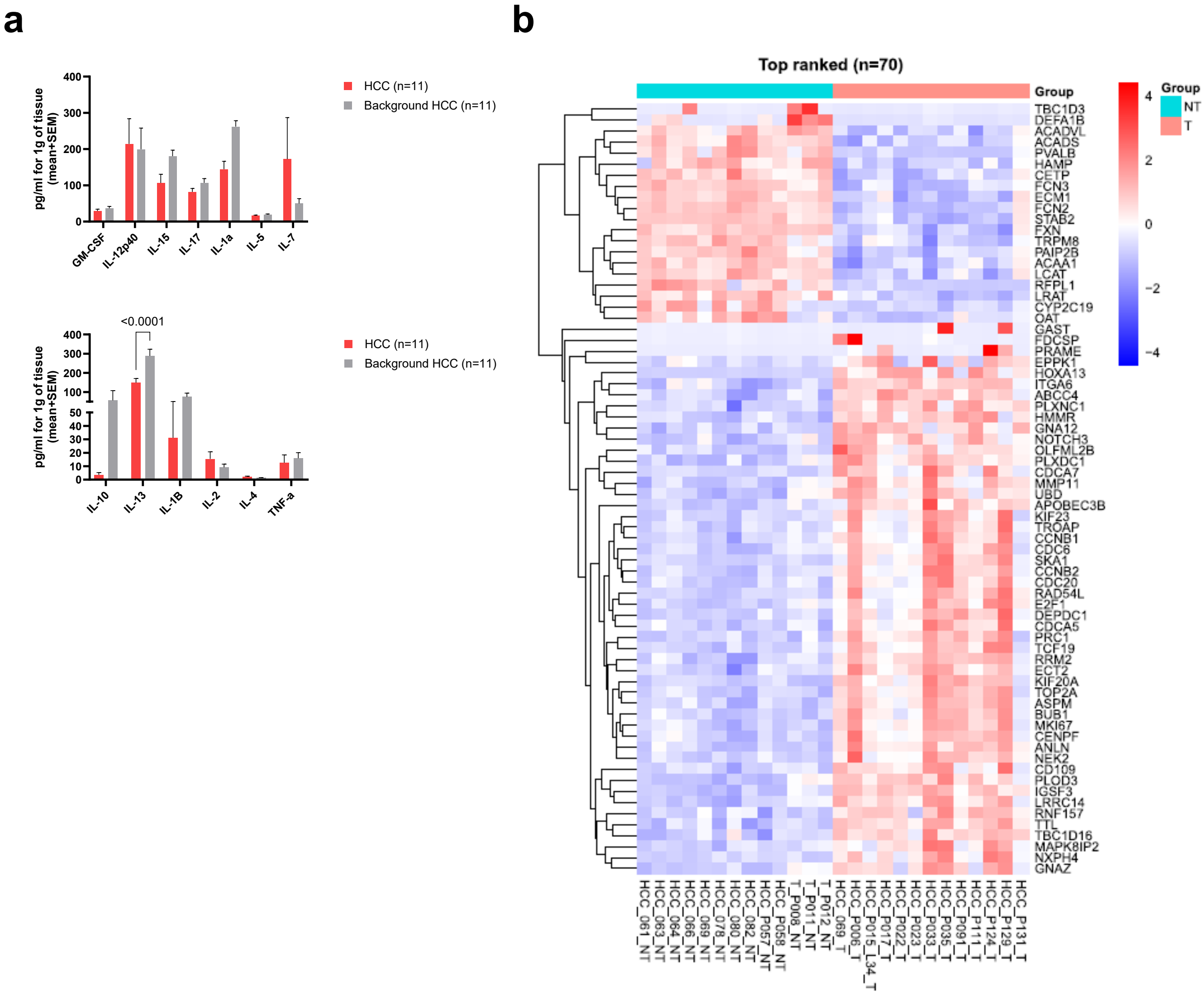

Supplementary Fig. 10. Proteomic and gene expression analysis of HCC and paired background tissues. (a) Bar graphs showing expression levels of selected cytokines in HCC and paired background tissues (n=11, each). P-value was calculated by two-way ANOVA with Sidak's multiple comparisons test. Mean +SEM are shown. Only statistically significant P-values are displayed. **(b)** Heatmap of the top 70 ranked genes based on differential expression across sample groups. Each row represents a gene, and each column a sample. Expression levels are colour-coded from low (blue) to high (red). Sample groups are indicated above the heatmap: T, HCC, NT, background HCC.

Supplementary Fig. 11

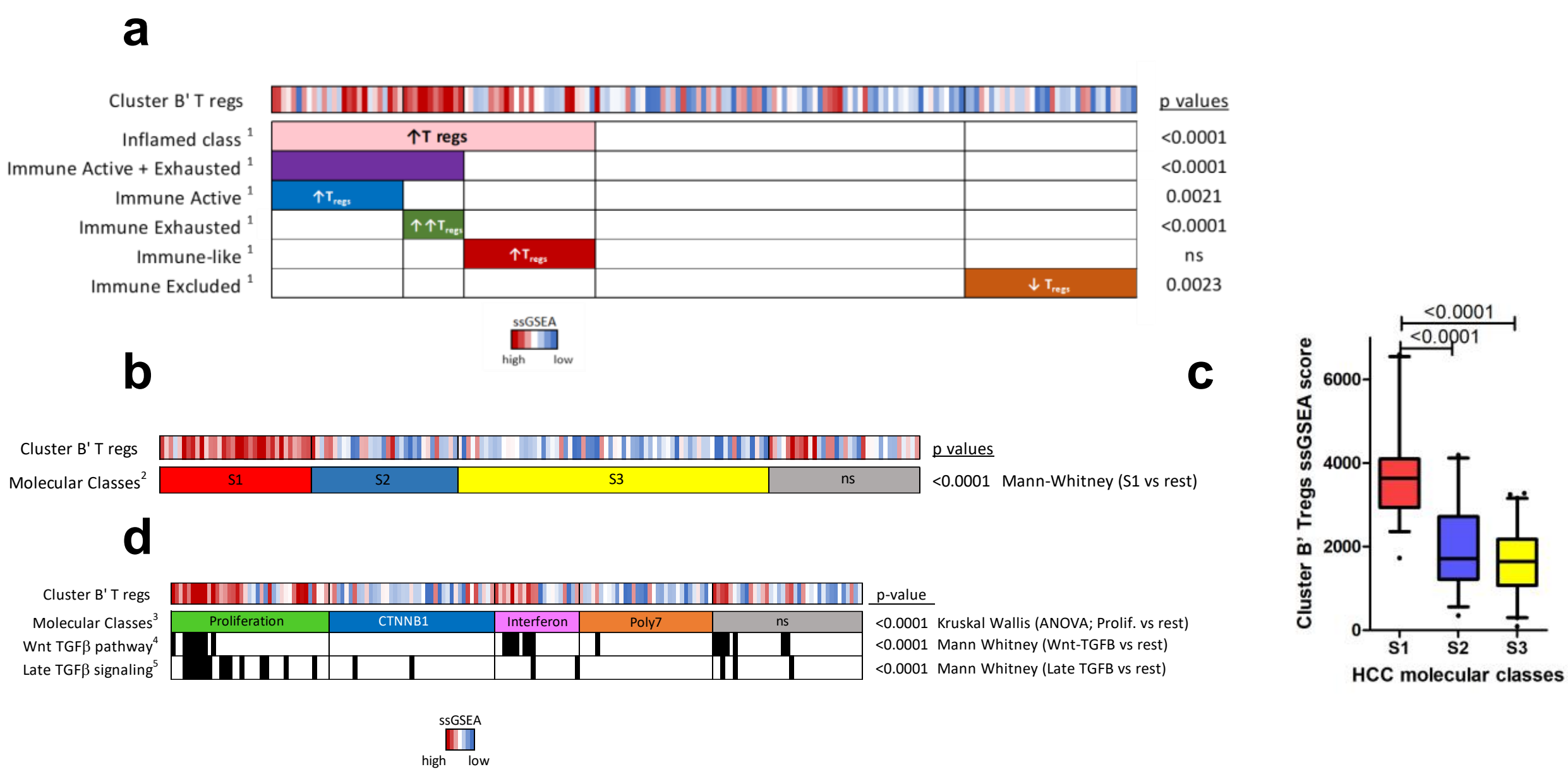

Supplementary Fig. 11. Correlation of Cluster B' gene signature with signatures derived from specific molecular and immune HCC subtypes. (a) Cluster B' Tregs are enriched in the Inflamed class and Immune class of HCC, especially Immune Exhausted tumors, while excluded from the Immune Excluded class. **(b, c)** Cluster B' Tregs are enriched in the S1 class. **(d)** Cluster B' Tregs are enriched in the Proliferation class, excluded from CTNNB1 and Poly7 classes, and correlate with activation of Wnt-TGFB pathway and Late TGFB activation. Mann-Whitney t-test was used for statistical analysis.

Supplementary Fig. 12

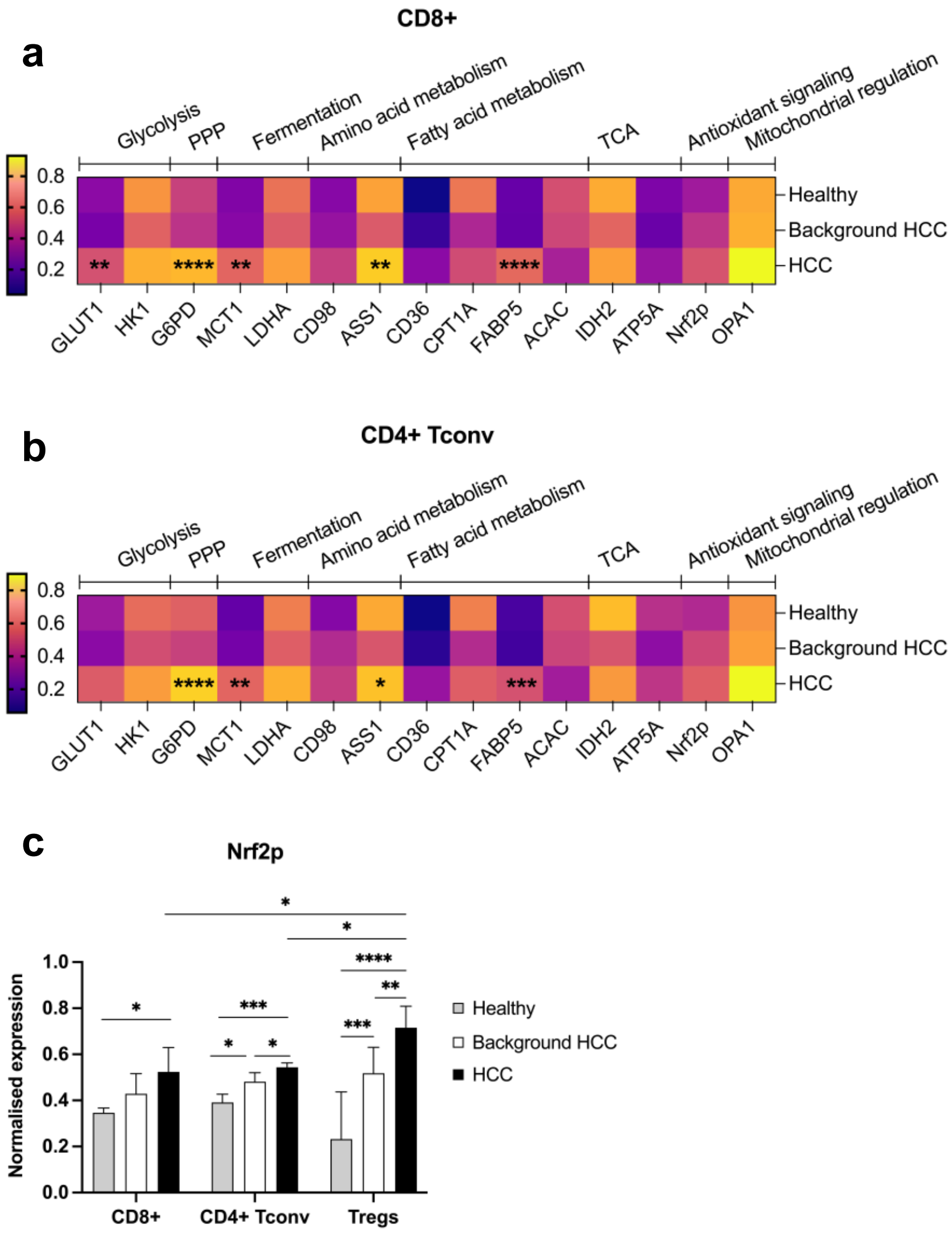

Supplementary Fig. 12. Metabolic profile of liver resident Tconv. (a, b) Heatmaps showing the normalized expression of key metabolic regulators in CD8+ and CD4+ Tconv cells, respectively, isolated from healthy liver, HCC and paired background HCC liver (n=5 of each). P-value shows the paired comparison between TIL T cells and T cells isolated from the paired background HCC tissue. **(c)** Normalised expression of Nrf2p in CD8+, CD4+ Tconv and Tregs in the different liver compartments (n=5 samples of each). P-value was calculated by two-way ANOVA with Tukey's multiple comparisons test. Only statistically significant P-values are displayed: *, P<0.05; **, P<0.01; ***, P<0.001; ****, P<0.0001. PPP, pentose phosphate pathway; TCA, tricarboxylic acid cycle.

Supplementary Fig. 13

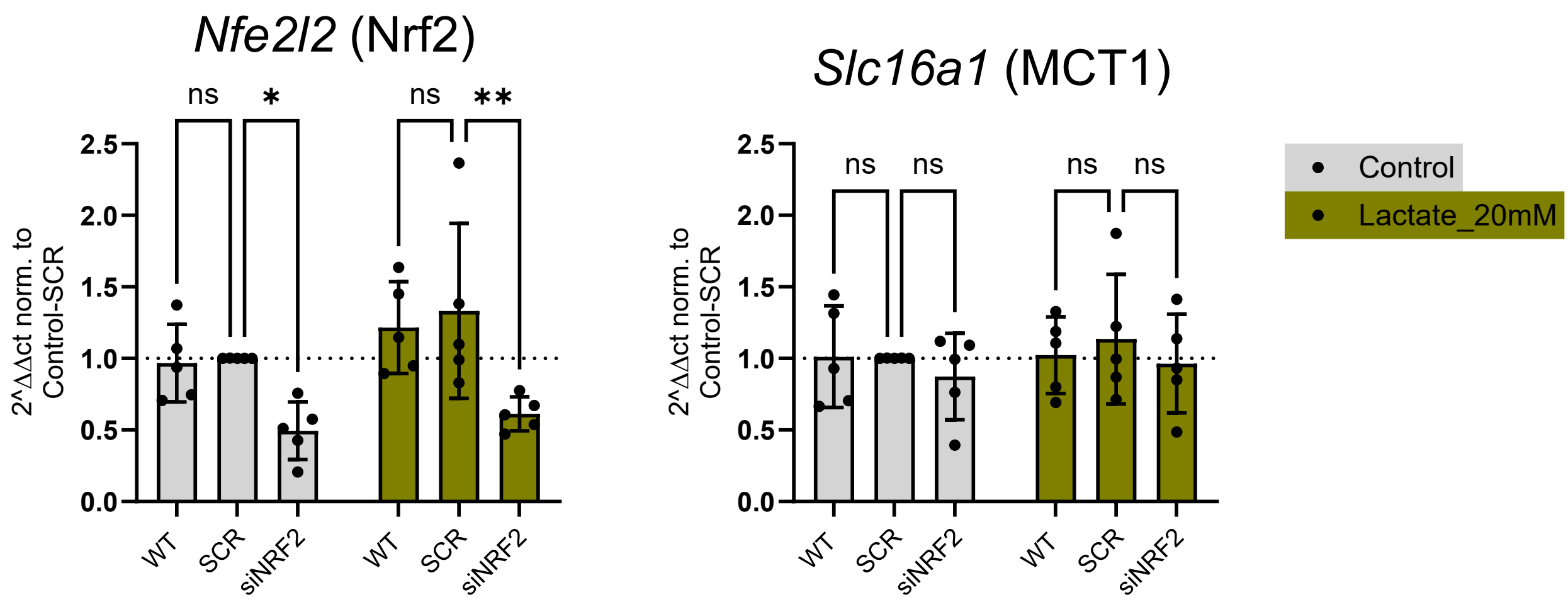

Supplementary Fig. 13. Nrf2 knockdown does not alter MCT1 expression in human Tregs. Relative mRNA expression levels of *Nfe2l2* (Nrf2) and *Slc16a1* (MCT1) in human Tregs, normalized to control. Tregs were freshly isolated using magnetic beads and expanded for 7 days with anti-CD3/CD28 beads (bead-to-cell ratio 1:1), rapamycin (100 nM), and recombinant human IL-2 (50 U/mL). Cells were then either left untransfected (wild-type, WT), transfected with a control plasmid (SCR), or with siRNA targeting *Nfe2l2* (siNRF2). After 48 hours of culture in standard RPMI medium with or without 20 mM lactate, qPCR was performed to assess gene expression.

Supplementary Fig. 14

a

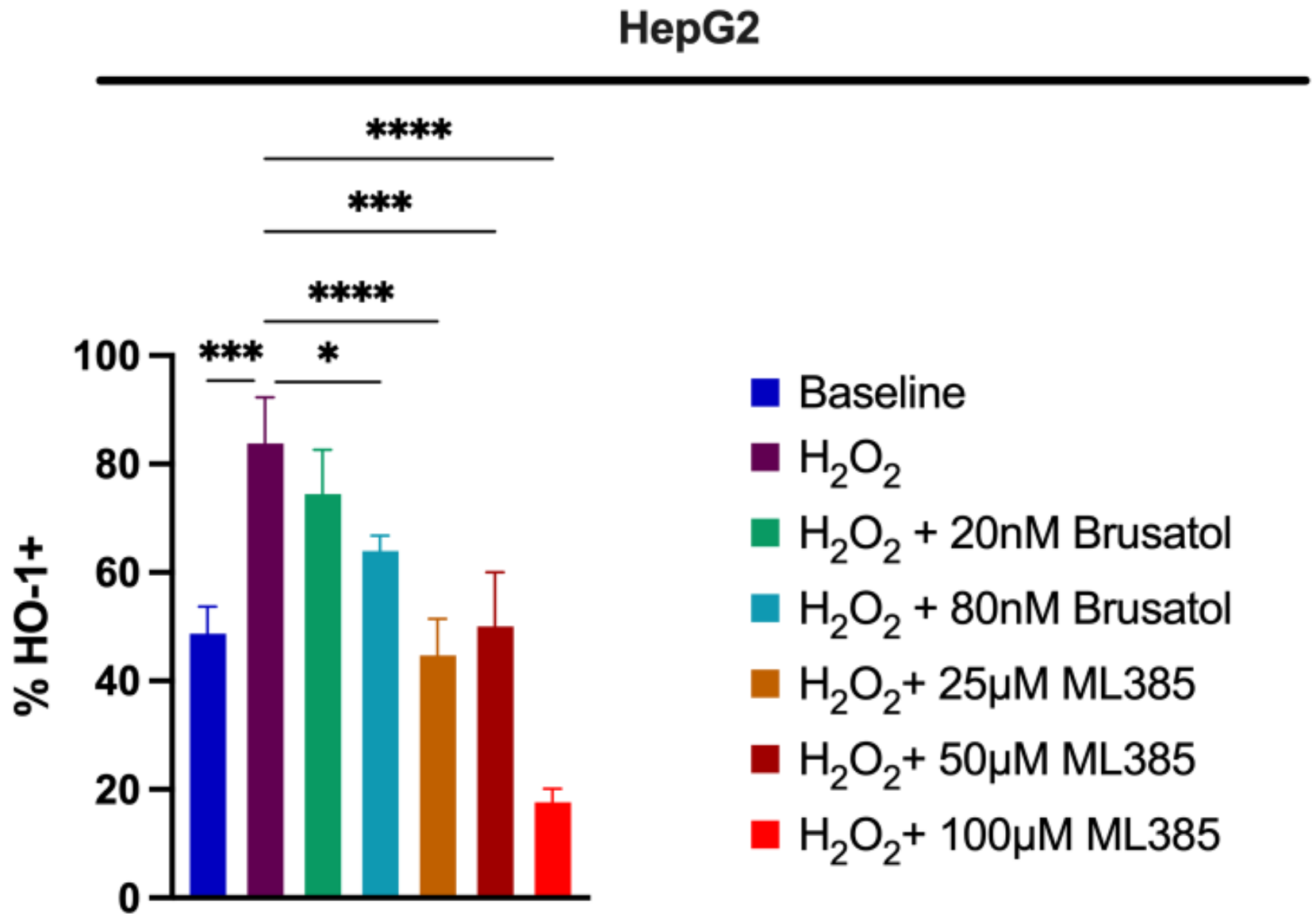

b

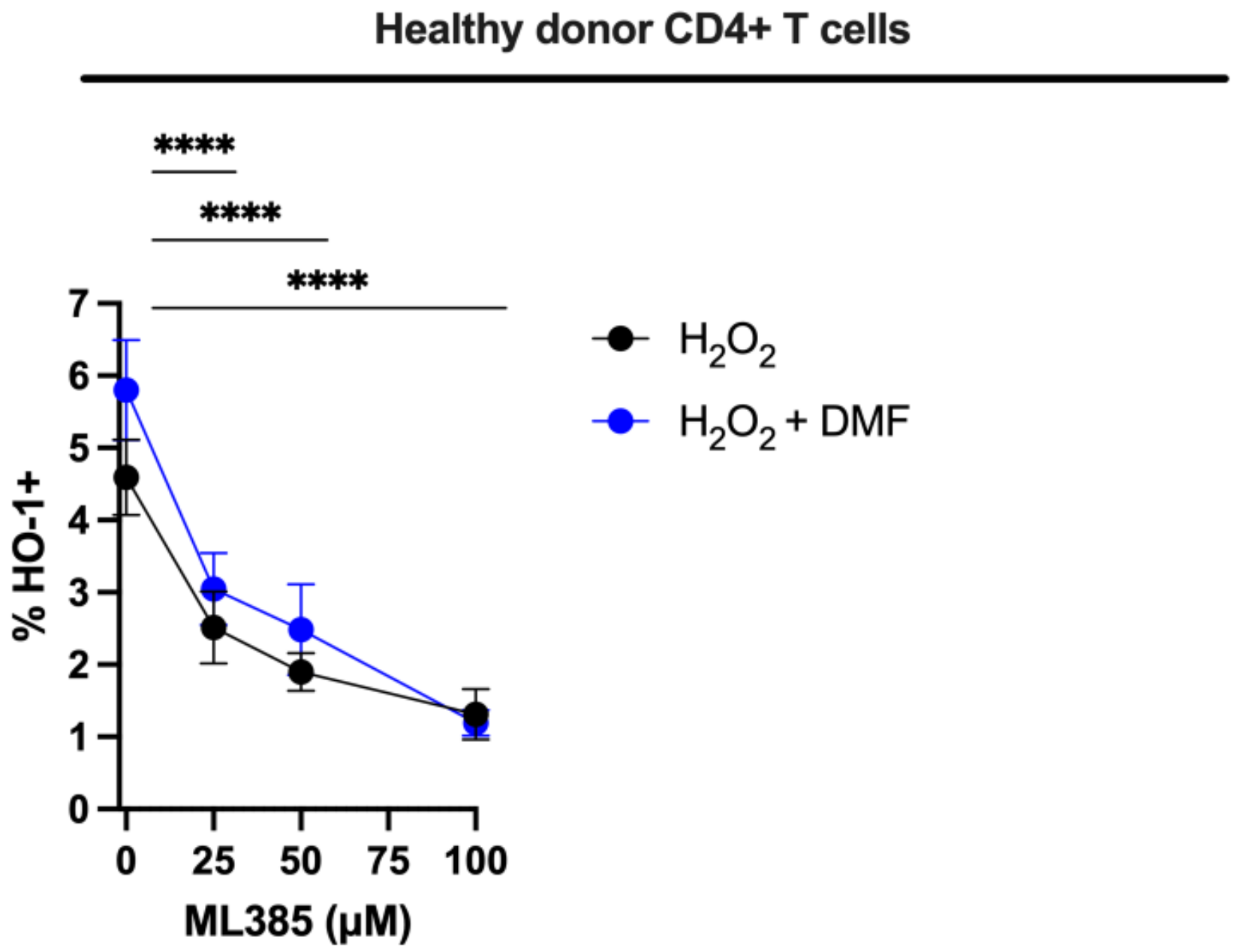

Supplementary Fig. 14. Pharmacological inhibition of Nrf2 using ML385. (a) Frequency of HO-1+ cells within HepG2 that were exposed for 16h to H_2O_2 and different concentration of two different Nrf2 inhibitors: Brusatol and ML385 (n=4 samples for each condition). **(b)** Frequency of HO-1+ cells within healthy donor CD4+ T cells that were exposed to H_2O_2±DMF for 45 minutes and then incubated with ML385 at different concentration for 16h (n=4 samples for each condition). P-value was calculated by one-way ANOVA with Tukey's multiple comparisons test. Only statistically significant P-values are displayed: *, P<0.05; **, P<0.01; ***, P<0.001; ****, P<0.0001

Supplementary Fig. 15

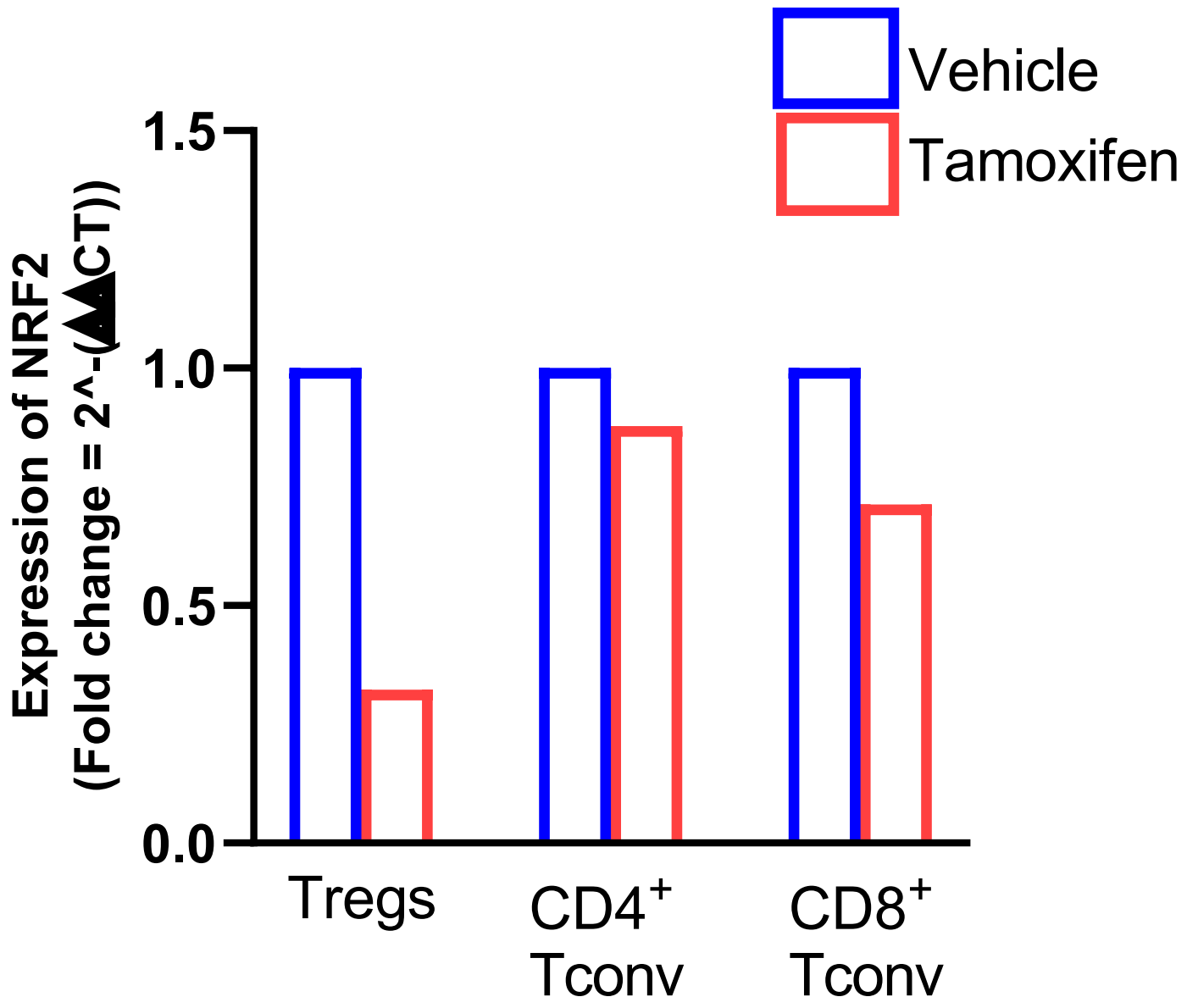

Supplementary Fig. 15. Fold change expression of *NFE2L2* (Nrf2) in Tregs, CD4+ and CD8+ Tconv cells in FoxP3^{GFPCre}ERT2Nrf2$^{fl/fl}$ mice treated with vehicle or tamoxifen.

Supplementary Fig. 16

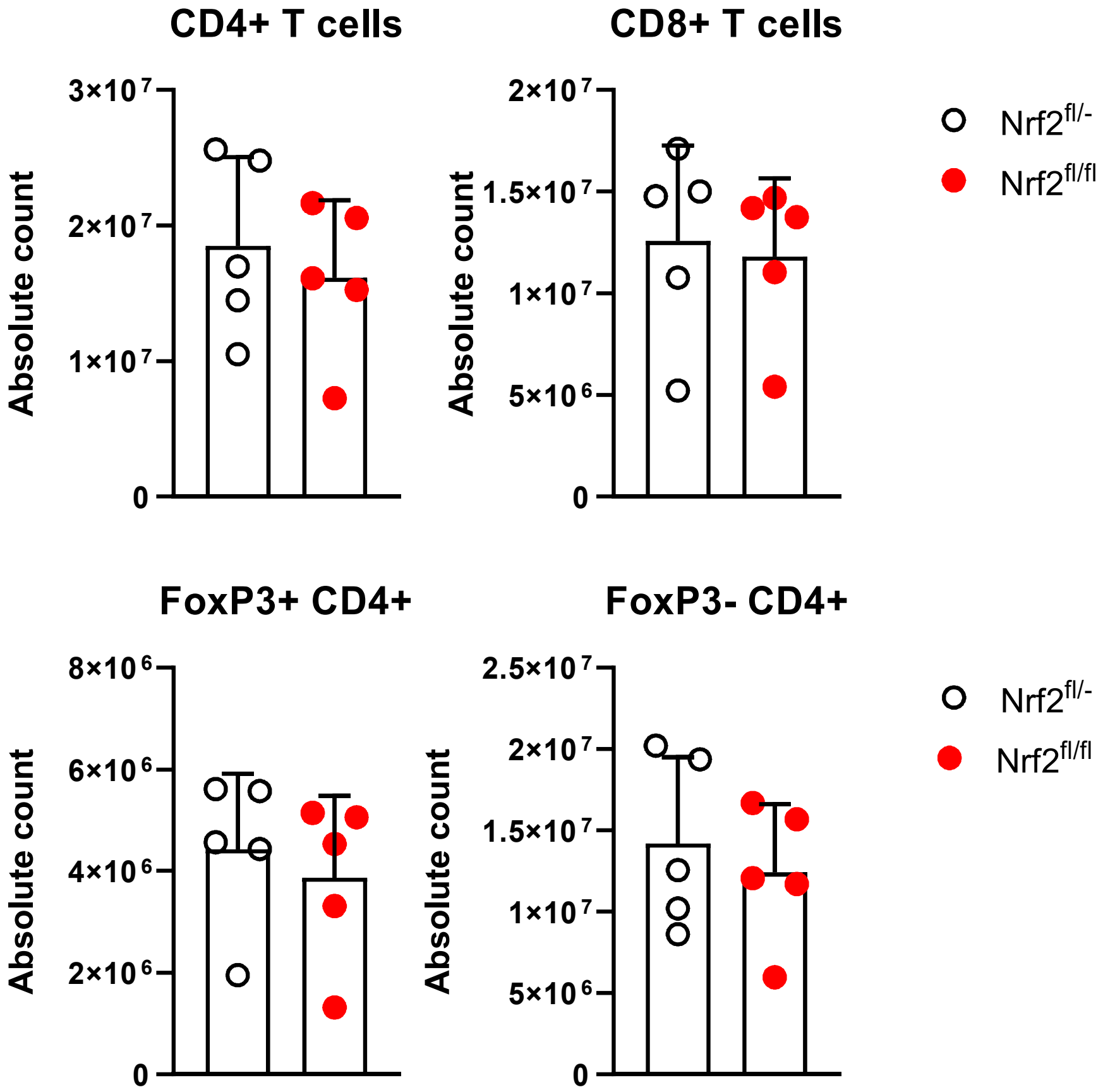

Supplementary Fig. 16. Splenic T cell numbers in the Treg-Nrf2 knock-down mouse model. Absolute count of T cell subsets identified in spleen from 5 control $Nrf2^{fl/-}$ ($FoxP3\text{-}Cre^{+/+}$ $Nrf2^{fl/-}$) and 5 Treg-Nrf2 knock-down, $Nrf2^{fl/fl}$, ($FoxP3\text{-}Cre^{+/+}$ $Nrf2^{fl/fl}$) mice after HCC inoculation. Mean +SD are shown. Data are presented as mean +SD. Statistical analysis using an unpaired t-test revealed no significant differences between the groups.

Supplementary Fig. 17

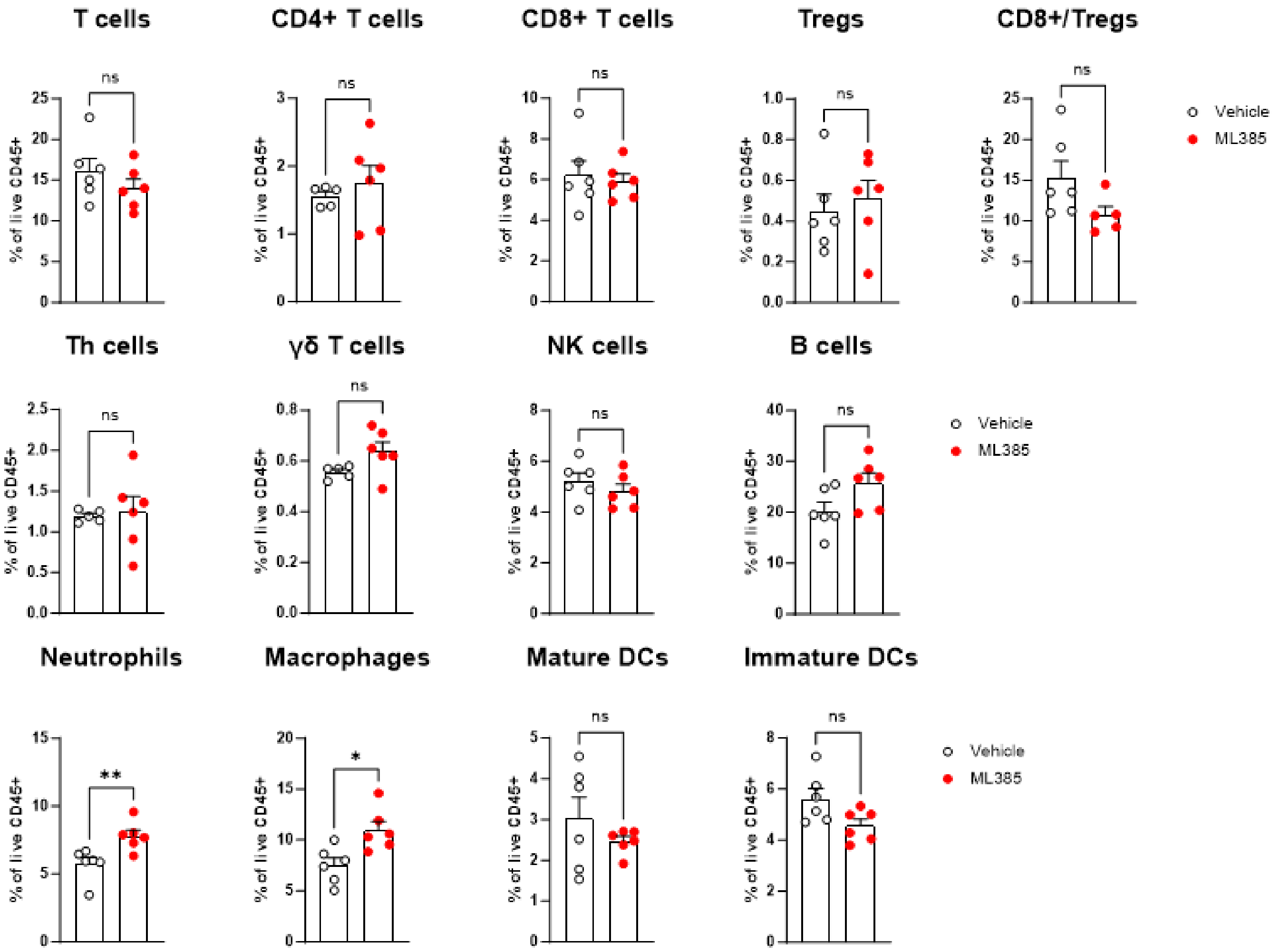

Supplementary Fig. 17. Immunophenotype analysis of non-tumoral liver tissue from the orthotopic HCC mouse model treated with ML385 or vehicle. Frequency of the different cell populations identified in the lymphoid and myeloid compartments from the CD45+ live cells. Mean +SD is shown. P-value was calculated by unpaired t test: *, P<0,05; **, P<0,01; ns, not significant.

Supplementary Fig. 18

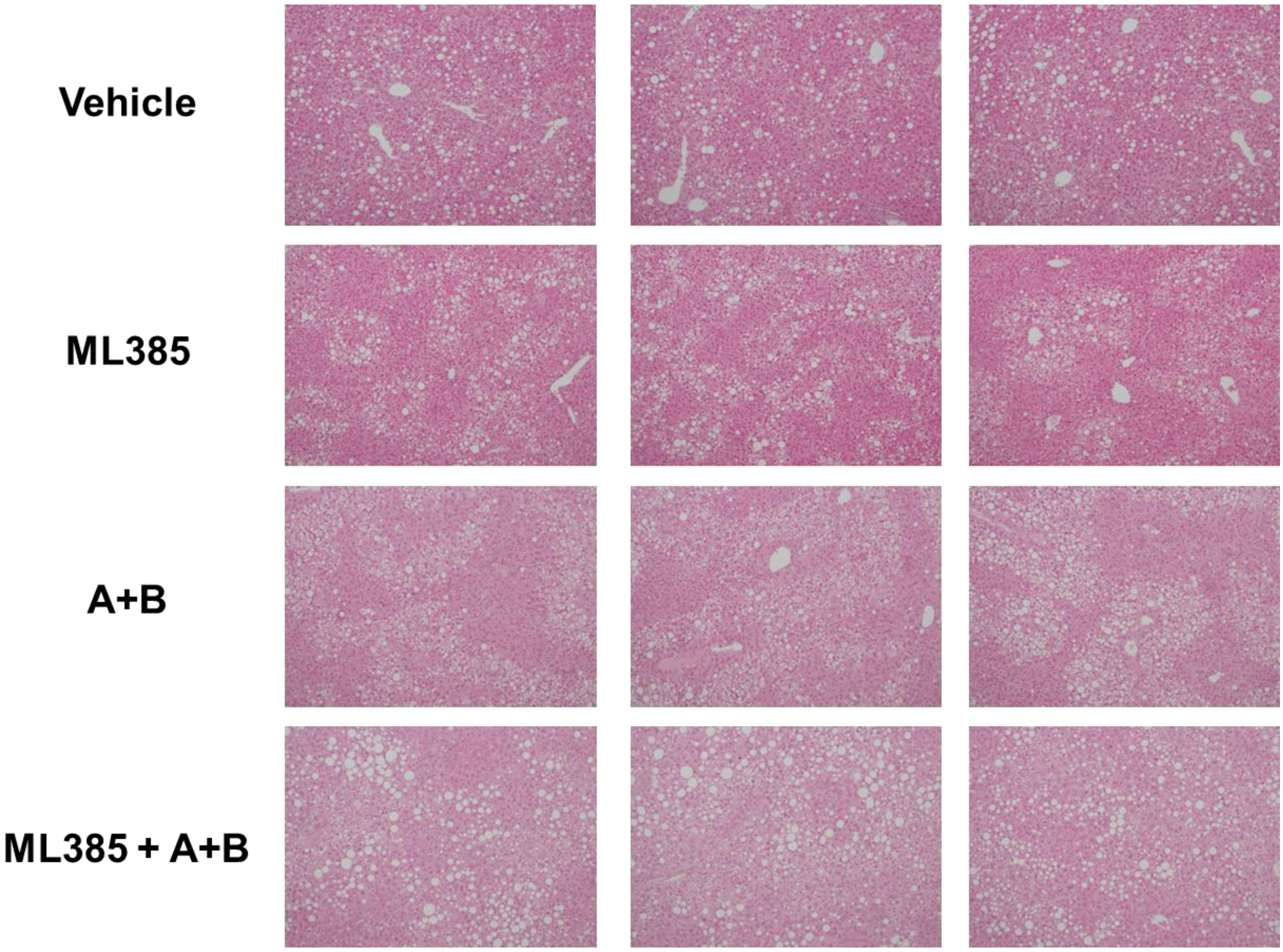

Supplementary Fig. 18. Histological analysis of liver sections from the orthotopic MASH-HCC murine model under different treatment conditions. Representative micrographs of liver tissue stained for histopathological evaluation from mice treated with vehicle, ML385, atezolizumab/ bevacizumab (A+B), or the combination ML385 + A+B. Each row corresponds to a treatment group, with three representative samples from one mouse shown per group. Histological features were assessed using the MASH scoring system, evaluating steatosis, inflammation, and ballooning degeneration. The images illustrate treatment-dependent differences in liver morphology, consistent with the quantified scores presented in Supplementary Table 15.

Supplementary Fig. 19

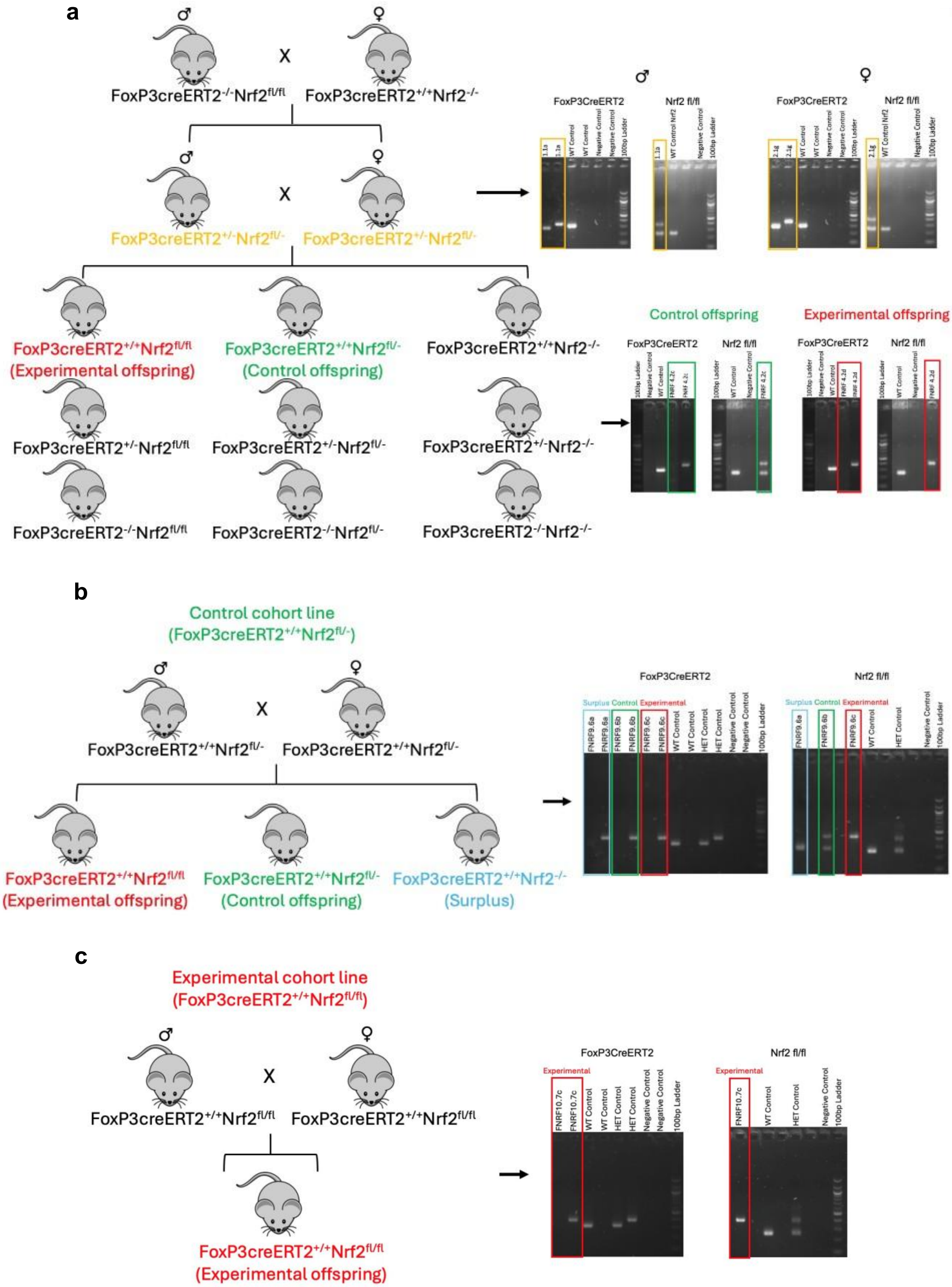

Supplementary Figure 19. Generation of FoxP3$^{\mathbf{GFPCre}}$ERT2Nrf2$^{\mathbf{fl/fl}}$ mice model. The schematic diagram describes the generation of FoxP3CreERT2$^{+/+}$Nrf2$^{fl/fl}$ mouse model. **(a)** FoxP3CreERT2$^{+/+}$Nrf2$^{-/-}$ mice (gift provided by Dr Ali/King's College London) were crossed with FoxP3CreERT2$^{-/-}$Nrf2$^{fl/fl}$ mouse (gift provided by Prof Shah/King's College London) to obtain the FoxP3CreERT2$^{+/-}$Nrf2$^{fl/-}$ intermediate offsprings that were heterozygous both in the FoxP3CreERT2 and Nrf2 alleles. The FoxP3CreERT2$^{+/-}$Nrf2$^{fl/-}$ mice were subsequently cross-bred to select experimental (FoxP3CreERT2$^{+/+}$Nrf2$^{fl/fl}$; red) and control (FoxP3CreERT2$^{+/+}$Nrf2$^{fl/-}$; green) animals respectively. **(b)** Scheme outlining the maintenance of control and **(c)** experimental animals. Representative genotyping results shown confirm the correct genotype of the animals. Parts of these were produced using vector by easyvectors.com on FreeImages (https://www.freeimages.com/vector/simple-cartoon-mouse-clip-art-5235124).

Supplementary Fig. 20

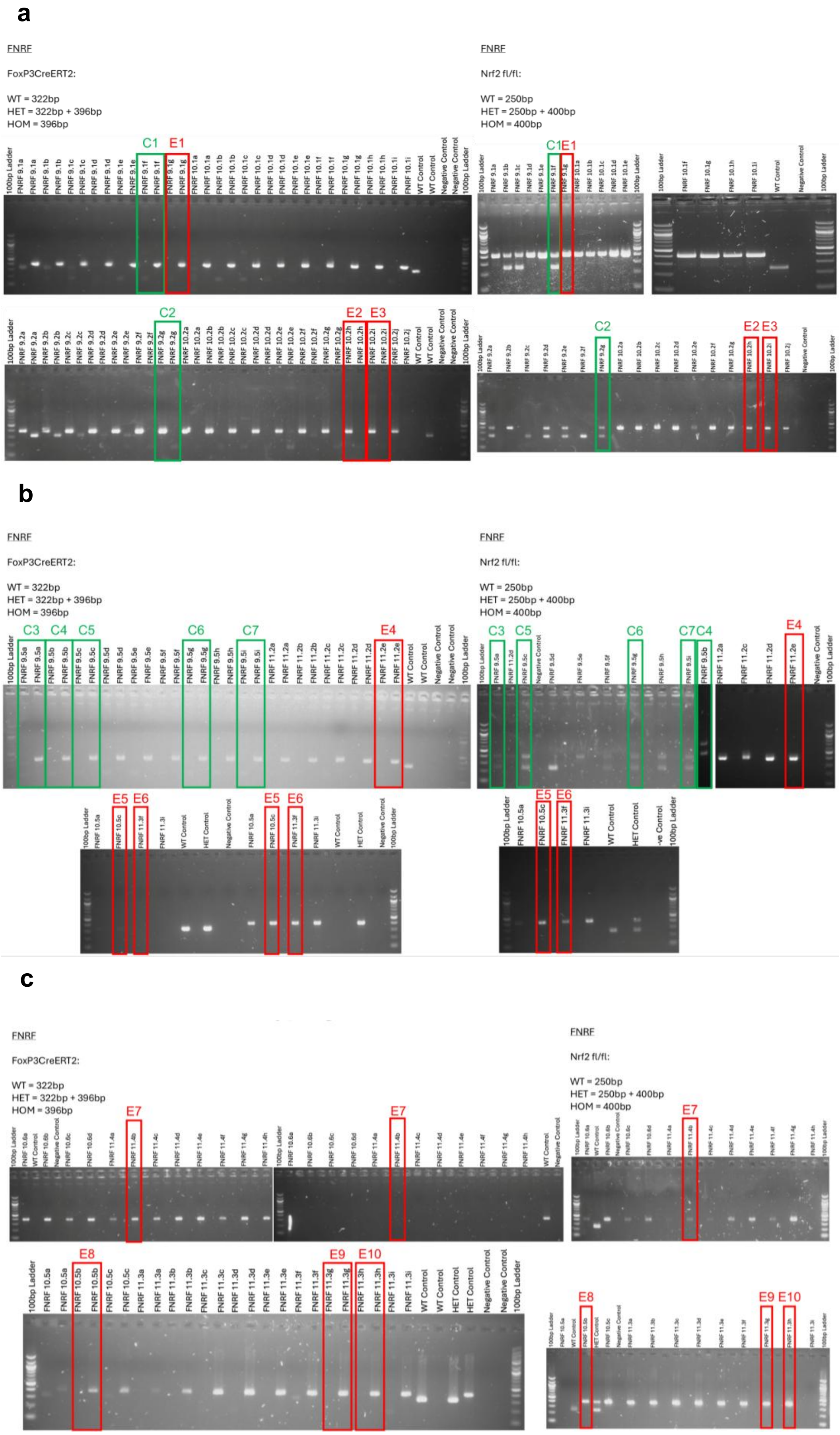

Supplementary Fig. 20. Full genotyping data of animals selected for experiments shown in Figures 7A-F. (a-c) Representative gel images of genotype analysis showing correct genotypes of the selected animals in the control and experimental cohorts. (Green frames) indicate control animals (FoxP3CreERT2$^{+/+}$Nrf2$^{fl/-}$) whereas (Red frames) indicate experimental animals (FoxP3CreERT2$^{+/+}$Nrf2$^{fl/fl}$).

Supplementary Fig. 21

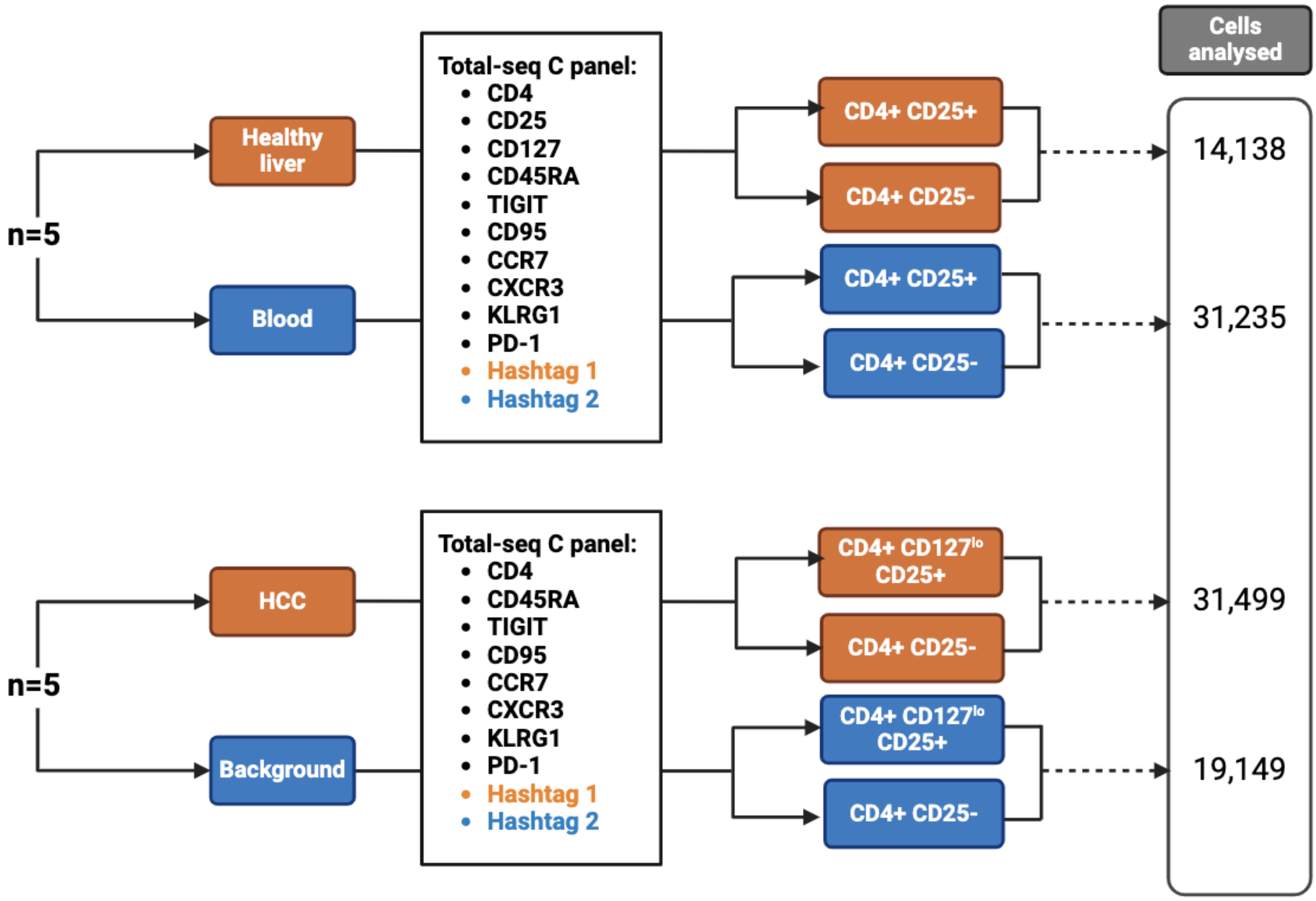

Supplementary Fig. 21. Schematic representation of the experimental setup for scRNAseq data acquisition. CITE-seq and cell sorting strategy to identify CD4+ Tregs and Tconv in healthy liver and paired blood (top panel, n=5 of each), and in HCC and paired background HCC tissues (bottom panel, n=5 of each). To allow sample multiplexing in each data set, cells from each specimen were labeled with a unique hashtag antibody. The reported number of analyzed cells represents the final count after data cleaning and filtering, utilized for subsequent analyses.

Supplementary Fig. 22

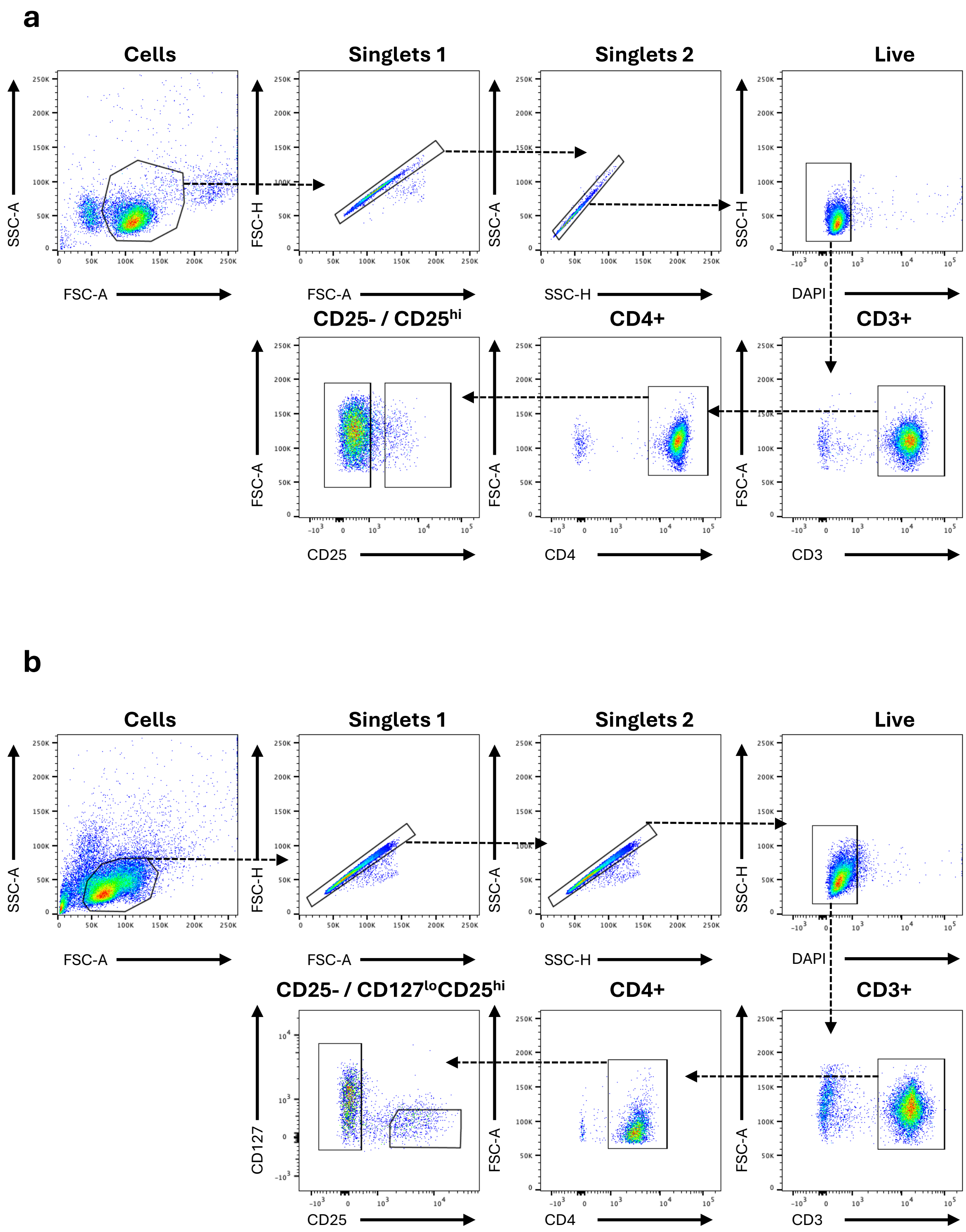

Supplementary Fig. 22. Gating strategy used for cell sorting prior to scRNA-seq analysis (Figures 2 and 3). Samples were first enriched for $CD4^+$ T cells using magnetic bead-based negative selection. The enriched $CD4^+$ population was then stained and gated by flow cytometry to isolate **(a)** CD4+ CD25- and $CD25^{hi}$ subsets; or **(b)** CD4+ CD25- and $CD127^{lo}$ $CD25^{hi}$ cells. Singlets and live cells were selected based on FSC/SSC and viability dye exclusion. CD127 marker could not be used in the first cell sorting strategy due to incompatibility with the CITE-seq CD127 antibody. The antibodies used were listed in Supplementary Table 17.

Supplementary Fig. 23

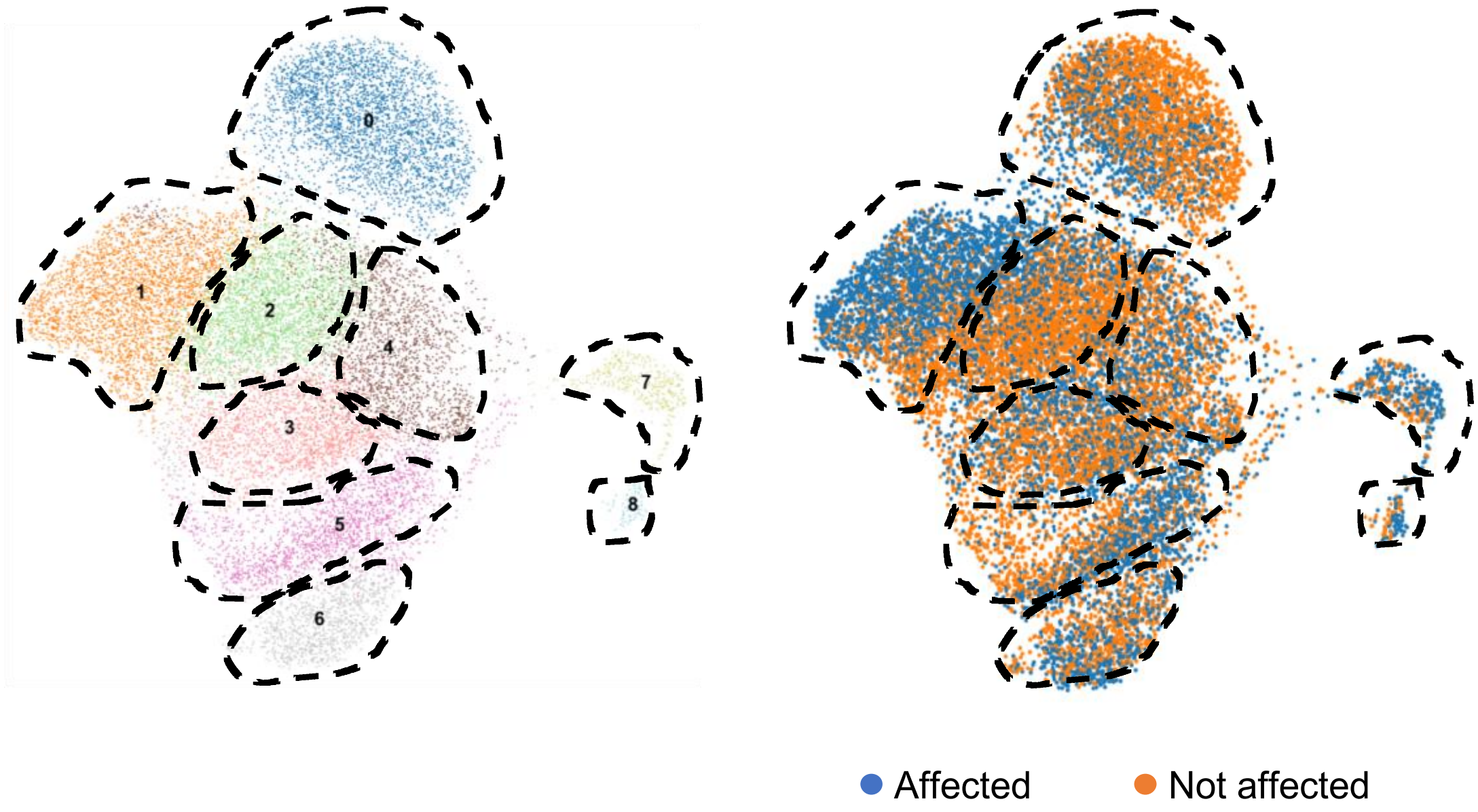

Supplementary Fig. 23. Impact of liver tissue digestion on cellular transcriptomes. UMAP of single cell transcriptomes of CD4+ T cells isolated from healthy liver (n=5). Cells are colour-coded according to subsets (left) or expression levels (right) of dissociation-induced genes. Cells exhibiting high expression levels are depicted in blue, denoting the "affected" category, while cells with low or no expression are depicted in orange, representing the "not affected" category.

Supplementary Fig. 24

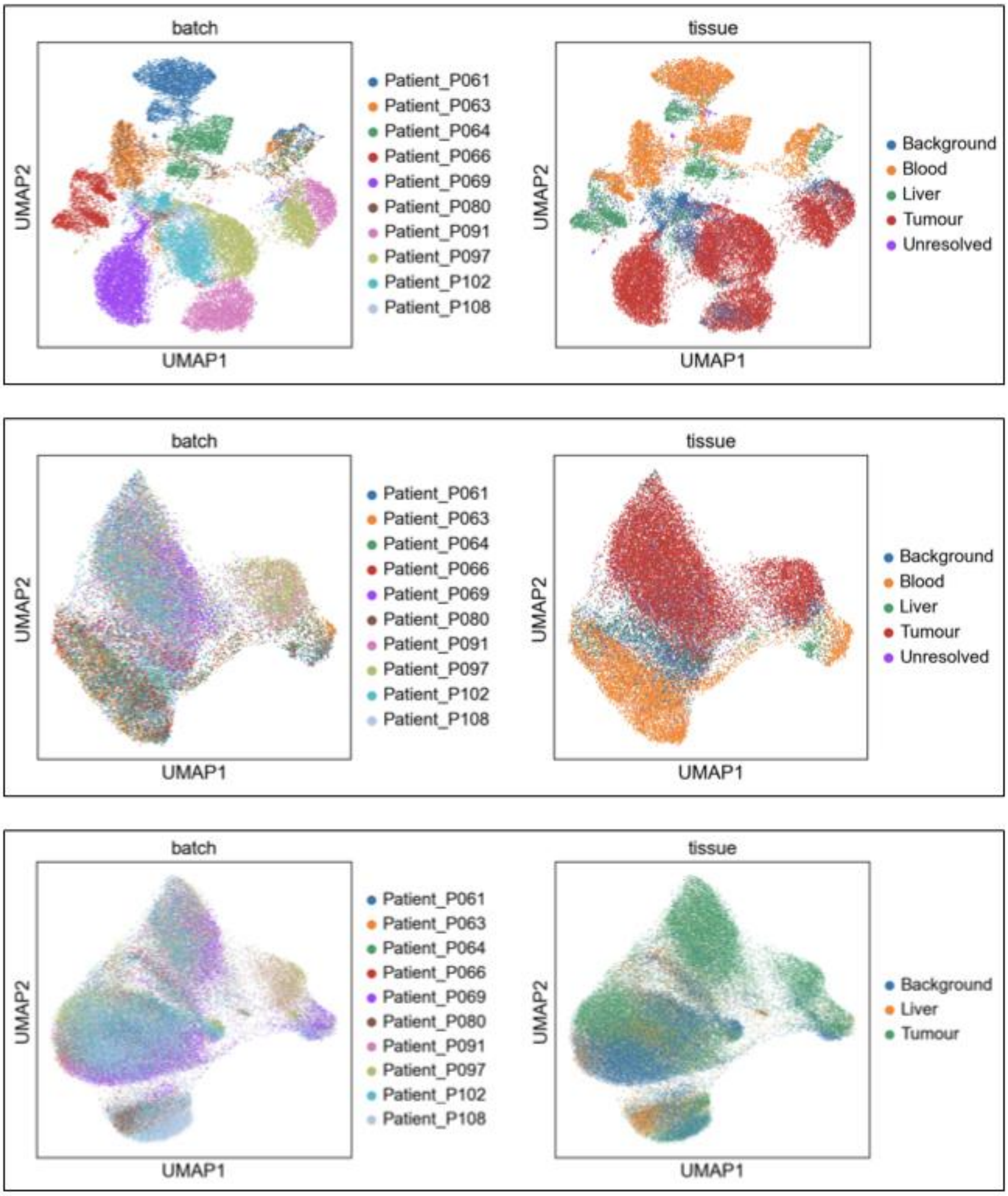

Supplementary Fig. 24. Integration analysis to correct batch effects in scRNA-seq data. Integration of scRNA-seq data from all specimens and patients, shown without batch effect correction (**top**) and with batch effect correction (**middle**). Panel (**bottom**) displays integration of liver tissue samples from different patients with batch correction applied, excluding paired PBMC samples from healthy tissues. UMAP plots on the left are colored by patient ID, with each dot representing a cell assigned to its respective patient. UMAP plots on the right are colored by specimen type: *background* (non-tumor liver tissue from HCC patients), *blood* (PBMCs paired with healthy liver), *liver* (healthy liver tissue), and *tumor* (HCC tissue).

Supplementary Fig. 25

a

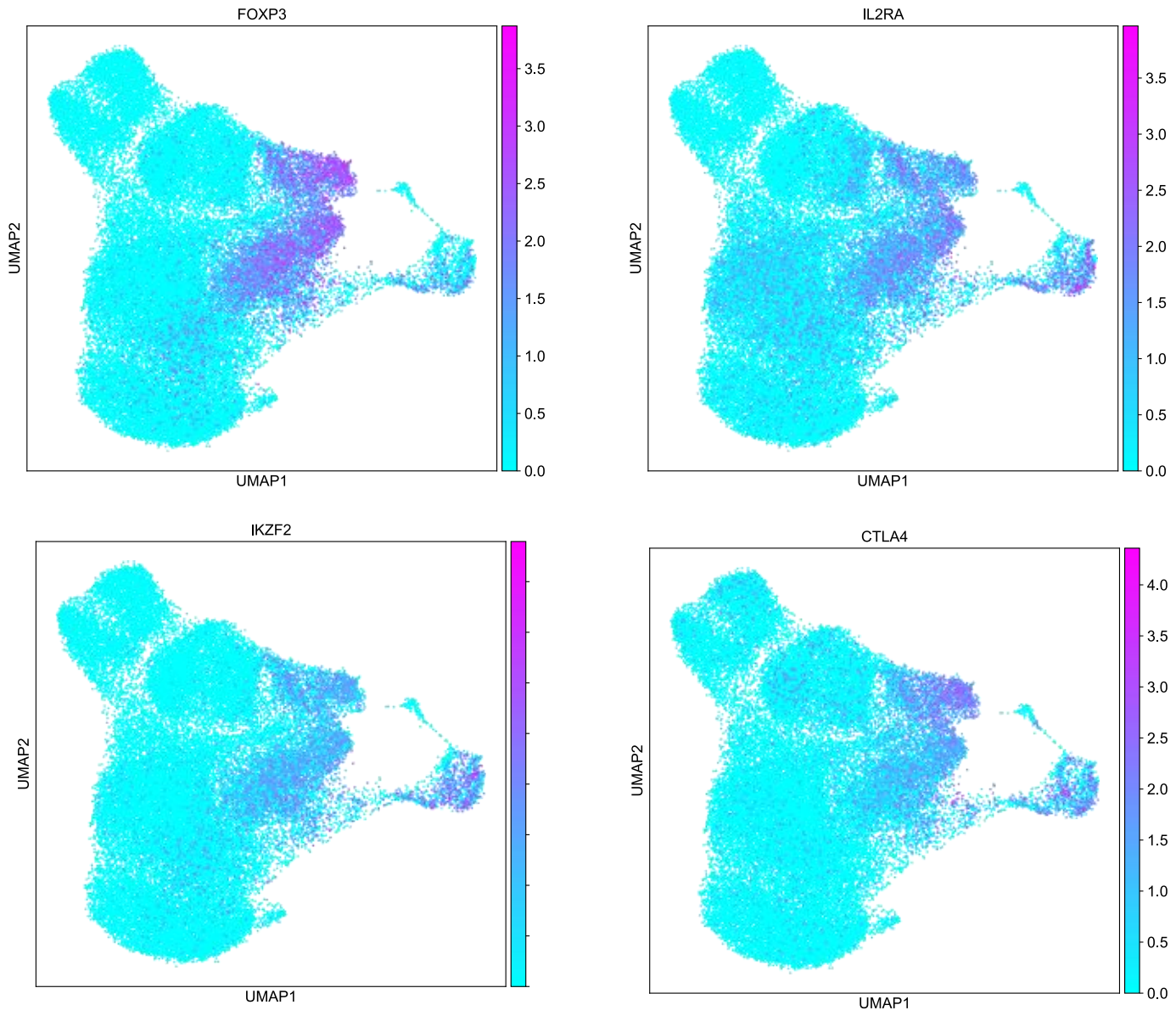

b

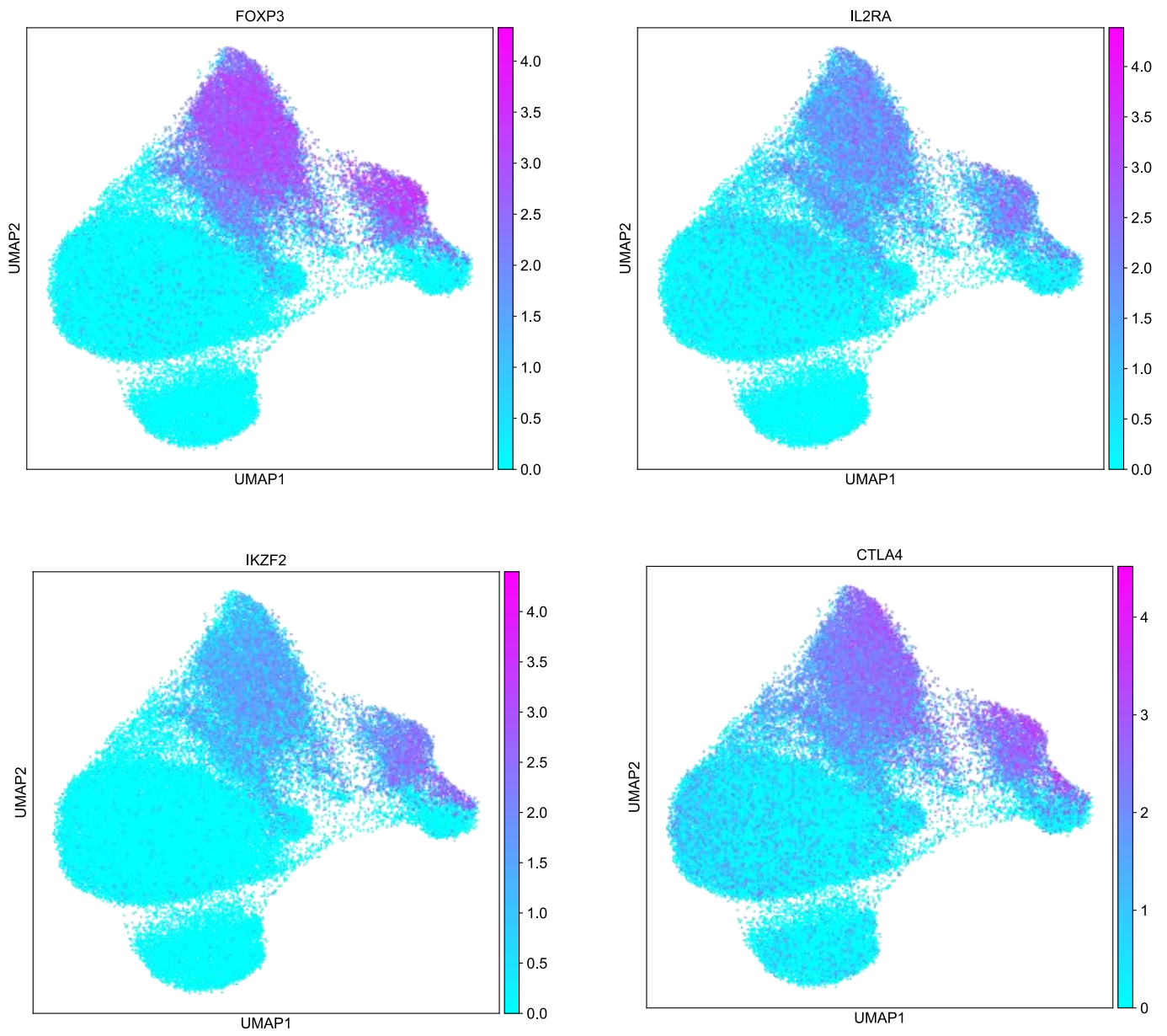

Supplementary Fig. 25. UMAP plots displaying the expression of canonical Treg markers across CD4+ clusters: *FOXP3*, *IL2RA* (CD25), *IKZF2* (HELIOS), and *CTLA4*. Each plot illustrates the expression of one gene, with color intensity indicating transcript abundance from high (purple) to low (blue) in **(a)** healthy liver and paired blood samples and in **(b)** liver compartments: healthy, HCC and paired background tissues.

Supplementary Figure 26

a

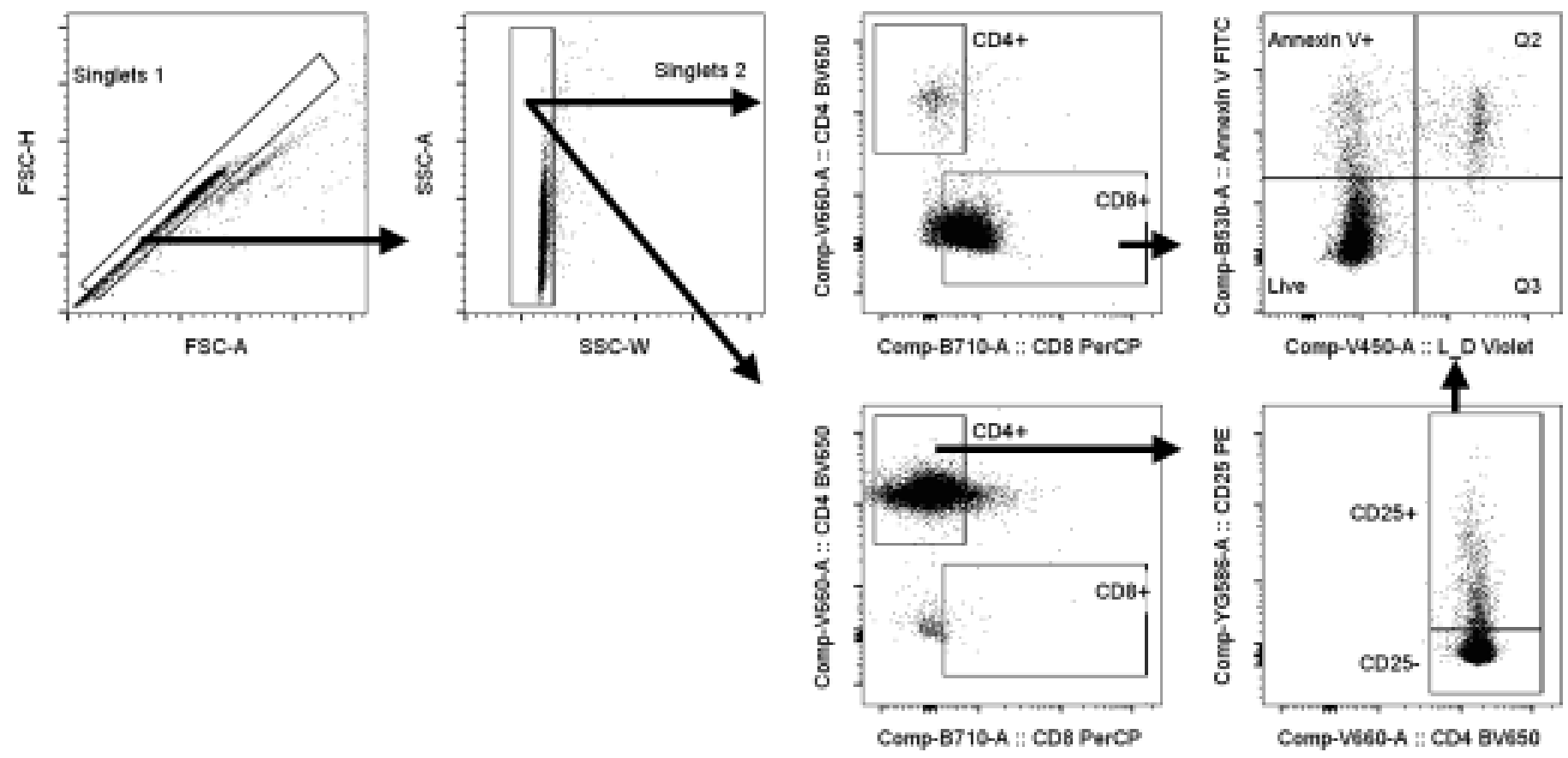

b

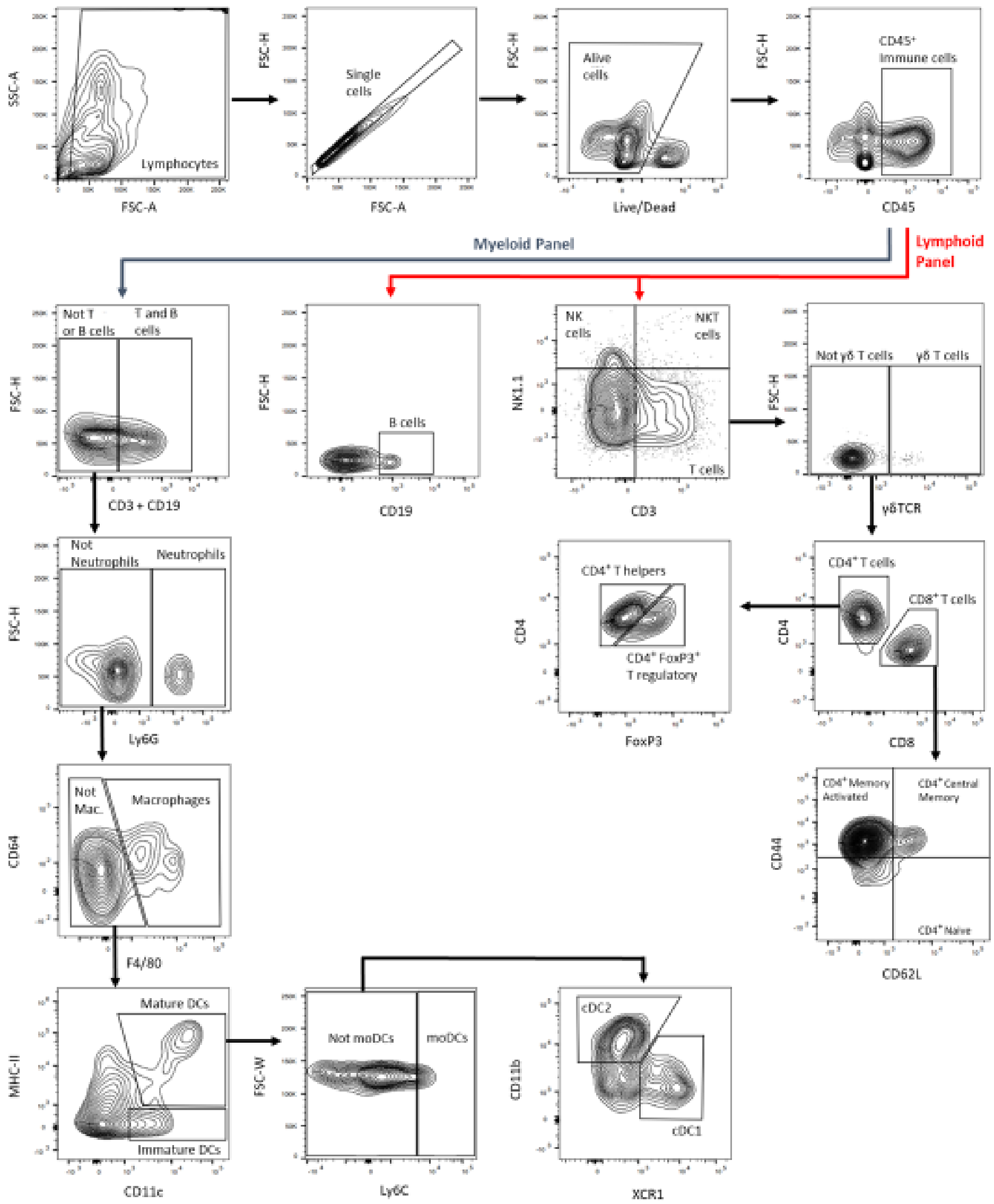

Supplementary Figure 26. Flow cytometry gating strategies used in the mouse experiments for the (a) identification and characterization of mouse Tregs and CD8+/CD4+ Tconv (**Fig. 6d-k** and **Supplementary Table 16**: "Apoptosis/viability murine T cells)"; and (**b**) immunophenotyping of mouse tumours (**Fig. 7i**) and **Supplementary Table 16**: "Immunophenotype orthotopic HCC mouse model".

Supplementary Tables

	Tconv					Treg				
**Cluster**	**ES**	**NES**	**Nominal p-value**	**FDR q-value**	**FWER p-value**	**ES**	**NES**	**Nominal p-value**	**FDR q-value**	**FWER p-value**
**0**	0,722	1,885	0,000	0,000	0,000	n/a	n/a	n/a	n/a	n/a
**1**	0,764	2,676	0,000	0,000	0,000	n/a	n/a	n/a	n/a	n/a
**2**	0,531	1,361	0,021	0,021	0,021	n/a	n/a	n/a	n/a	n/a
**3**	n/a	n/a	n/a	n/a	n/a	0,791	2,356	0,000	0,000	0,000
**4**	0,680	1,644	0,000	0,000	0,000	n/a	n/a	n/a	n/a	n/a
**5**	0,692	2,233	0,000	0,000	0,000	n/a	n/a	n/a	n/a	n/a
**6**	0,687	2,818	0,000	0,000	0,000	n/a	n/a	n/a	n/a	n/a
**7**	0,789	2,378	0,000	0,000	0,000	n/a	n/a	n/a	n/a	n/a
**8**	n/a	n/a	n/a	n/a	n/a	0,841	3,783	0,000	0,000	0,000
**9**	0,651	1,747	0,000	0,001	0,001	0,533	1,310	0,115	0,080	0,156
**10**	0,560	1,403	0,035	0,035	0,035	n/a	n/a	n/a	n/a	n/a
**11**	n/a	n/a	n/a	n/a	n/a	0,776	2,366	0,000	0,000	0,000
**12**	0,620	1,602	0,000	0,000	0,000	n/a	n/a	n/a	n/a	n/a
**13**	n/a	n/a	n/a	n/a	n/a	0,428	2,129	0,000	0,000	0,000
**14**	0,577	1,761	0,003	0,003	0,003	n/a	n/a	n/a	n/a	n/a
**15**	n/a	n/a	n/a	n/a	n/a	0,596	1,781	0,002	0,002	0,002
**16**	0,636	1,710	0,004	0,004	0,004	n/a	n/a	n/a	n/a	n/a

Supplementary Table 1. GSEA of reference Tconv- and Treg-gene signatures for the CD4+ clusters in healthy liver and paired PBMCs. FDR; false discovery rate; FWER, family-wise error rate; n/a, not applicable. GSEA used a weighted Kolmogorov–Smirnov–like statistic; nominal p-values were obtained by phenotype permutations, and multiple testing was controlled using FDR and FWER. Enrichment was evaluated in both directions (two-sided).

Cluster	Gene set	ES	NES	Nominal p-value	FDR q-value	FWER p-value
**0**	**Naïve**	0,660	1,840	0,000	0,000	0,000
**1**	**CM**	0,750	2,290	0,000	0,000	0,000
**2**	**EM**	0,510	1,230	0,014	0,019	0,018
**4**	**CM**	0,730	1,640	0,000	0,000	0,000
**5**	**Naïve**	0,760	2,220	0,000	0,000	0,000
**6**	**CM**	0,790	2,850	0,000	0,000	0,000
**7**	**EM**	0,630	1,770	0,001	0,001	0,001
**9**	**TEMRA**	0,500	1,440	0,000	0,012	0,023
**10**	**TEMRA**	0,530	1,460	0,000	0,023	0,023
**12**	**CM**	0,620	1,620	0,009	0,009	0,009
**14**	**EM**	0,750	2,300	0,000	0,000	0,000
**16**	**Naïve**	0,390	1,020	0,046	0,046	0,045

Supplementary Table 2. GSEA of reference naïve, terminally differentiated effector memory (TEMRA), central memory (CM) effector memory (EM) and T follicular helper (Tfh) signatures for the CD4+ Tconv clusters in healthy liver and paired blood. FDR; false discovery rate; FWER, family-wise error rate. GSEA used a weighted Kolmogorov–Smirnov–like statistic; nominal p-values were obtained by phenotype permutations, and multiple testing was controlled using FDR and FWER. Enrichment was evaluated in both directions (two-sided).

	Tconv				
**Cluster**	**ES**	**NES**	**Nominal p-value**	**FDR q-value**	**FWER p-value**
**0**	n/a	n/a	n/a	n/a	n/a
**1**	0,730	4,730	0,000	0,000	0,000
**2**	0,450	1,320	0,011	0,010	0,183
**3**	0,410	1,320	0,032	0,045	0,162
**4**	0,740	3,460	0,000	0,000	0,000
**5**	n/a	n/a	n/a	n/a	n/a
**6**	0,770	3,690	0,000	0,000	0,000
**7**	0,730	3,990	0,000	0,000	0,000
**8**	n/a	n/a	n/a	n/a	n/a
**9**	0,290	1,690	0,000	0,006	0,001
**10**	0,130	1,130	0,029	0,029	0,268
**11**	n/a	n/a	n/a	n/a	n/a
**12**	-0,300	-0,880	0,622	0,633	0,693
	**Treg**				
**Cluster**	**ES**	**NES**	**Nominal p-value**	**FDR q-value**	**FWER p-value**
**0**	0,800	3,690	0,000	0,000	0,000
**1**	n/a	n/a	n/a	n/a	n/a
**2**	-0,820	-2,380	0,000	0,000	0,000
**3**	-0,820	-2,690	0,000	0,000	0,000
**4**	n/a	n/a	n/a	n/a	n/a
**5**	0,880	4,230	0,000	0,000	0,000
**6**	n/a	n/a	n/a	n/a	n/a
**7**	n/a	n/a	n/a	n/a	n/a
**8**	0,610	3,470	0,000	0,000	0,000
**9**	n/a	n/a	n/a	n/a	n/a
**10**	n/a	n/a	n/a	n/a	n/a
**11**	0,580	2,550	0,000	0,000	0,000
**12**	-0,530	-1,430	0,103	0,178	0,126

Supplementary Table 3. GSEA of reference Tconv- and Treg-gene signatures for the CD4+ clusters in healthy liver, HCC and paired background tissues. FDR; false discovery rate; FWER, family-wise error rate; n/a, not applicable. GSEA used a weighted Kolmogorov–Smirnov–like statistic; nominal p-values were obtained by phenotype permutations, and multiple testing was controlled using FDR and FWER. Enrichment was evaluated in both directions (two-sided).

Cluster	Gene set	ES	NES	Nominal p-value	FDR q-value	FWER p-value
**1**	**CM**	0,570	2,940	0,000	0,000	0,000
**2**	**TEMRA**	0,770	3,670	0,000	0,000	0,000
**3**	**EM**	0,410	2,620	0,000	0,000	0,000
**4**	**EM**	0,470	1,710	0,013	0,013	0,015
**6**	**CM**	0,450	1,690	0,011	0,016	0,011
**6**	**EM**	0,450	1,620	0,011	0,014	0,022
**7**	**EM**	0,510	2,550	0,000	0,000	0,000
**9**	**CM**	0,570	2,020	0,000	0,000	0,000
**10**	**EM**	0,250	1,710	0,020	0,062	0,041

Supplementary Table 4. GSEA of reference naïve, terminally differentiated effector memory (TEMRA), central memory (CM) effector memory (EM) and T follicular helper (Tfh) signatures for the CD4+ Tconv clusters in healthy liver, HCC and paired background tissues. FDR; false discovery rate; FWER, family-wise error rate. GSEA used a weighted Kolmogorov–Smirnov–like statistic; nominal p-values were obtained by phenotype permutations, and multiple testing was controlled using FDR and FWER. Enrichment was evaluated in both directions (two-sided).

TNFRSF18	*GM2A*
TNFRSF4	*MYL6B*
SYNGR2	*STAC*
TNFRSF9	*FHL3*
CD82	*CCL20*
LAIR2	*SLCO4A1*
CTLA4	*LRRC32*
BATF	*C1orf112*
RANBP1	*CPA5*
SMS	*COL9A2*
PTP4A3	*TNS3*
ATOX1	*SGPP2*
EBI3	*RBBP8*
CD59	*TSPAN13*
TYMP	*CD80*
CTSC	*CLNK*
ID3	*DOT1L*
NME1	*SOX4*
PIM3	*TRIB1*
GLRX	*ZG16B*
MAGEH1	*CHRNA6*
GBP2	*CTTNBP2NL*
FABP5	
TBC1D4	
CD177	
OTUD5	
NAMPT	
TNIP3	
CUL9	
LMCD1	
NFKBID	
LAPTM4B	
SLC4A2	
GNG8	
CADM1	
TBC1D8	
TSPAN17	
AHSP	
CLEC7A	
IL2RA	
HMOX1	
PDGFA	

Supplementary Table 5. 64 gene-signature of Treg cluster B'.

ID	FoxP3CreERT2		Nrf2 fl/fl	
	Sample	Result	Sample	Result
C1	9.1f	HOM	9.1f	HET
C2	9.2g	HOM	9.2g	HET
C3	9.5a	HOM	9.5a	HET
C4	9.5b	HOM	9.5b	HET
C5	9.5c	HOM	9.5c	HET
C6	9.5i	HOM	9.5i	HET
C7	9.5g	HOM	9.5g	HET
	WT Control	WT	WT Control	WT
	HET Control	HET	HET Control	HET
	Negative Control	-ve	Negative Control	-ve
E1	9.1g	HOM	9.1g	HOM
E2	10.2h	HOM	10.2h	HOM
E3	10.2i	HOM	10.2i	HOM
E4	11.2e	HOM	11.2e	HOM
E5	10.5c	HOM	10.5c	HOM
E6	11.3f	HOM	11.3f	HOM
E7	11.4b	HOM	11.4b	HOM
E8	10.5b	HOM	10.5b	HOM
E9	11.3g	HOM	11.3g	HOM
E10	11.3h	HOM	11.3h	HOM
	WT Control	WT	WT Control	WT
	HET Control	HET	HET Control	HET
	Negative Control	-ve	Negative Control	-ve

Supplementary Table 6. Genotyping summary of the control (C) and experimental (E) mice of the tamoxifen-inducible Treg-specific Nrf2-knock-down colony. The summary table of the genotype results of the selected animals in the in vivo tumour growth comparison study. All animals in the control cohort were confirmed to be homozygous (HOM) in FoxP3CreERT2 allele but heterozygous (HET) in Nrf2 allele. On the other hand, all animals in the experimental cohort were confirmed to be homozygous (HOM) in both FoxP3CreERT2 allele and Nrf2 alleles.

Group	Mouse ID	Steatosis	Inflammation	Ballooning	Total
Vehicle	ER410	2	1	0	3
Vehicle	ER411	2	2	0	4
Vehicle	ER412	2	1	0	3
Vehicle	ER413	3	2	0	5
Vehicle	ER414	2	1	0	3
Vehicle	ER449	1	1	0	2
Vehicle	ER450	2	1	0	3
Vehicle	ER451	1	1	0	2
Vehicle	ER452	1	2	0	3
ML385	ER415	2	1	0	3
ML385	ER416	2	2	0	4
ML385	ER417	1	1	0	2
ML385	ER418	2	1	0	3
ML385	ER419	1	2	0	3
ML385	ER420	2	1	0	3
ML385	ER421	2	1	0	3
ML385	ER422	2	1	0	3
ML385	ER423	2	2	0	4
ML385	ER424	1	1	0	2
A+B	ER425	2	1	0	3
A+B	ER426	2	1	0	3
A+B	ER427	2	1	0	3
A+B	ER428	2	1	0	3
A+B	ER429	2	1	0	3
A+B	ER430	2	1	0	3
A+B	ER431	2	1	0	3
A+B	ER432	3	1	0	4
A+B	ER434	3	1	0	4
A+B	ER435	2	1	0	3
A+B	ER436	2	1	0	3
ML385 + A+B	ER437	1	1	0	2
ML385 + A+B	ER438	3	2	0	5
ML385 + A+B	ER439	2	1	0	3
ML385 + A+B	ER441	2	1	0	3
ML385 + A+B	ER442	2	2	0	4
ML385 + A+B	ER443	2	1	0	3
ML385 + A+B	ER444	3	2	0	5
ML385 + A+B	ER445	3	1	0	4
ML385 + A+B	ER446	1	1	0	2
ML385 + A+B	ER447	1	2	0	3
ML385 + A+B	ER448	2	1	0	3

Supplementary Table 7. Histopathological scoring of liver sections from the orthotopic MASH-HCC murine model under different treatment conditions. Individual scores for steatosis, inflammation, and hepatocellular ballooning were assessed for each mouse using the MASH scoring system. The total score represents the sum of these three parameters. Mice were grouped according to treatment: Vehicle, ML385, atezolizumab plus bevacizumab (A+B), and ML385 combined with A+B. These scores support the histological findings presented in Supplementary Fig. 18.

Antibody Panel	Target	Clone	Fluorophore	Manufacturer	Catalog number	ul/100ul
Apoptosis/viability human T cells	Annexin V	N/A	FITC	Biolegend	640906	5
	CD127	A019D5	BV711	Biolegend	351328	3
	CD25	2A3	APC	BD Bioscience	340907	5
	CD25	M-A251	APC	BD Bioscience	555434	5
	CD4	RPA-T4	BV605	Biolegend	300556	2,5
	CD45RA	HI100	BV650	Biolegend	304136	2
	CD8	HIT8a	PE/Cy7	Biolegend	300914	1
	FoxP3	259D	PE	Biolegend	320208	5
	Ki67	Ki-67	PerCP/Cy5.5	Biolegend	350520	5
	Viability	N/A	Live/Dead Fixable Aqua	Biolegend	423101/2	100 of 1:1000
Apoptosis/viability murine T cells	Annexin V	N/A	FITC	Biolegend	640906	5
	CD25	M-A251	PE	Biolegend	356103	0,625
	CD4	RM4-5	BV650	Biolegend	100555	1,5
	CD8	53-6.7	PerCP	Biolegend	100732	1,5
	Viability	N/A	Live/Dead Fixable Violet	ThermoFisher	L34955	100 of 1:1000
Human Treg count	CD127	A019D5	BV711	Biolegend	351328	3
	CD14	M5E2	PerCPCy5.5	Biolegend	301824	1
	CD19	HIB19	FITC	BD Bioscience	555412	1
	CD25	2A3	APC	BD Bioscience	340907	5
	CD25	M-A251	APC	BD Bioscience	555434	5
	CD3	UCHT1	APC/Cy7	Biolegend	300426	1
	CD4	RPA-T4	BV605	Biolegend	300556	2,5
	CD45RA	HI100	BV650	Biolegend	304136	2
	CD56	5.1H11	BV421	Biolegend	362551/2	2
	CD8	HIT8a	PE/Cy7	Biolegend	300914	1
	FoxP3	259D	PE	Biolegend	320208	5
	Viability	N/A	Live/Dead Fixable Aqua	Biolegend	423101/2	100 of 1:1000
MitoTrackers human T cells	CD127	A019D5	BV711	Biolegend	351328	3
	CD25	M-A251	PE	Biolegend	356103	0,625
	CD4	SK3	BUV395	BD Biosciences	563552	2
	CD8	HIT8a	PE-Cy7	Biolegend	300914	1
	MitoTracker DR	N/A	APC	Invitrogen	M22426	1.25 of 1:1000
	MitoTracker Green	N/A	FITC	Invitrogen	M7514	2 of 1:1000
	Viability	N/A	Live/Dead Fixable Violet	ThermoFisher	L34955	100 of 1:1000
MitoTrackers murine T cells	CD25	PC61	BV510	Biolegend	102042	5
	CD4	RM4-5	BV650	Biolegend	100555	1,5
	CD8	53-6.7	PerCP	Biolegend	100732	1,5
	MitoTracker DR	N/A	APC	Invitrogen	M22426	1.25 of 1:1000
	MitoTracker Green	N/A	FITC	Invitrogen	M7514	2 of 1:1000

	Viability	N/A	Live/Dead Fixable Violet	ThermoFisher	L34955	100 of 1:1000
Immunophenotype orthotopic HCC mouse model	Ly6C	HK1.4	PerCP-Cy5.5	Biolegend	128011	1:800
	CD45	30-F11	Alexa Fluor 700-Brilliant Violet 785	Biolegend	103128-103149	1:100
	CD64	X54-5/7.1	APC-Fire 750	Biolegend	139333	1:100
	CD11b	M1/70	Brilliant Violet 510	Biolegend	562950	1:100
	CD11c	N418	Brilliant Violet 605	Biolegend	117334	1:100
	XCR1	ZET	APC	Biolegend	148206	1:100
	Ly6G	1A8	Brilliant Violet 785	Biolegend	127645	1:100
	MHC-II	M5/114.15.2	BUV615	Biolegend	751570	1:100
	F4/80	T45-2342	BUV661	Biolegend	750643	1:100
	CD8	53-6.7	Alexa Fluor 700	Biolegend	100730	1:100
	CD4	RM4-5	FITC	Biolegend	100510	1:100
	FoxP3	MF-14	Brilliant Violet 421	Biolegend	126419	1:100
	NK1.1	PK136	Brilliant Violet 750	Biolegend	746876	1:100
	CD3	17A2	BUV805-BUV395	Biolegend	569192-740268	1:100
	gdTCR	GL3	BUV661	Biolegend	750410	1:100
	CD19	1D3	BUV805-BUV615	Biolegend	56287-751213	1:100
	Viability	N/A	BUV450-BUV515	Thermo Fisher	L34962	1:1000

Supplementary Table 8. Antibody panels employed in flow cytometry experiments.

All these panels were used with freshly isolated cells. N/A, not applicable.

Samples	Antibody Panel	Antibody	Clone	Manufacturer	Catalog number	ul/100ul
**Human healthy liver tissue and paired blood**	**CITE-seq staining**	Biotin anti-human CD25	BC96	Biolegend	302624	1
		TotalSeq-C0957 APC Streptavidin (CD25)	N/A	Biolegend	405285	1.25 of 1:10
		TotalSeq-C0045 anti-human CD4	SK3	Biolegend	344651	2.5 of 1:10
		TotalSeq-C0063 anti-human CD45RA	HI100	Biolegend	304163	2
		TotalSeq-C0088 anti-human CD279 (PD-1)	EH12.2H7	Biolegend	329963	1
		TotalSeq-C0089 anti-human TIGIT (VS3)	A15153G	Biolegend	372729	1
		TotalSeq-C0140 anti-human CD183 (CXCR3)	G025H7	Biolegend	353747	2
		TotalSeq-C0148 anti-human CD197 (CCR7)	G043H7	Biolegend	353251	2
		TotalSeq-C0151 anti-human CD152 (CTLA-4)	BNI3	Biolegend	369621	2
		TotalSeq-C0156 anti-human CD95 (Fas)	DX2	Biolegend	305651	1
		TotalSeq-C0390 anti-human CD127 (IL-7Rα)	A019D5	Biolegend	351356	1
		TotalSeq-C0250 anti-mouse/human KLRG1 (MAFA)	2F1/KLRG1	Biolegend	138433	1
		TotalSeq-C0251 anti-human Hashtag 1	LNH-94	Biolegend	394661	0,25
		TotalSeq-C0252 anti-human Hashtag 2	2M2	Biolegend	394663	0,25
	**Cell sorting**	APC/Cyanine7 anti-human CD3	UCHT1	Biolegend	300426	4
		Brilliant Violet 605 anti-human CD4	RPA-T4	Biolegend	300556	10
**Human tumour liver and paired background tissues**	**CITE-seq staining**	TotalSeq-C0045 anti-human CD4	SK3	Biolegend	344651	2.5 of 1:10
		TotalSeq-C0063 anti-human CD45RA	HI100	Biolegend	304163	2
		TotalSeq-C0088 anti-human CD279 (PD-1)	EH12.2H7	Biolegend	329963	1
		TotalSeq-C0089 anti-human TIGIT (VS3)	A15153G	Biolegend	372729	1
		TotalSeq-C0140 anti-human CD183 (CXCR3)	G025H7	Biolegend	353747	2
		TotalSeq-C0148 anti-human CD197 (CCR7)	G043H7	Biolegend	353251	2
		TotalSeq-C0151 anti-human CD152 (CTLA-4)	BNI3	Biolegend	369621	2
		TotalSeq-C0156 anti-human CD95 (Fas)	DX2	Biolegend	305651	1
		TotalSeq-C0250 anti-mouse/human KLRG1 (MAFA)	2F1/KLRG1	Biolegend	138433	1
		TotalSeq-C0251 anti-human Hashtag 1	LNH-94	Biolegend	394661	0,25
		TotalSeq-C0252 anti-human Hashtag 2	2M2	Biolegend	394663	0,25
	**Cell sorting**	APC/Cyanine7 anti-human CD3	UCHT1	Biolegend	300426	4

		Brilliant Violet 605 anti-human CD4	RPA-T4	Biolegend	300556	10
		APC anti-human CD25	2A3	BD Bioscience	340907	5
		APC anti-human CD25	M-A251	BD Bioscience	555434	5
		Brilliant Violent 711 anti-human CD127	A019D5	Biolegend	351328	3

Supplementary Table 9: Antibodies used for single-cell RNA seq experiments. N/A, not applicable.

Actg1
Ankrd1
Arid5a
Atf3
Atf4
Bhlhe40
Btg2
Ccrn4l
Cebpb
Csrnp1
Cxcl1
Cyr61
Dnajb4
Dusp1
Dusp8
Egr1
Egr2
Erf
Errfi1
Fos
Fosb
Fosl2
Gadd45a
Gadd45g
Gem
Hspa1a
Hspa1b
Hspb1
Ier2
Ier3
Ifrd1
Irf1
Itpkc
Jun
Junb
Jund
Klf2
Klf4
Klf6
Lmna
Maff
Mafk
Nfkbia
Nfkbiz
Nr4a1

Per1
Ppp1r15a
Rassf1
Rhob
Sat1
Serpine1
Slc38a2
Socs3
Srf
Stat3
Tagln2
Tiparp
Tnfaip3
Tppp3
Trib1
Ubc
Zfp36
Zfp36l1

Supplementary Table 10. Compilation of genes demonstrating upregulation across diverse cell populations post tissue dissociation at 37°C.

Target	Clone	Metal	Manufacturer	Catalog number	Concentration
ACAC	EPR4971	167Er	Abcam	ab231681	1/100
ASS1	EPR12398	159Tb	Abcam	ab231684	1/50
ATP5A	15H4C4	158Gd	Abcam	ab14748	1/50
CD127	A019D5	149Sm	Fluidigm	3149011B	1/50
CD25	2A3	169Tm	Fluidigm	3169003B	1/50
CD25	M-A251	169Tm	Biolegend	356102	1/50
CD3	UCHT1	141Pr	Fluidigm	3141019B	1/100
CD36	5-271	152Sm	Fluidigm	3152007B	1/100
CD4	RPA-T4	145Nd	Biolegend	300501	1/100
CD44	IM7	162Dy	Fluidigm	3162030B	1/200
CD45	HI30	89Y	Fluidigm	3089003B	1/200
CD45	HI30	106Cd	Fluidigm	3106001B	1/75
CD45	HI30	116Cd	Fluidigm	3116001B	1/50
CD45RA	HI100	143Nd	Fluidigm	3143006B	1/50
CD62L	DREG-56	153Eu	Fluidigm	3153004B	1/50
CD69	FN50	147Sm	Biolegend	310939	1/200
CD8a	RPA-T8	146Nd	Fluidigm	3146001B	1/50
CD98	MEM-108	150Nd	Biolegend	315602	1/50
CPT1A	8F6AE9	173Yb	Abcam	ab128568	1/200
CXCR6	K041E5	151Eu	Biolegend	356002	1/100
FABP5	311215	154Sm	Novus Biologicals	MAB3077	1/50
FoxP3	PCH101	171Yb	Invitrogen	14-4776-82	1/100
FoxP3	150D	171Yb	Biolegend	320002	1/100
FoxP3	259D	171Yb	Biolegend	320202	1/100
G6PD	EPR20668	164Dy	Abcam	ab231828	1/50
GLUT1	EPR3915	168Er	Abcam	ab196357	1/50
Helios	22F6	175Lu	Biolegend	137202	1/100
HK1	EPR10134(B)	170Er	Abcam	ab233837	1/100
IDH2	EPR7577	165Ho	Abcam	ab230796	1/200
Ki67	Ki-67	176Yb	Biolegend	350502	1/100
LDHA	EP1566Y	166Er	Abcam	ab219591	1/50
MCT1	P14612	172Yb	Thermo Fisher	MA5-18288	1/50
NRF2p	EPI809Y	142Nd	Abcam	ab180844	1/100
OPA1	EPR11057(B)	160Gd	Abcam	ab240143	1/100

Supplementary Table 11. Antibodies used for CyTOF experiments.

Target	Clone	Fluorophore	Manufacturer	Catalog number
CD4	RPA-T4	APC-Cy7	BD	557871
CD14	HCD14	PerCP-Cy5.5	BioLegend	325622
CD19	HIB19	Pacific blue	BioLegend	302232
CD25	2A3	PE	BD	341011
CD25	M-A251	PE	BD	555432
CD127	HIL-7R-M21	Alexa Fluor 647	BD	558598

Supplementary Table 12. Antibodies used for cell sorting for the in vitro Treg micro-suppression assay.
