# Supplementary material for: Nrf2-mediated metabolic reprogramming drives regulatory T cell accumulation in hepatocellular carcinoma
Source: Nat Commun. 2026 Jul 13;17:8639. doi: 10.1038/s41467-026-73485-3 (PMC13487165; doi:10.1038/s41467-026-73485-3)
Supplement: Supplementary file 2 [file 41467_2026_73485_MOESM2_ESM.pdf]

Description of Additional Supplementary Files

Supplementary Data 1. Gene signatures used to identify Treg and Tconv cells.

Supplementary Data 2. Gene signatures used to identify the different Tconv subsets. TEMRA, terminally differentiated effector memory; EM, effector memory; CM, central memory; Tfh, T follicular helper.

Supplementary Data 3. Top 100 most frequent TCR clonotypes identified within distinct CD4+ T cell clusters, indicating the clone designation and the corresponding cell counts expressing each TCR clonotype within each cluster.

Supplementary Data 4. Top 100 DEGs ranked by P-value from the pseudo-bulk analysis comparing liver Tregs and blood Tregs. Non-parametric Wilcoxon rank sum tests were set to evaluate the significance of each individual DEG.

Supplementary Data 5. Top 100 DEGs, ranked by adjusted P-value, between the Treg clusters in liver and blood, assessed by comparing one cluster vs the others. pvals_adj; adjusted P-value. Non-parametric Wilcoxon rank sum tests were set to evaluate the significance of each individual DEG.

Supplementary Data 6. Top 100 DEGs, ranked by adjusted P-value, between the Treg clusters in healthy liver, HCC and background tissues, assessed by comparing one cluster vs the others. pvals_adj, adjusted P-value. Non-parametric Wilcoxon rank sum tests were set to evaluate the significance of each individual DEG.

Supplementary Data 7. Statistical analysis of the metabolic markers' expression within the different HCC Treg subpopulations. Adjusted p-value was calculated by Two-way Anova with multiple Tukey's comparisons test.

Supplementary Data 8. Demographic and clinical characteristics of study participants.

Supplementary Data 9. Top 100 DEGs, ranked by adjusted p-value, between the CD4+ T clusters in liver, assessed by comparing one cluster vs the others. pvals_adj; adjusted P-value. Non-parametric Wilcoxon rank sum tests were set to evaluate the significance of each individual DEG.
