# Supplementary material for: Nrf2-mediated metabolic reprogramming drives regulatory T cell accumulation in hepatocellular carcinoma
Source: Nat Commun. 2026 Jul 13;17:8639. doi: 10.1038/s41467-026-73485-3 (PMC13487165; doi:10.1038/s41467-026-73485-3)
Supplement: Supplementary file 3 [file 41467_2026_73485_MOESM4_ESM.pdf]

nature portfolio

Corresponding author(s): Niloufar Safinia

Last updated by author(s): Jan 17, 2026

Reporting Summary

Nature Portfolio wishes to improve the reproducibility of the work that we publish. This form provides structure for consistency and transparency in reporting. For further information on Nature Portfolio policies, see our Editorial Policies and the Editorial Policy Checklist.

Statistics

For all statistical analyses, confirm that the following items are present in the figure legend, table legend, main text, or Methods section.

n/a	Confirmed	
☐	☒	The exact sample size (*n*) for each experimental group/condition, given as a discrete number and unit of measurement
☐	☒	A statement on whether measurements were taken from distinct samples or whether the same sample was measured repeatedly
☐	☒	The statistical test(s) used AND whether they are one- or two-sided<br>*Only common tests should be described solely by name; describe more complex techniques in the Methods section.*
☐	☒	A description of all covariates tested
☐	☒	A description of any assumptions or corrections, such as tests of normality and adjustment for multiple comparisons
☐	☒	A full description of the statistical parameters including central tendency (e.g. means) or other basic estimates (e.g. regression coefficient) AND variation (e.g. standard deviation) or associated estimates of uncertainty (e.g. confidence intervals)
☐	☒	For null hypothesis testing, the test statistic (e.g. *F*, *t*, *r*) with confidence intervals, effect sizes, degrees of freedom and *P* value noted<br>*Give P values as exact values whenever suitable.*
☒	☐	For Bayesian analysis, information on the choice of priors and Markov chain Monte Carlo settings
☒	☐	For hierarchical and complex designs, identification of the appropriate level for tests and full reporting of outcomes
☐	☒	Estimates of effect sizes (e.g. Cohen's *d*, Pearson's *r*), indicating how they were calculated

Our web collection on statistics for biologists contains articles on many of the points above.

Software and code

Policy information about availability of computer code

Data collection	BD FACSDiva software (version 9) was used to collect all flow cytometry data on BD LSR Fortessa flow cytometer (BD Biosciences), except data from the orthotopic mouse HCC model that was collected on a BD Symphony A5 flow cytometer (BD Biosciences).<br>CyTOF software (version 7.0.8493.0) was used to collect mass cytometer data on Helios mass cytometer (Fluidigm).
Data analysis	FlowJo (version 10.7.1)<br>Cell Ranger (version 7.2)<br>Python (version 3.9.13)<br>Scanpy (version 1.9.6)<br>GSEA (version 4.3.2)<br>GSVA (version 1.0.6)<br>GSEApy (version 1.1.0)<br>Scirpy (version 0.13.1)<br>Seurat R (version 4.0)<br>CyTOF software (version 7.0.8493.0)<br>Cytobank (version 10.3)<br>Graphpad Prism (version 10.1.1)

For manuscripts utilizing custom algorithms or software that are central to the research but not yet described in published literature, software must be made available to editors and reviewers. We strongly encourage code deposition in a community repository (e.g. GitHub). See the Nature Portfolio guidelines for submitting code & software for further information.

Data

Policy information about availability of data

All manuscripts must include a data availability statement. This statement should provide the following information, where applicable:

- Accession codes, unique identifiers, or web links for publicly available datasets
- A description of any restrictions on data availability
- For clinical datasets or third party data, please ensure that the statement adheres to our policy

All data supporting the findings of this study are available within the paper and its supplementary information. Multi-omics single-cell data generated in this study has been deposited in the NCBI Gene Expression Omnibus (GEO) under accession number GSE320155 (https://www.ncbi.nlm.nih.gov/geo/query/acc.cgi?acc=GSE320155). Processed and annotated scanpy objects can be downloaded from a Zenodo repository (https://doi.org/10.5281/zenodo.15471231).

Research involving human participants, their data, or biological material

Policy information about studies with human participants or human data. See also policy information about sex, gender (identity/presentation), and sexual orientation and race, ethnicity and racism.

Reporting on sex and gender	Both female and male participants were recruited (17 female and 36 male). Detailed information regarding sex and gender is provided in Supplementary Table 13. Sex was not considered during the study design. Due to the limited number of female participants per experimental group, sex- and gender-based analyses were not performed.
Reporting on race, ethnicity, or other socially relevant groupings	Race, ethnicity and other socially relevant demographic variables were not collected as part of participant recruitment or analysis in this study. As such, these characteristics were not considered in the design, conduct, or interpretation of the research.
Population characteristics	The target population were patients undergoing surgical liver resections for either HCC, metastasis, or benign liver lesions at King's College Hospital (London, UK). Patients with abnormal livest tests and/or recent (<2 months) administration of chemotherapeutic agents were excluded.
Recruitment	Demographic and clinical characteristics (sex, age, diagnosis, treatment history and co-morbidities) of study participants were collected through the review of electronic medical record and are detailed in Supplementary Table 13.
Ethics oversight	All samples were obtained under the remit of the King's College Hospital Liver Tissue Bank (UK Research Ethics Committee approval reference: 23/LO/0708; IRAS ID: 332608)

Note that full information on the approval of the study protocol must also be provided in the manuscript.

Field-specific reporting

Please select the one below that is the best fit for your research. If you are not sure, read the appropriate sections before making your selection.

☒ Life sciences ☐ Behavioural & social sciences ☐ Ecological, evolutionary & environmental sciences

For a reference copy of the document with all sections, see nature.com/documents/nr-reporting-summary-flat.pdf

Life sciences study design

All studies must disclose on these points even when the disclosure is negative.

Sample size	The patient sample size was not determined using any statistical method, as they were collected based on their availability.
Data exclusions	No data were excluded.
Replication	All flow and mass cytometry, scRNA-seq, in vitro and in vivo studies were conducted using biological replicates. qPCR assays were carried out with two technical replicates, while human Treg microsuppression assays were performed with a minimum of three technical replicates. Due to limited cell numbers, technical replicates from patient samples were not included. To prevent any technical bias, CyTOF assays included PBMCs from the same healthy donor in each staining batch. All attempts at replication were successful.
Randomization	Randomization was unnecessary since samples were selected based on clinical eligibility criteria. For the in vitro studies, each biological sample included appropriate treatment controls.
Blinding	Blinding was not required because clinical samples were obtained as available from surgical resections and processed using standardized protocols. No experimental steps relied on subjective evaluation that could be influenced by knowledge of sample identity.

Reporting for specific materials, systems and methods

We require information from authors about some types of materials, experimental systems and methods used in many studies. Here, indicate whether each material, system or method listed is relevant to your study. If you are not sure if a list item applies to your research, read the appropriate section before selecting a response.

Materials & experimental systems

n/a	Involved in the study
☐	☒ Antibodies
☐	☒ Eukaryotic cell lines
☐	☐ Palaeontology and archaeology
☐	☒ Animals and other organisms
☐	☒ Clinical data
☐	☐ Dual use research of concern
☐	☐ Plants

Methods

n/a	Involved in the study
☐	☐ ChIP-seq
☐	☒ Flow cytometry
☐	☐ MRI-based neuroimaging

Antibodies

Antibodies used

Details of all antibodies used in this study can be found in Supplementary Tables 15, 16, 19, 20, and in the Methods section, as well as listed below:

Human apoptosis/viability/Treg count/mitochondria/cell sorting:
-FITC Annexin V (Biolegend, clone N/A, cat. 640906, dilution 5:100)
-Anti-human -BV711 anti-human CD127 (Biolegend, clone A019D5, cat. 351328, dilution 3:100)
-APC anti-human CD25 (BD Bioscience, clone 2A3, cat. 340907, dilution 5:100)
-APC anti-human CD25 (BD Bioscience, clone M-A251, cat. 555434, dilution 5:100)
-PE anti-human CD25 (Biolegend, clone M-A251, cat. 356103, dilution 0.625:100)
-BV605 anti-human CD4 (Biolegend, clone RPA-T4, cat. 300556, dilution 2.5:100)
-BUV395 anti-human CD4 (BD Biosciences, clone SK3, cat. 564552, dilution 2:100)
-BV650 anti-human CD45RA (Biolegend, clone HI100, cat. 304136, dilution 2:100)
-PE/Cy7 anti-human CD8 (Biolegend, clone HIT8a, cat. 300914, dilution 1:100)
-PE anti-human FoxP3 (Biolegend, clone 259D, cat. 320208, dilution 5:100)
-PerCP/Cy5.5 anti-human Ki67 (Biolegend, clone Ki-67, cat. 350520, dilution 5:100)
-PerCP/Cy5.5 anti-human CD14 (Biolegend, clone M5E2, cat. 301824, dilution 1:100)
-FITC anti-human CD19 (BD Bioscience, clone HIB19, cat. 555412, dilution 1:100)
-APC/Cy7 anti-human CD3 (Biolegend, clone UCHT1, cat. 300426, dilution 1:100)
-BV421 anti-human CD56 (Biolegend, clone 5.1H11, cat. 362551/2, dilution 2:100)
-APC MitoTracker Deep Red (Invitrogen, clone N/A, cat. M22426, dilution 1:1000)
-FITC MitoTracker Green (Invitrogen, clone N/A, cat. M7514, dilution 1:1000)
-Live/Dead Fixable Aqua (Biolegend, clone N/A, cat. 423101/2, dilution 1:1000)

Mouse apoptosis/viability/Treg count/mitochondria:
-FITC Annexin V (Biolegend, clone N/A, cat. 640906, dilution 5:100)
-PE anti-mouse CD25 (Biolegend, clone M-A251, cat. 356103, dilution 5:100)
-BV650 anti-mouse CD4 (Biolegend, clone RM4-5, cat. 100555, dilution 1.5:100)
-PerCP anti-mouse CD8 (Biolegend, clone 53-6.7, cat. 100732, dilution 1.5:100)
-BV510 anti-mouse CD25 (Biolegend, clone PC61, cat. 102042, dilution 5:100)
-APC MitoTracker Deep Red (Invitrogen, clone N/A, cat. M22426, dilution 1:1000)
-FITC MitoTracker Green (Invitrogen, clone N/A, cat. M7514, dilution 1:1000)
-Live/Dead Fixable Violet (ThermoFisher, clone N/A, cat. L34955, dilution 1:1000)

scRNA-seq CITE-seq:
-Biotin anti-human CD25 (Biolegend, clone BC96, cat. 302624, dilution 1:100)
-TotalSeq-C0957 APC Streptavidin (CD25) (Biolegend, clone N/A, cat. 405285, dilution 0.125:100)
-TotalSeq-C0045 anti-human CD4 (Biolegend, clone SK3, cat. 344651, dilution 0.25:100)
-TotalSeq-C0063 anti-human CD45RA (Biolegend, clone HI100, cat. 304163, dilution 2:100)
-TotalSeq-C0088 anti-human CD279 (PD-1) (Biolegend, clone EH12.2H7, cat. 329963, dilution 1:100)
-TotalSeq-C0089 anti-human TIGIT (VS3) (Biolegend, clone A15153G, cat. 372729, dilution 1:100)
-TotalSeq-C0140 anti-human CD183 (CXCR3) (Biolegend, clone G025H7, cat. 353747, dilution 2:100)
-TotalSeq-C0148 anti-human CD197 (CCR7) (Biolegend, clone G043H7, cat. 353251, dilution 2:100)
-TotalSeq-C0151 anti-human CD152 (CTLA-4) (Biolegend, clone BNI3, cat. 369621, dilution 2:100)
-TotalSeq-C0156 anti-human CD95 (Fas) (Biolegend, clone DX2, cat. 305651, dilution 1:100)
-TotalSeq-C0390 anti-human CD127 (IL-7Rα) (Biolegend, clone A019D5, cat. 351356, dilution 1:100)
-TotalSeq-C0250 anti-mouse/human KLRG1 (MAFA) (Biolegend, clone 2F1/KLRG1, cat. 138433, dilution 1:100)
-TotalSeq-C0251 anti-human Hashtag 1 (Biolegend, clone LNH-94, cat. 394663, dilution 0.25:100)
-TotalSeq-C0252 anti-human Hashtag 2 (Biolegend, clone 2M2, cat. 394663, dilution 0.25:100)

CyTOF:
-Anti-human ACAC (Abcam, clone EPR4971, cat. ab231681, dilution 1:100)
-Anti-human ASS1 (Abcam, clone EPR12398, cat. ab231684, dilution 1:50)
-Anti-human ATP5A (Abcam, clone 15H4C4, cat. ab14748, dilution 1:50)
-149Sm anti-human CD127 (Fluidigm, clone 3149011B, cat. 3149011B, dilution 1:50)
-169Tm anti-human CD25 (Fluidigm, clone 2A3, cat. 3169003B, dilution 1:50)

-Anti-human CD25 (Biolegend, clone M-A251, cat. 356102, dilution 1:50)
-141Pr anti-human CD3 (Fluidigm, clone UCHT1, cat. 3141019B, dilution 1:100)
-152Sm anti-human CD36 (Fluidigm, clone 5-271, cat. 3152007B, dilution 1:100)
-Anti-human CD4 (Biolegend, clone RPA-T4, cat. 300501, dilution 1:100)
-162Dy anti-human CD44 (Fluidigm, clone IM7, cat. 3162030B, dilution 1:200)
-89Y anti-human CD45 (Fluidigm, clone HI30, cat. 3089003B, dilution 1:200)
-106Cd anti-human CD45 (Fluidigm, clone HI30, cat. 3106001B, dilution 1:75)
-116Cd anti-human CD45 (Fluidigm, clone HI30, cat. 3116001B, dilution 1:50)
-143Nd anti-human CD45RA (Fluidigm, clone HI100, cat. 3143006B, dilution 1:50)
-153Eu anti-human CD62L (Fluidigm, clone DREG-56, cat. 3153004B, dilution 1:50)
-Anti-human CD69 (Biolegend, clone FN50, cat. 310939, dilution 1:200)
-146Nd anti-human CD8a (Fluidigm, clone RPA-T8, cat. 3146001B, dilution 1:50)
-Anti-human CD98 (Biolegend, clone MEM-108, cat. 315602, dilution 1:50)
-Anti-human CPT1A (Abcam, clone 8F6AE9, cat. ab128568, dilution 1:200)
-Anti-human CXCR6 (Biolegend, clone K041E5, cat. 356002, dilution 1:100)
-Anti-human FABP5 (Novus Biologicals, clone 311214, cat. MAB3077, dilution 1:50)
-Anti-human FoxP3 (Invitrogen, clone PCH101, cat. 14-4776-82, dilution 1:100)
-Anti-human FoxP3 (Biolegend, clone 150D, cat. 320003, dilution 1:100)
-Anti-human FoxP3 (Biolegend, clone 259D, cat. 320202, dilution 1:100)
-Anti-human G6PD (Abcam, clone EPR20668, cat. ab231828, dilution 1:50)
-Anti-human GLUT1 (Abcam, clone EPR3915, cat. ab196357, dilution 1:50)
-Anti-human Helios (Biolegend, clone 22F6, cat. 137202, dilution 1:100)
-Anti-human HK1 (Abcam, clone EPR10134(B), cat. ab233837, dilution 1:100)
-Anti-human IDH2 (Abcam, clone EPR7577, cat. ab230796, dilution 1:200)
-Anti-human Ki67 (Biolegend, clone Ki-67, cat. 350502, dilution 1:100)
-Anti-human LDHA (Abcam, clone EP1566Y, cat. ab219591, dilution 1:50)
-Anti-human MCT1 (Thermo Fisher, clone P14612, cat. MA5-18288, dilution 1:50)
-Anti-human NRF2p (Abcam, clone EPI809Y, cat. ab180844, dilution 1:100)
-Anti-human OPA1 (Abcam, clone EPR11057(B), cat. ab240143, dilution 1:100)

In vitro Treg micro-suppression assay:
-APC-Cy7 anti-human CD4 (BD Biosciences, clone RPA-T4, cat. 55781, dilution 1:50)
-PerCP-Cy5.5 anti-human CD14 (Biolegend, clone HCD14, cat. 325622, dilution 1:50)
-Pacific blue anti-human CD19 (Biolegend, clone HIB19, cat. 302232, dilution 2:50)
-PE anti-human CD25 (BD Biosciences, clone 2A3, cat. 341011, dilution 5:50)
-PE anti-human CD25 (BD Biosciences, clone MA251, cat. 555432, dilution 5:50)
-AF647 anti-human CD127 (BD Biosciences, clone HIL-7R-M21, cat. 558598, dilution 10:50)

Orthotopic HCC mouse model:
-PerCP-Cy5.5 anti-mouse Ly6C (BioLegend, clone HK1.4, cat. 128011, dilution 1:800)
-Alexa Fluor 700 / Brilliant Violet 785 anti-mouse CD45(BioLegend, clone 30-F11, cat. 103128 / 103149, dilution 1:100)
-APC-Fire 750 anti-mouse CD64 (BioLegend, clone X54-5/7.1, cat. 139333, dilution 1:100)
-Brilliant Violet 510 anti-mouse CD11b (BioLegend, clone M1/70, cat. 562950, dilution 1:100)
-Brilliant Violet 605 anti-mouse CD11c (BioLegend, clone N418, cat. 117334, dilution 1:100)
-APC anti-mouse XCR1 (BioLegend, clone ZET, cat. 148206, dilution 1:100)
-Brilliant Violet 785 anti-mouse Ly6G (BioLegend, clone 1A8, cat. 127645, dilution 1:100)
-BUV615 anti-mouse MHC-II (BioLegend, clone M5/114.15.2, cat. 751570, dilution 1:100)
-BUV661 anti-mouse F4/80 (BioLegend, clone T45-2342, cat. 750643, dilution 1:100)
-Alexa Fluor 700 anti-mouse CD8 (BioLegend, clone 53-6.7, cat. 100730, dilution 1:100)
-FITC anti-mouse CD4 (BioLegend, clone RM4-5, cat. 100510, dilution 1:100)
-Brilliant Violet 421 anti-mouse FoxP3 (BioLegend, clone MF-14, cat. 126419, dilution 1:100)
-Brilliant Violet 750 anti-mouse NK1.1 (BioLegend, clone PK136, cat. 746876, dilution 1:100)
-BUV805 / BUV395 anti-mouse CD3 (BioLegend, clone 17A2, cat. 569192 / 740268, dilution 1:100)
-BUV661 anti-mouse γδTCR (BioLegend, clone GL3, cat. 750410, dilution 1:100)
-BUV805 / BUV615 anti-mouse CD19 (BioLegend, clone 1D3, cat. 56287 / 751213, dilution 1:100)
-BUV450 / BUV515 Viability Dye (Thermo Fisher, cat. L34962, dilution 1:1000)

Validation

All antibodies were titrated and tested using appropriate FMO and single colour controls for flow cytometry.
All antibodies had validation statements provided on the website of the manufacturer.

Eukaryotic cell lines

Policy information about cell lines and Sex and Gender in Research

Cell line source(s)	Hep53.4 cell line was purchased from CLS, Eppelheim, Germany, GMBH. Catalog number: 400200
Authentication	STR profiling.
Mycoplasma contamination	Cell lines tested negative to mycoplasma throughout the study.

Commonly misidentified lines (See ICLAC register)	No commonly misidentified cell lines were used in this study.

Palaeontology and Archaeology

Specimen provenance	N/A
Specimen deposition	N/A
Dating methods	N/A

☐ Tick this box to confirm that the raw and calibrated dates are available in the paper or in Supplementary Information.

Ethics oversight	N/A

Note that full information on the approval of the study protocol must also be provided in the manuscript.

Animals and other research organisms

Policy information about studies involving animals; ARRIVE guidelines recommended for reporting animal research, and Sex and Gender in Research

Laboratory animals	Mice were housed in a specific pathogen-free condition in accordance with institutional guidelines and ethical regulations.<br><br>Mouse strains:<br>-C57BL/6JOlaHsd wild-type mice were purchased from Envigo.<br>-NRF2 KO mice were provided by Dr. Giovanni E. Mann (Kings College London).<br>-FOXP3-Cre+/+ Nrf2fl/- mice were developed during the study at Kings College London.<br>-FOXP3-Cre+/+ Nrf2fl/fl mice were developed during the study at Kings College London.<br><br>Strain, age and sex of mice:<br>-C57BL/6J wild-type and NRF2 KO mice (6-10 weeks old, female and male)<br>-FOXP3-Cre+/+ Nrf2fl/- and FOXP3-Cre+/+ Nrf2fl/fl mice (10-15 weeks old, female)<br><br>Housing conditions: mice were housed under standard specific pathogen–free conditions, with a 12-hour light/12-hour dark cycle, an ambient temperature of 20–24°C, and relative humidity maintained between 45–65%. Food and water were provided ad libitum. Experimental and control mice were co-housed.
Wild animals	N/A
Reporting on sex	The study involved the use of a tamoxifen-inducible Treg-specific Nrf2-knockdown mice and findings only apply to female mice. In this model, only female mice are homozygous in the FOXP3-Cre and Nrf2 fl/fl alleles which have the absolute effect of Treg-specific Nrf2-knockdown upon tamoxifen administration. For the orthothopic and MASH-HCC mouse models, only adult male mice were used. For the in vitro experiments, samples from both male and female C57BL/6J wild-type and NRF2 KO mice were used.
Field-collected samples	This study did not involve samples collected from the field.
Ethics oversight	The study involved animals, and all procedures related to animal work were performed in accordance with all legal, ethical, and institutional requirements (PP4067431 and PP8854860) as dictated by UK legislation and ethical review committees at King's College London and Newcastle University.

Note that full information on the approval of the study protocol must also be provided in the manuscript.

Clinical data

Policy information about clinical studies

All manuscripts should comply with the ICMJE guidelines for publication of clinical research and a completed CONSORT checklist must be included with all submissions.

Clinical trial registration	N/A
Study protocol	N/A
Data collection	N/A
Outcomes	N/A

Dual use research of concern

Policy information about dual use research of concern

Hazards

Could the accidental, deliberate or reckless misuse of agents or technologies generated in the work, or the application of information presented in the manuscript, pose a threat to:

No	Yes	
☒	☐	Public health
☒	☐	National security
☒	☐	Crops and/or livestock
☒	☐	Ecosystems
☒	☐	Any other significant area

Experiments of concern

Does the work involve any of these experiments of concern:

No	Yes	
☒	☐	Demonstrate how to render a vaccine ineffective
☒	☐	Confer resistance to therapeutically useful antibiotics or antiviral agents
☒	☐	Enhance the virulence of a pathogen or render a nonpathogen virulent
☒	☐	Increase transmissibility of a pathogen
☒	☐	Alter the host range of a pathogen
☒	☐	Enable evasion of diagnostic/detection modalities
☒	☐	Enable the weaponization of a biological agent or toxin
☒	☐	Any other potentially harmful combination of experiments and agents

Plants

Seed stocks: N/A

Novel plant genotypes: N/A

Authentication: N/A

ChIP-seq

Data deposition

☐ Confirm that both raw and final processed data have been deposited in a public database such as GEO.

☐ Confirm that you have deposited or provided access to graph files (e.g. BED files) for the called peaks.

Data access links
May remain private before publication.: N/A

Files in database submission: N/A

Genome browser session
(e.g. UCSC): N/A

Methodology

Replicates: N/A

Sequencing depth: N/A

Antibodies: N/A

Peak calling parameters: N/A

Data quality: N/A

Software: N/A

Flow Cytometry

Plots

Confirm that:

☒ The axis labels state the marker and fluorochrome used (e.g. CD4-FITC).

☒ The axis scales are clearly visible. Include numbers along axes only for bottom left plot of group (a 'group' is an analysis of identical markers).

☒ All plots are contour plots with outliers or pseudocolor plots.

☒ A numerical value for number of cells or percentage (with statistics) is provided.

Methodology

Sample preparation

Freshly resected human liver and tumour samples were processed by enzymatic digestion with the BD HorizonTM Dri Tumor & Tissue Dissociation Reagent (TTDR) (BD Biosciences) as per manufacturer instructions. After digestion, intra-hepatic or intra-tumoral lymphocytes were isolated as described in Kucykowicz, S. et al. STAR Protoc. 3, 101356 (2022), and used fresh or stored in CryoStor® cell preservation media (Sigma-Aldrich).

Cells from human spleen and lymph nodes obtained from healthy liver donors were isolated by mechanic tissue disruption, and red blood cells were lysed with RBC lysis buffer (Biolegend).

Human PBMCs were isolated from heparinised blood by density centrifugation using Ficoll-Paque PLUS (Sigma-Aldrich). PBMCs were used fresh or were frozen in CryoStor® cell preservation media (Sigma-Aldrich) and stored until the moment of analysis.

Murine cells were isolated from the spleen and lymph nodes by mechanic tissue disruption, and red blood cells were lysed with RBC lysis buffer (Biolegend).

Mouse tumours were digested using a mouse tumour dissociation kit in GentleMACS C digestion tubes with a GentleMACS tissue dissociator (Miltenyi Biotec) and single cell suspension generated by passing through a 70um filter. Cells were layered onto 30% Percoll gradient and pelleted RBCs lysed.

Instrument

BD LSR Fortessa and BD Symphony A5 flow cytometers.

Software

For data collection: BD FACSDiva software (version 9)
For data analysis: FlowJo (version 10.7.1)

Cell population abundance

The frequency of the purity for the populations of interest are specified in the appropriate figures/methods.

Gating strategy

Cells were initially gated for FSC/SSC, with doublets excluded using FSC and SSC A/H parameters. Live cells were then identified, followed by population-specific markers.

☐ Tick this box to confirm that a figure exemplifying the gating strategy is provided in the Supplementary Information.

Magnetic resonance imaging

Experimental design

Design type: N/A

Design specifications: N/A

Behavioral performance measures: N/A

Acquisition

Imaging type(s)	N/A
Field strength	N/A
Sequence & imaging parameters	N/A
Area of acquisition	N/A

Diffusion MRI ☐ Used ☒ Not used

Preprocessing

Preprocessing software	N/A
Normalization	N/A
Normalization template	N/A
Noise and artifact removal	N/A
Volume censoring	N/A

Statistical modeling & inference

Model type and settings	N/A
Effect(s) tested	N/A

Specify type of analysis: ☐ Whole brain ☐ ROI-based ☐ Both

Statistic type for inference (See Eklund et al. 2016)	N/A
Correction	N/A

Models & analysis

n/a	Involved in the study
☒	☐ Functional and/or effective connectivity
☒	☐ Graph analysis
☒	☐ Multivariate modeling or predictive analysis
