# Supplementary material for: Nrf2-mediated metabolic reprogramming drives regulatory T cell accumulation in hepatocellular carcinoma
Source: Nat Commun. 2026 Jul 13;17:8639. doi: 10.1038/s41467-026-73485-3 (PMC13487165; doi:10.1038/s41467-026-73485-3)
Supplement: Supplementary file 4 [file 41467_2026_73485_MOESM5_ESM.pdf]

nature portfolio

Peer Review File

Nrf2-mediated metabolic reprogramming drives regulatory T cell accumulation in hepatocellular carcinoma

Corresponding Author: Dr Niloufar Safinia

This file contains all reviewer reports in order by version, followed by all author rebuttals in order by version.

Version 0:

Reviewer comments:

Reviewer #1

(Remarks to the Author)
The Authors investigate the molecular and metabolic program of tissue resident Tregs in hepatocellular carcinoma (HCC) and non-tumor/healthy liver tissue.
They report that while non-tumoral Tregs are metabolically inactive and are prone to apoptosis, HCC Tregs are metabolically active as a result of Nrf2 activation that is linked to Treg survival and function. The role of Nrf2 in favoring HCC Tregs in supported by *in vivo* experiments showing that loss of function of this transcription factor, either through Nfe212 ablation or pharmacologic inhibition, limits intra-tumoral Treg accumulation and suppresses tumor growth. In addition to providing new insights on the role of Nrf2 in governing Treg metabolism in HCC, the findings are translationally relevant by proposing blockade of Nrf2 as a new therapeutic approach for HCC.
There are aspects of the proposed work that require attention and further consideration.
• Results in Fig. 1 should be presented in the text in a more concise and clearer way. The Authors use the terms ‘healthy liver’ and ‘HCC background tissue’ interchangeably. Moreover, it is not clear as to why results from ‘healthy’ liver peripheral blood are included in the main figure while those of peripheral blood paired with HCC liver are presented in Supplementary Fig. 1. Unless there is a strong scientific rationale for it, I would recommend presenting the data once, to avoid redundancy.
• Markers defining Tregs in Fig. 1A should be provided. A brief explanation of the CD45RA^{+}Foxp3^{lo}, CD45RA^{-}Foxp3^{lo} and CD45RA^{-}Foxp3^{hi} subsets is encouraged.
• Fig. 1H: Tregs from HCC liver display a stronger suppression when compared to Tregs from healthy liver. However, a statistically significant difference is noted at 1:2, which is not the typical Treg/Teff physiological ratio (i.e., 1:8-1:9). Given that a difference statistically significant is not found even at 1:4 ratio when comparing Tregs from different compartments, the extent to which metabolic signatures impact Treg function *ex vivo* remains unclear.
• What is the difference between the findings in Supplementary Fig. 1G-H and Supplementary Fig. 2 B-C?
• The Authors might consider attenuating some of the statements related to transcriptome analysis. As an example, unless supported by experimental evidence, the conclusion on page 11 (lines 222-224) that TCR engagement is key for Treg metabolic adaptation and survival, should be mitigated.
• When reporting findings on metabolically active and inactive Tregs (clusters G and C of liver resident Tregs from non-tumor tissue), to what extent are these metabolic differences important for Treg suppression *ex vivo*?
• How do TIL Tregs maintain suppressor capabilities despite being characterized by increased expression of genes related to glycolysis and ATP production?
• Do healthy CD4 cells exposed to lactate preserve their suppression?
• Do liver Tregs from Nrf2 KO mice display metabolic changes comparable to those noted in human HCC?

Reviewer #2

(Remarks to the Author)
The manuscript by Perpiñán et al addresses a timely and important question in cancer immunology by identifying Nrf2 as a key regulator of intra-tumoral Treg survival and function in hepatocellular carcinoma (HCC). The study provides novel insights into how lactate-rich tumor microenvironments promote Treg-mediated immunosuppression via Nrf2-driven metabolic adaptation. The use of both genetic and pharmacological approaches to target Nrf2 strengthens the mechanistic claims and highlights translational potential. That said, the ultimate impact of this work will hinge on the depth of mechanistic investigation and the robustness of in vivo models. More specific comments are as follows:

1. Your study identifies Nrf2 as a critical regulator of intratumoral Treg survival and function in HCC, suggesting its inhibition as a therapeutic strategy. However, prior studies have shown context-dependent roles of Nrf2 in Tregs. For instance, Klemm et al. (EJI, 2019) reported that constitutive Nrf2 activation via Keap1 deletion destabilizes Tregs and leads to autoimmunity, while a more recent study (Front. Immunol., 2024) showed that

pharmacological Nrf2 activation by dimethyl fumarate enhances Treg differentiation and function in allergic asthma. Given these contrasting outcomes, could the authors speculate on how the TME-specific metabolic context (e.g., lactate-induced oxidative stress) uniquely shapes Nrf2 function in Tregs in HCC, and how this might differ from homeostatic or inflammatory settings such as allergy? Additionally, how do the authors envision the therapeutic window for Nrf2 inhibition, given its potentially beneficial role in peripheral immune tolerance?
2. Specific for Fig. 1H, given the differences in Treg shown in TILs vs NILs and PBMCs, can the authors explain why the Treg in TILs is only significantly more immunosuppressive vs those in NILs but not PBMCs? In addition, It would also be helpful to discuss the potential mechanisms driving the differences in Tregs phenotypes, particularly the role of IL-2 availability in more detail e.g. by providing analysis of IL-2 level to support the speculation.
3. For the scRNA seq data, if the focus on this study is to decipher phenotypes/ function of Tregs in TILs, why is the scRNA seq analysis conducted only on blood and paired healthy livers but not HCC tumours? Also, it is not clear how Clusters 3, 8, 11, 13 and 15 corresponded to Tregs, the authors should provide expression of key Treg genes, particularly Foxp3, in UMAP or Dot Plot to confirm the annotation.
4. The scRNA seq analysis related to Fig. 2 is largely descriptive and speculative. For instance it is not clear of the implications of these differences based on genes enrichment or TCR repertoire analysis. For instance, TCR overlap analysis is supportive but not definitive in speculation on Treg trajectory. In fact, a trajectory analysis will be more helpful in this conclusion. Second, the pseudo-bulk approach is acceptable, but a more granular analysis (e.g., module scoring across clusters or pathway-level analysis per Treg subset) would provide greater resolution. Most importantly, the narrative is somewhat confusing: the initial observations focus on TIL-Tregs, but the subsequent mechanistic analyses (e.g., scRNA-seq) shift to intrahepatic Tregs from healthy liver tissue, without addressing TIL-Tregs in the same depth (same comment as in #3). Furthermore, the added value of subclustering Treg from Tconv, which is already a highly specific subset of $CD4^+$ T cells, is not clear.
5. Finally the analysis based on TILs-Treg is provided in Fig.3. In my opinion, Integrating all three compartments: TILs, NILs and PBMCs in a unified analysis (instead of providing redundant analysis in Fig. 2 & 3) would have provided a more comprehensive and physiologically relevant understanding of Treg heterogeneity and dynamics in HCC. In addition, same issues as comment in #3, the authors should provide expression of key Treg genes in UMAP or Dot Plot to justify the clusters annotation. Furthermore, this analysis is again highly speculative without any functional evidence. The interpretation that TIL Tregs are oligoclonally expanded and distinct from non-tumoral liver Tregs is also based on TCR overlap but lacks depth in clonal trajectory analysis.
6. Can the authors provide justification of the 64-gene signature for Cluster B' Tregs as it lacks typical Treg-feature genes like Foxp3?
7. The design of panel for Mass Cytometry in Fig. 5 is not clearly explained. How did the authors select those markers for analysis? The flow of the earlier discovery to Nrf2 seems rather sudden and without clear justification. Also, among other potential targets, picking Nrf2 for downstream analysis is rather strange and somewhat bias/cherry-picking.
8. The aim of introduction of H2O2 in Fig. 6 is not clear. How is that relevant to the earlier findings/conclusion?
9. The comprehensive TILs analysis e.g. effector (CD8+ T and NK cells) immune cell subsets on Treg-specific Nrf2 KD model is lacking. The similar analysis like those provided in Fig. 7I is important to explain the effect on tumour burden in this model.
10. Histology analysis on mice fed with western diet should be provided to confirm MAFLD conditions in the liver.

Reviewer #3

(Remarks to the Author)
Perpinan et al. analyzed metabolic adaptions of Tregs in HCC. By analysing human tissue Tregs from HCC and non-tumoral liver tissue, they found activation of Nrf2 in Tregs promoting Treg survival and function. In mouse models they reveal that Nrf2 deletion prevents Treg accumulation in HCC and reduces tumor growth.
The study is well performed and includes a comprehensive analysis of human tissue Tregs from different origins as well as proof-of-principle mice experiments. In addition the topic is of high relevance.
However there are some pointa that need to be adressed before considering the study for publication.
authors show that Tregs in non-tumoral liver tissue undergo apoptosis. The liver is also a storage for iron. So, is it apoptosis or ferroptosis ?
For me it is not clear by which mechanism Tregs behave different from T conv in HCC. Tregs are normally metabolically less active that Tconv. While HCC Tregs show an increased metabolic activity. So there has to be a fundamental switch.Can authors show why the MCT-1 lactate uptake is higer in Tregs? Is lactate inducing NRF2 by Keap1-Lactylation or is NRF2 inducing MCT-1? Can authors answer this by means of the NRF2-KO mice? Or at least discuss this point. It would also help to underpin the metabolic profiling of HCC Tregs by means of SCENITH assays. Also the role for NRF2 in mitochondrial regulation could by analyzed more in detail, to see if all or one complex of the electron transport chain is affected in NRF2 deficient mice and also in HCC Tregs. This can be analyzed by using the method of Erny et al. (STAR PROTOCOLS, 2022).
Minor Points:
In figure 1. the gating strategy is not clear to me. authors should describe in detail how they gate on Tregs and are sure not to include T conv the Treg gates. The sorting strategy also needs to be clarified.
fig. 1 A-E the lymph node data are from 3 donors and show high variation. This schould be interpreted with caution.
NRF2 activation should in addition to flow cytometric analysis of NRF2p also be assesed by WB of NRF2 or its target gene NQO1
Fig.6: Were these experiments only performed with 3 mice in each group? Please use at least 5 mice when you want to show clear differences.
6A: Why did authors gate on HO-1 here and not NQO1, which is knwon to be the high specific NRF2 target gene

Discussion
-There is a study from Maj et al.(Nat. Immun. 2017) which in contrast to this study showed that tumor Tregs are more vulnerable for apoptosis and have a weak anti-oxidant system. Authors should discuss this study and the differences to their findings.
- authors should mention limitations of the study
- authors state that it is benefecial to target NRF2 especially in inflammed HCC. While this might be true for established tumors, authors should also mention that targeting Tregs and or NRF2 can also have dramatic effects in inflammation, such as NASH.

Version 1:

Reviewer comments:

Reviewer #1

(Remarks to the Author)
N/A

Reviewer #2

(Remarks to the Author)

This reviewer acknowledges the authors' efforts in revising the manuscript and addressing previous comments. However, the revised data should be included in the rebuttal letter to facilitate review. There are also several errors and remaining issues that require attention:

#3. There are errors in the references to supplementary figures. For example, the expression of Treg-specific genes is shown in Fig. S25 rather than Fig. S24, and the histopathological evaluation of MASH in the animal model appears in Fig. S18 instead of Fig. S17. The authors are advised to carefully check and confirm the accuracy and order of all supplementary figures.
#4. The trajectory analysis plot seems unconventional, and the directionality of the trajectory is unclear. Furthermore, the gene expression or pathway differences leading to the two distinct fates (E vs. G) are not analysed or illustrated. Please clarify the trajectory direction and include data or discussion that elucidates the molecular or pathway distinctions between these two fates.

Reviewer #3

(Remarks to the Author)
The authors have adequately adressed my comments.

Version 2:

Reviewer comments:

Reviewer #2

(Remarks to the Author)
Thank u for the effort in addressing my comments. I do believe with the additional data provided, the manuscript is now suitable for publication in Nature Communications.

Open Access This Peer Review File is licensed under a Creative Commons Attribution 4.0 International License, which permits use, sharing, adaptation, distribution and reproduction in any medium or format, as long as you give appropriate credit to the original author(s) and the source, provide a link to the Creative Commons license, and indicate if changes were made.
In cases where reviewers are anonymous, credit should be given to 'Anonymous Referee' and the source.
The images or other third party material in this Peer Review File are included in the article's Creative Commons license, unless indicated otherwise in a credit line to the material. If material is not included in the article's Creative Commons license and your intended use is not permitted by statutory regulation or exceeds the permitted use, you will need to obtain permission directly from the copyright holder.
To view a copy of this license, visit https://creativecommons.org/licenses/by/4.0/

Reviewer 1:

1) *"Results in Fig. 1 should be presented in the text in a more concise and clearer way. The Authors use the terms 'healthy liver' and 'HCC background tissue' interchangeably. Moreover, it is not clear as to why results from 'healthy' liver peripheral blood are included in the main figure while those of peripheral blood paired with HCC liver are presented in Supplementary Fig. 1. Unless there is a strong scientific rationale for it, I would recommend presenting the data once, to avoid redundancy".*

We are grateful to Reviewer 1 to pointing this out. We have unified **Fig.1** and **Fig.S1** and amended our manuscript to provide a more concise description of the results. "Healthy liver" and "background HCC tissue" correspond to different specimens – we have made sure that these terms are used accurately and not interchangeably throughout the manuscript.

2) *"Markers defining Tregs in Fig. 1A should be provided. A brief explanation of the CD45RA$^+$Foxp3^{lo}, CD45RA$^-$Foxp3^{lo} and CD45RA$^-$ Foxp3hi subsets is encouraged".*

The gating strategy is provided in the new **Fig.S1** The figure legend provides a brief description of the *CD45RA$^+$Foxp3^{lo}, CD45RA$^-$Foxp3^{lo} and CD45RA$^-$ Foxp3hi subsets.* Further information is included in the Material & Methods section.

3) *"Fig. 1H: Tregs from HCC liver display a stronger suppression when compared to Tregs from healthy liver. However, a statistically significant difference is noted at 1:2, which is not the typical Treg/Teff physiological ratio (i.e., 1:8-1:9). Given that a difference statistically significant is not found even at 1:4 ratio when comparing Tregs from different compartments, the extent to which metabolic signatures impact Treg function ex vivo remains unclear".*

While we agree with Reviewer 1 that a Treg:Teff of 1:2 could be considered as unphysiological, many other studies have used similarly high ratios (see Collison LW et al. Methods Mol Biol 2011). Furthermore, extrapolating from different studies is complex, given that the Treg:Teff ratios needed to demonstrate immunosuppressive effects vary substantially depending on the specific experimental conditions employed. In our case, due to the very small number of Tregs that could be isolated from clinical specimens, we employed a 'micro-suppression' assay developed by our collaborator Prof. Tim Tree whose experimental conditions differ from those used in more 'conventional' suppression assays (from which the typical Treg/Teff ratios Reviewer 1 is referring to were derived). Altogether, we believe that the 'area under the curve' analysis also provided in **Fig.1H** is a much more reliable marker of the overall Treg suppressive capacity given that it integrates suppression values from all the Treg:Teff ratios employed.

4) *"What is the difference between the findings in Supplementary Fig. 1G-H and Supplementary Fig. 2 B-C?"*

Reviewer 1 is right to point out that there was a degree of redundancy between Supplementary Fig. 1G-H and Supplementary Fig. 2B-C. To correct this and avoid potential confusions, all data have now been consolidated into **Fig. 1I**.

5) *"The Authors might consider attenuating some of the statements related to transcriptome analysis. As an example, unless supported by experimental evidence, the conclusion on page 11 (lines 222-224) that TCR engagement is key for Treg metabolic adaptation and survival, should be mitigated".*

We are grateful to Reviewer 1 for pointing this out. We have amended our manuscript to provide more nuanced statements whenever only descriptive data (e.g. scRNASeq) is available to support our claims.

6) *"When reporting findings on metabolically active and inactive Tregs (clusters G and C of liver resident Tregs from non-tumor tissue), to what extent are these metabolic differences important for Treg suppression ex vivo?".*

We assume that Reviewer 1 is referring to clusters G and E, the two main clusters of tissue-resident Tregs detected in non-tumoral tissue. Unfortunately, we cannot formally answer Reviewer 1's question given the small number of Tregs that can be isolated from surgical resections, which precludes the performance of detailed functional experiments on sorted Treg sub-populations. Based

on their metabolic programmes, however, we would speculate that cluster G Tregs would be more immunosuppressive than cluster E Tregs (of note, in terms of their enrichment in metabolic pathways, clusters C and G were closer than clusters G and E, despite the fact that cluster C did not exhibit tissue-resident features). Our speculation is based on published literature demonstrating that mitochondrial integrity, OXPHOS, and fatty acid oxidation are critically involved in the maintenance of Treg suppression (e.g. Michalek et al. Journal of Immunology 2011; Priyadharshini et al. Journal of Immunology 2018; Beier et al. FASEB Journal 2015; Zhao et al. Mitochondrion 2025; Blagih et al. Frontiers in Immunology 2021; Angelin et al. Cell Metabolism 2017). Our own results comparing the properties of HCC TIL Tregs with those of paired background HCC tissue further support our assumptions. HCC TIL Tregs, which exhibited highly active glycolytic and OXPHOS metabolic pathways, were found to exert much more powerful suppressive effects *ex vivo* than Tregs from non-tumoral tissue.

7) *"How do TIL Tregs maintain suppressor capabilities despite being characterized by increased expression of genes related to glycolysis and ATP production?"*

We thank Reviewer 1 for raising this important question, which is interconnected the previous one (point 6). Tregs exhibit a remarkable metabolic flexibility that allows them to maintain the energy production and biosynthetic capacity necessary for effective immune suppression across a variety of environmental conditions (de Candia et al. Immunity 2022). On the one hand, as pointed out by Reviewer 1, multiple evidences indicate that mitochondrial integrity, OXPHOS, and fatty acid oxidation are critically involved in the maintenance of Treg suppression (e.g. Michalek et al. Journal of Immunology 2011; Priyadharshini et al. Journal of Immunology 2018; Beier et al. FASEB Journal 2015; Zhao et al. Mitochondrion 2025; Blagih et al. Frontiers in Immunology 2021). This includes Tregs residing in glucose-deplete, lactate-rich, tumour microenvironments (Angelin et al. Cell Metabolism 2017). On the other hand, there is also data indicating that the viability, proliferative, and suppressive capacity of tumour-infiltrating Tregs depends on the activation of glycolytic pathways as well fatty acid metabolism (Pacella et al. PNAS 2018). Pacella et al. used gene expression and Seahorse analyses to demonstrate that tumour-infiltrating Tregs simultaneously engage glycolysis, fatty acid synthesis and OXPHOS, which allows them to maintain a higher proliferative capacity than conventional T cells and at the same time retain powerful immunosuppressive properties. Our findings indicating that the accumulation of viable and highly suppressive Tregs within the HCC microenvironment is associated with the activation of both glycolytic and OXPHOS pathways is well in line with the results of Pacella et al. This has now been specifically highlighted in the Discussion section.

8) *"Do healthy CD4 cells exposed to lactate preserve their suppression?"*

Yes, multiple published studies have shown that healthy CD4+ regulatory T cells exposed to lactate are able to preserve, and in some contexts even enhance, their suppressive function. This is the reason why we did not consider necessary to undertake this specific experiment in our study. Relevant references include: Watson et al. Nature 2021; Angelin et al. Cell Metabolism 2017; Zhou et al. J Clin Invest 2024; de Kivit et al. Cell Reports 2024.

9) *"Do liver Tregs from Nrf2 KO mice display metabolic changes comparable to those noted in human HCC?*

Yes, our experiments assessing mitochondrial functionality and fitness reveal that there are striking similarities between the Nrf2 KO mice and what is observed in human HCC samples. This is clearly shown by the MitoTracker Green and Red staining, which provide an estimate of the mitochondrial mass and membrane potential, respectively. Nrf2 KO Tregs exhibited reduced fluorescence intensity for both dyes as compared to WT Tregs (**Fig.6M** and **N**). This suggests that in the absence of Nrf2, Tregs have lower mitochondrial content and more depolarized mitochondrial membranes, indicative of impaired mitochondrial function. In contrast, Tregs from HCC tissues show enhanced mitochondrial fitness, as evidenced by high MitoTracker Green and Red signal intensities (**Fig.6O**), reflecting increased mitochondrial mass and preserved membrane polarization. These findings are further supported by the results of the single-cell RNA sequencing data shown in **Fig.3E**.

Please note that, to ensure consistency in data representation, **Fig. 6O** has been updated to display MitoTracker MFI values for HCC Tregs, aligning with the format used for WT and Nrf2 KO Tregs and Tconv cells.

Reviewer 2:

1) *"Your study identifies Nrf2 as a critical regulator of intratumoral Treg survival and function in HCC, suggesting its inhibition as a therapeutic strategy. However, prior studies have shown context-dependent roles of Nrf2 in Tregs. For instance, Klemm et al. (EJI, 2019) reported that constitutive Nrf2 activation via Keap1 deletion destabilizes Tregs and leads to autoimmunity, while a more recent study (Front. Immunol., 2024) showed that pharmacological Nrf2 activation by dimethyl fumarate enhances Treg differentiation and function in allergic asthma. Given these contrasting outcomes, could the authors speculate on how the TME-specific metabolic context (e.g., lactate-induced oxidative stress) uniquely shapes Nrf2 function in Tregs in HCC, and how this might differ from homeostatic or inflammatory settings such as allergy? Additionally, how do the authors envision the therapeutic window for Nrf2 inhibition, given its potentially beneficial role in peripheral immune tolerance?".*

We thank Reviewer 2 for this insightful question regarding the context-dependent roles of Nrf2 in Tregs. As hinted by the reviewer, we believe that the apparent contradictions in the literature can be reconciled by considering the unique metabolic and oxidative stress conditions within the tumour microenvironment (TME) that distinguish it from homeostatic or alternative inflammatory settings. Our data indicate that one of the candidate metabolites, lactate, which is found at high concentration within the TME (Kim EY et al. Molecules (Basel) 2025; Barba I et al. Int J of Mol Sci 2024; Pérez-Tomás R et al. Cancers 2020; Jin M et al. Frontiers in Cell and Develop Biology 2022; Gao Y et al. J of Immunol Res 2022; Tan S et al. Int J of Mol Sci 2025; Romero-Garcia S et al. Frontiers in Immunol 2016) induces lactoylation of Keap1, leading to Nrf2 stabilization and activation. This post-translational modification represents a metabolic adaptation mechanism that is specific to the lactate-rich TME and that differs fundamentally from both the genetic perturbations studied by Klemm et al. and the pharmacological activation examined in allergic asthma models. We hypothesize that the key distinction lies in the stimulus-response context: constitutive genetic activation through Keap1 deletion creates sustained, supraphysiological, Nrf2 activation that disrupts normal cellular homeostasis, while TME-induced lactoylation provides a proportionate, stress-responsive, activation that maintains Treg viability under hostile conditions. Similarly, pharmacological Nrf2 activation in allergic asthma operates in a different metabolic context, in which glucose availability and lactate concentrations differ markedly from what is found in the TME.

Regarding the therapeutic window for Nrf2 inhibition, we acknowledge the potential risks of systemic inhibition, given Nrf2's key anti-oxidant function and the role in peripheral immune tolerance that our work has elucidated. However, several emerging strategies could provide tumour-selective targeting, such as: 1) the use of Nrf2 inhibitors designed as inactive compounds that are selectively activated within the TME (e.g. in response to the altered pH conditions present in tumours); 2) Nrf2 inhibitors could be administered directly into the HCC tumours (e.g. through transarterial chemoembolization (TACE), which is routinely employed as treatment for HCC); 3) the use of proteolysis-targeting chimeras (PROTACs) designed for tumour-selective degradation of Nrf2 (Ji J et al. J of Med Chem 2023).

We have provided clarifications on these topics in the amended version of the manuscript's Discussion.

2) *"Specific for Fig. 1H, given the differences in Treg shown in TILs vs NILs and PBMCs, can the authors explain why the Treg in TILs is only significantly more immunosuppressive vs those in NILs but not PBMCs? In addition, It would also be helpful to discuss the potential mechanisms driving the differences in Tregs phenotypes, particularly the role of IL-2 availability in more detail e.g. by providing analysis of IL-2 level to support the speculation".*

We thank Reviewer 2 for this comment. While it is true that no statistically significant differences were observed between TIL and PBMC Tregs, **Fig 1H** shows a strong trend in favour of TIL Tregs (as compared to both PBMC and healthy liver Tregs). Furthermore, *in vitro* suppression assays are extremely reductionistic in that they do not capture the whole array of immunosuppressive mechanisms exerted by Tregs. Indeed, the higher activation status and expression levels of phenotypic

markers such as CTLA-4, TIGIT, CD95 and PD-1 noted on TIL Tregs indicate that these cells are likely to be substantially more immunosuppressive than those detected in other tissue compartments.

In response to Reviewer 2's request we have conducted additional RNASeq and cytokine array experiments to better characterize the inflammatory environment of tumoral and non-tumoral liver tissue and identify potential drivers of HCC TIL Treg accumulation/fitness. These experiments (provided as Supplementary Fig. 10) have revealed no differences between the 2 tissue compartments in what regards conventional Treg growth factors, which further support our conclusion that Treg metabolic adaptations are key for their expansion within the tumour microenvironment.

3) *"For the scRNA seq data, if the focus on this study is to decipher phenotypes/ function of Tregs in TILs, why is the scRNA seq analysis conducted only on blood and paired healthy livers but not HCC tumours? Also, it is not clear how Clusters 3, 8, 11, 13 and 15 corresponded to Tregs, the authors should provide expression of key Treg genes, particularly Foxp3, in UMAP or Dot Plot to confirm the annotation".*

The objectives of our study were to identify the mechanisms through which Tregs adapt to the liver microenvironment and determine whether/how these mechanisms are modified once liver cancer arises (see Introduction [page 6] and Study Design [page 24] sections). This is the reason why we first compared blood and paired healthy liver, and only afterwards analysed the differences between cancerous and non-cancerous liver tissue.

As requested, we now provide dot plots showing the expression of Foxp3 and other key genes to confirm the identity of the Treg clusters (see **Fig.S24**).

4) *"The scRNA seq analysis related to Fig. 2 is largely descriptive and speculative. For instance it is not clear of the implications of these differences based on genes enrichment or TCR repertoire analysis. For instance, TCR overlap analysis is supportive but not definitive in speculation on Treg trajectory. In fact, a trajectory analysis will be more helpful in this conclusion. Second, the pseudo-bulk approach is acceptable, but a more granular analysis (e.g., module scoring across clusters or pathway-level analysis per Treg subset) would provide greater resolution.*

We are grateful for Reviewer 2's comments. As suggested, we have now conducted pseudotime trajectory analyses employing the *scFates* package to strengthen the conclusions of our original TCR repertoire overlap analyses. The results of these new analyses (provided as **Fig.S7**) confirm that clusters E and G (liver) constitute different cell states that derive from cluster C (blood), with cluster E being in closer continuity with circulating Tregs than cluster G. We believe these results support our hypothesis that circulating Tregs face 2 different fates when they reach the liver: they either become metabolically quiescent and prone to apoptosis (cluster E) or they become metabolically active and clonally expand (cluster G), presumably as a result of being able to recognize cognate antigens.

5) *"Most importantly, the narrative is somewhat confusing: the initial observations focus on TIL-Tregs, but the subsequent mechanistic analyses (e.g., scRNA-seq) shift to intrahepatic Tregs from healthy liver tissue, without addressing TIL-Tregs in the same depth (same comment as in #3). Furthermore, the added value of subclustering Treg from Tconv, which is already a highly specific subset of CD4⁺ T cells, is not clear.*

The narrative of the manuscript follows the objectives of the study, which were: 1) to identify the key determinants of Treg liver tissue residency; 2) to investigate how these processes are impacted by HCC development; and 2) to elucidate the contribution of Nrf2 signalling to the homeostasis of intra-tumoral Tregs and to the progression of HCC. This is the reason why, after describing the differential accumulation of Tregs in non-tumoral and tumoral liver tissue as compared to other compartments, our scRNASeq experiments were first focused on the differences between Tregs residing on healthy liver and those found in the circulation. Having determined that liver tissue Tregs exhibit some unique features, we investigated the extent to which these features change when primary liver cancer develops. We apologise if this did not come across clearly enough. We have amended the manuscript to highlight the study's objectives and we have provided more clarity as to the narrative (see Introduction [page 6], Results [page 13], and Study Design [page 24] sections).

We decided to re-cluster the cells that we had identified as Tregs to ensure that the resulting Treg clusters would be driven by differences in the Treg transcriptome, rather than by major transcriptional differences between Tregs and Tconv (which is what accounts for the differences observed in the UMAP shown in Fig.S3A). We believe this is standard practice in the scRNASeq field, although we agree

that it increases the complexity of the results. We have carefully reviewed our manuscript and added additional clarifications were needed to facilitate the reading.

6) *"Finally the analysis based on TILs-Treg is provided in Fig.3. In my opinion, Integrating all three compartments: TILs, NILs and PBMCs in a unified analysis (instead of providing redundant analysis in Fig. 2 & 3) would have provided a more comprehensive and physiologically relevant understanding of Treg heterogeneity and dynamics in HCC. In addition, same issues as comment in #3, the authors should provide expression of key Treg genes in UMAP or Dot Plot to justify the clusters annotation. Furthermore, this analysis is again highly speculative without any functional evidence. The interpretation that TIL Tregs are oligoclonally expanded and distinct from non-tumoral liver Tregs is also based on TCR overlap but lacks depth in clonal trajectory analysis".*

We agree with Reviewer 2 that, rather than conducting 2 separate sets of scRNASeq analyses, it would have been more informative to integrate all tissue compartments (i.e. blood, non-tumoral liver tissue, tumoral liver tissue) into one single analysis. We did conduct an analysis of all compartments grouped together (i.e. PBMC, healthy liver, background HCC liver, and HCC), but had to discard it because the PBMC compartment hindered the overall data integration and prevented us from simultaneously dealing with the heterogeneity of patients and compartments.

As can be seen in panel A of the figure below (which includes all samples pooled together), an integration step was necessary to mitigate the batch effects from the different patients. When we attempted to remove the patient batch effect on the whole dataset, most tissue compartments remained separated (panel B), which would have interfered with any subsequent analyses (i.e. most Treg clusters would have been compartment-specific). In contrast, when we removed the PBMC compartment, data integration was much more successful and we managed to mitigate both patient and compartment batch effects (panel C). We believe that this is due to the effects on the T cell transcriptome of the tissue homogenization & digestion protocol needed to isolate intra-hepatic lymphocytes. This is despite having employed very stringent criteria to remove those intra-hepatic lymphocytes whose transcriptome showed signs of having been affected by the digestion protocol (19% of the total). Of note, we had much fewer difficulties in integrating scRNASeq data from PBMC and healthy liver tissue samples (presumably because these were paired samples), which is why we opted to describe the 2 analyses separately.

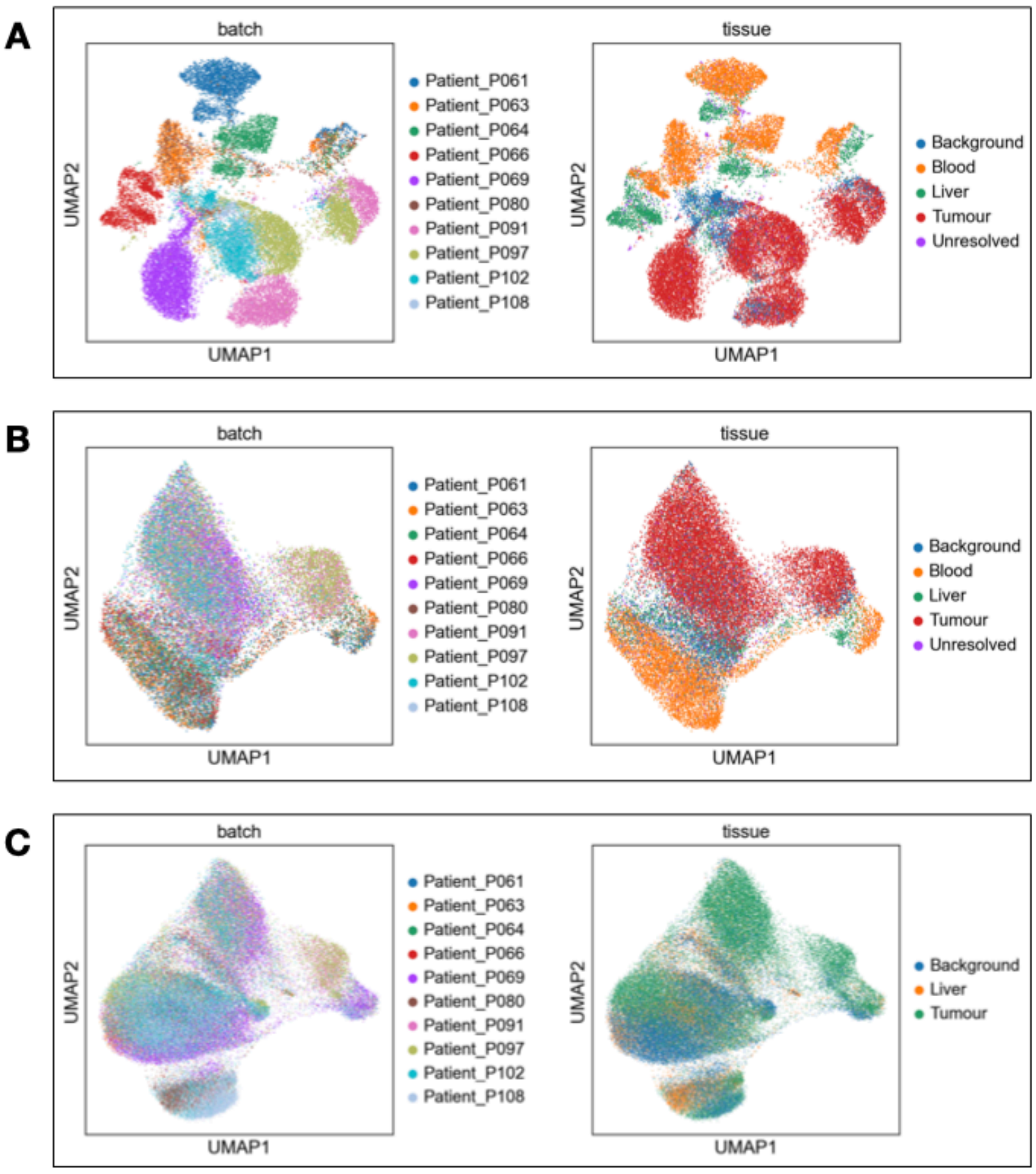

The methodology employed to integrate the different samples has been better described in the Supplementary Materials and Methods, and the figures shown above is now provided as **Fig.S23**.

As requested, the expression of key Treg genes is now provided to better illustrate the cluster annotation (see UMAPs in **Fig. S24**).

Finally, we agree with Reviewer 3 that scRNASeq data are highly descriptive and that any conclusions should be considered speculative in the absence of functional validation. As stated above, the objective of our project was not to investigate the origin of intra-tumoral Tregs (circulation versus surrounding non-tumoral tissue), given that previous studies in other type of cancers had already unambiguously settled this question (e.g. Plitas G et al. Immunity 2016). This is the reason why we did not analyse PBMC samples paired with the HCC samples. As outlined above, our objective was to elucidate which mechanisms allow Tregs to accumulate within the HCC lesions but not within the non-tumoral liver tissue. The scRNASeq data pointed us towards Nrf2 as a potential target, which we then validated conducting various *in vitro* and *in vivo* experiments. We have amended the manuscript to ensure that any claims based on scRNASeq data alone are appropriately tempered down.

7) *"Can the authors provide justification of the 64-gene signature for Cluster B' Tregs as it lacks typical Treg-feature genes like Foxp3?"*

To define a Cluster B'-specific gene signature likely to be of clinical relevance, we first ranked the genes differentially expressed between cluster B' and all other CD4+ T cells, next we selected those with FDR<0.01 and log2ratio>2 (179 genes), and finally we used Cox regression (LogRank p-value <0.05; Hazard Ratio >1) to identify those significantly associated with hepatocellular carcinoma (HCC) survival in the TCGA database (64 genes). This is described in page 8 of the Supplementary Materials (Supplementary Materials and Methods: "Transcriptomic analysis of publicly available scRNA-seq and bulk RNASeq HCC datasets").

8) *"The design of panel for Mass Cytometry in Fig. 5 is not clearly explained. How did the authors select those markers for analysis? The flow*

of the earlier discovery to Nrf2 seems rather sudden and without clear justification. Also, among other potential targets, picking Nrf2 for downstream analysis is rather strange and somewhat bias/cherry-picking".

The mass cytometry panel was adapted from a previously published study (Hartmann FJ et al. Nat Biotechnol 2021). Antibody concentrations and staining protocols were carefully optimized to reduce background and enhance signal intensity. To ensure specificity and consistency across batches, a control PBMC sample was included in every run, allowing validation of marker expression and dynamic range. This is now described in the Material & Methods section.

We apologize if the rationale for focusing on Nrf2 was not clearly described in the manuscript. We previously reported that in chronic liver disease (the pathology underlying most cases of HCC Tregs are dysfunctional and prone to apoptosis as a result of Nrf2 dysregulation (Vaikunthanathan T et al. Ebiomedicine 2023). We then hypothesized that the reverse would be true in HCC (i.e. that the HCC microenvironment would promote Treg accumulation by activating Nrf2, ensuring their metabolic fitness and survival). Addressing this hypothesis was one of the main objectives of our current study. Given that both our scRNASeq and CyTOF experimental supported this hypothesis, we decided to thoroughly investigate the functional effects of Nrf2 on intra-tumoral Tregs. We have amended our manuscript to ensure that our hypothesis and rationale is now clearly described (see Introduction [page 6], Results [page 12], and Study Design [page 24]).

9) *"The aim of introduction of H2O2 in Fig. 6 is not clear. How is that relevant to the earlier findings/conclusion?"*

We thank Reviewer 2 for seeking clarification on the rationale for investigating the effects of H_2O_2 (Fig.6). The HCC TME is characterized by elevated oxidative stress and increased reactive oxygen species (ROS) (Wang X et al. Scientific Reports 2024; Zhang S et al. Frontiers in Cell and Develop Biol 2023; Ma-On C et al. Medical Oncology 2017). This is driven by multiple factors such as hypoxia, inflammatory cell infiltration, and metabolic reprogramming (Chen TH et al. Cancer Metastasis Reviews 2025; Wang Z et al. Oxidative Medicine and Cellular Longevity 2016; Cardin R et al. World J of Gastroenterol 2014). We used H_2O_2 to investigate *in vitro* the influence of Nrf2 activation on the effects of oxidative stress on both Tregs and Tconv. This allowed us to demonstrate for the first time that the cytoprotective effects of Nrf2 activation are different for Tregs and Tconv (Nrf2 activation induced by DMF treatment preserved the viability of Tregs, but not Tconv, even when exposed to the highest concentration of H_2O_2; see **Fig.6B** and **C**). We believe this is an important finding that complements our additional experiments investigating the differential impact of Nrf2 activation on Tregs and Tconv exposed to high lactate/low glucose culture conditions (see **Fig.6D-K**). Taken together, what these experiments reveal is that in culture conditions that replicate the characteristics of the TME (i.e. oxidative stress induced by H2O2 and metabolic stress caused by high lactate/low glucose), Nrf2 provides survival advantages to Tregs, but not to Tconv.

10) *"The comprehensive TILs analysis e.g. effector (CD8+ T and NK cells) immune cell subsets on Treg-specific Nrf2 KD model is lacking. The similar analysis like those provided in Fig. 7I is important to explain the effect on tumour burden in this model".*

The flow cytometry experiments described in Fig. 7I were performed by our collaborator Dr Jack Leslie in Newcastle. Unfortunately, we could not perform the same type of analyses at the time we performed the Treg-specific Nrf2-knock-out experiments. What we conducted were IHC analyses, which, reassuringly, showed consistent results with changes in Tregs and in the CD8+/FoxP3+ (Treg) ratio (**Fig. 7E**). This suggests that both systemic and Treg-specific Nrf2 inhibition results in similar changes to the immune tumour microenvironment.

11) *"Histology analysis on mice fed with western diet should be provided to confirm MAFLD conditions in the liver"*

We apologize for this omission. Our collaborator Dr Jack Leslie, who supervised the HCC murine experiments included in our study, has already reported that western diet consistently results in MAFLD conditions (Leslie J et al. Gut 2022). The corresponding reference has been added to the amended manuscript. Furthermore, representative micrographs of liver tissue stained for histopathological evaluation and the MASH scores have been included as **Fig. S17** and **Table S15**.

Reviewer 3:

1) *"Authors show that Tregs in non-tumoral liver tissue undergo apoptosis. The liver is also a storage for iron. So, is it apoptosis or ferroptosis?"*

We agree with Reviewer 3 that distinguishing between apoptosis and ferroptosis is important, particularly given the liver's role as an iron storage organ and the emerging recognition of ferroptosis as a significant cell death pathway in liver pathology. Our data demonstrating increased Annexin V positivity in non-tumoral liver Tregs (**Fig. 1F**), which indicates phosphatidylserine externalization, is a feature common to multiple forms of programmed cell death and cannot definitively distinguish between apoptosis and ferroptosis. In order to unambiguously answer this question, the following experiments would have been required:

- Ferroptosis-specific markers (e.g., lipid peroxidation products, iron accumulation assays)
- Assessment of GPX4 expression and glutathione levels
- Treatment with ferroptosis inhibitors (ferrostatin-1) versus apoptosis inhibitors (z-VAD-FMK)
- Evaluation of characteristic morphological features by electron microscopy.

Unfortunately, the limited yield of Tregs obtained from surgical liver resections precluded us from conducting these experiments. We have described this as one of the limitations of our study. In the absence of these data, what our study can conclude is that tumour-infiltrating Tregs exhibit enhanced survival compared to their non-tumoral liver counterparts, with this cytoprotective effect being mediated through Nrf2-dependent signalling pathways. The enhanced survival phenotype is observed irrespective of the specific cell death mechanisms involved.

2) *"For me it is not clear by which mechanism Tregs behave different from T conv in HCC. Tregs are normally metabolically less active that Tconv. While HCC Tregs show an increased metabolic activity. So there has to be a fundamental switch. Can authors show why the MCT-1 lactate uptake is higer in Tregs? Is lactate inducing NRF2 by Keap1-Lactylation or is NRF2 inducing MCT-1? Can authors answer this by means of the NRF2-KO mice? Or at least discuss this point. It would also help to underpin the metabolic profiling of HCC Tregs by means of SCENITH assays. Also the role for NRF2 in mitochondrial regulation could by analyzed more in detail, to see if all or one complex of the electron transport chain is affected in NRF2 deficient mice and also in HCC Tregs. This can be analyzed by using the method of Erny et al. (STAR PROTOCOLS, 2022)".*

We appreciate Reviewer 3's observations and the need to further clarify the interpretation and discussion of our findings. The dependence of Tregs on MCT1-medidated lactate uptake to maintain their suppressive capacity within the TME was originally described by Delgoffe et (Watson MJ et al. Nature al 2021). What our findings now show is that lactate uptake via MCT1 induces lactoylation of Keap1, leading to Nrf2 stabilization and activation. We believe that this lactate-driven Nrf2-mediated metabolic reprogramming represents the fundamental switch that distinguishes HCC Tregs from both HCC Tconv and Tregs in non-tumoral tissues.

To address the directionality question, we have complemented our original experiments showing that MCT1 inhibition abrogates lactate-dependent Nrf2 activation, with new experiments using siRNA that demonstrate that Nrf2 knockdown in human Tregs does not impact MCT1 expression (see figure below). These findings establish the pathway as: Lactate uptake (MCT1) → Keap1 lactoylation → Nrf2 activation. These results have been incorporated as **Fig. S12**, and the Supplementary Materials and Methods section has been updated to include the methodological details of the siRNA experiment.

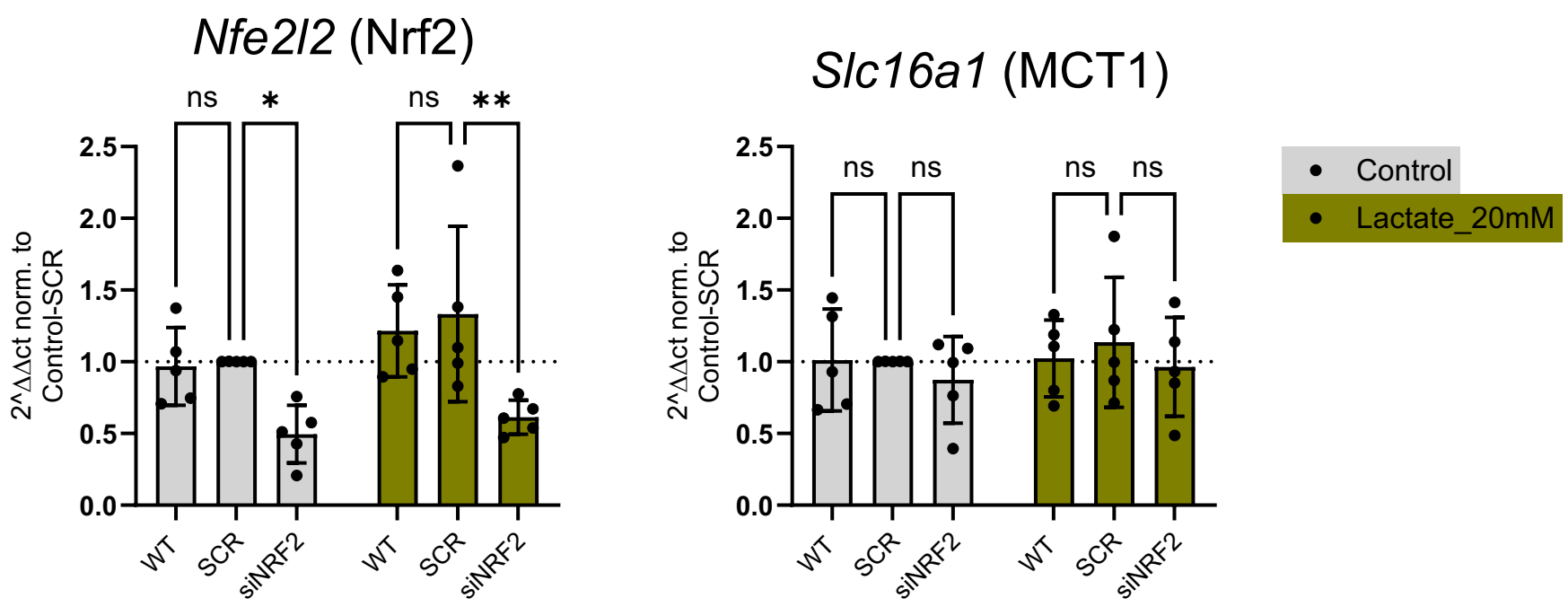

Nrf2 knockdown does not alter MCT1 expression in human Tregs. Relative mRNA expression levels of *Nfe2l2* (Nrf2) and *Slc16a1* (MCT1) in human Tregs, normalized to control. Tregs were freshly isolated using magnetic beads and expanded for 7 days with anti-CD3/CD28 beads (bead-to-cell ratio 1:1), rapamycin (100 nM), and recombinant human IL-2 (50 U/mL). Cells were then either left untransfected (wild-type, WT), transfected with a control plasmid (SCR), or with siRNA targeting Nfe2l2 (siNRF2). After 48 hours of culture in standard RPMI medium with or without 20 mM lactate, qPCR was performed to assess gene expression.

Importantly, our results reveal that the survival advantage provided by Nrf2 is specific to Tregs. While CD4 and CD8 effector T cells are not dependent on Nrf2 for viability in lactate-enriched environments, Tregs from global Nrf2 KO mice showed compromised survival and were more prone to apoptosis under tumour-mimicking conditions (**Fig.6J** and **K**). This selectivity suggests that Tregs have evolved a unique dependency on Nrf2-mediated stress responses for TME adaptation. Our findings provide a clear rationale for targeting the lactate-MCT1-Nrf2 axis in HCC Tregs. The cell-type specificity of Nrf2 dependence suggests that therapeutic intervention could selectively compromise tumour-infiltrating Tregs while preserving effector T cell function.

We agree with Reviewer 3 that the SCENITH assay provides valuable metabolic readouts. However, given the small number of lymphocytes available for ex vivo analyses, we decided to conduct instead a combination of scRNASeq and CyTOF analyses. We believe that these multi-dimensional approaches provide a more complete picture of the metabolic reprogramming than the two primary readouts measured by SCENITH.

3) *"In figure 1. the gating strategy is not clear to me. authors should describe in detail how they gate on Tregs and are sure not to include T conv the Treg gates. The sorting strategy also needs to be clarified".*

We apologize for not having provided this information. The gating strategy used to identify human Tregs is shown in **Fig. S1**. Additionally, **Fig. S21** illustrates the gating strategy applied during cell sorting prior to scRNA-seq.

4) *"Fig. 1 A-E the lymph node data are from 3 donors and show high variation. This should be interpreted with caution".*

Reviewer 3 is totally right in pointing this out. We decided to include the data from a limited number of intra-abdominal lymph node samples to complement the observations obtained in spleen samples. The fact that both types of secondary lymphoid tissues show similar results is reassuring. However, to ensure that we do not over-interpret our results, we have eliminated from the manuscript any mention or claim based on the lymph node results.

5) *"NRF2 activation should in addition to flow cytometric analysis of NRF2p also be assessed by WB of NRF2 or its target gene NQO1"*

We agree with Reviewer 3 on the need to provide additional proofs of Nrf2 activation in addition to the increased Nrf2p levels shown in **Fig.5**. We have provided such supporting information by analysing our scRNASeq dataset using a signature that incorporates the key Nrf2 targets (among them *NQO1*). As shown in **Fig.S9B** and **C**, the transcriptomes of the most metabolically active intra-tumoral Treg clusters (B' and E') were the most significantly enriched in this Nrf2 target signature. Unfortunately, we were not able to isolate sufficient Tregs to conduct confirmatory WB experiments.

6) *"Fig.6: Were these experiments only performed with 3 mice in each group? Please use at least 5 mice when you want to show clear differences".*

As per Reviewer 3's suggestion, we have now conducted additional independent experiments to increase the sample size and strengthen the robustness of our results. Reassuringly, the results of these experiments (depicted in the amended version of **Fig.6**) confirm our original observations.

7) *"6A: Why did authors gate on HO-1 here and not NQO1, which is kwon to be the high specific NRF2 target gene".*

The results shown in **Fig.6A** correspond to an *in vitro* experiment that employed flow cytometry as read-out. Given the lack of reliable flow cytometry antibodies targeting NQO1, we used HO-1 as a surrogate to identify Nrf2 activation.

8) *"Discussion:*
-There is a study from Maj et al.(Nat. Immun. 2017) which in contrast to this study showed that tumor Tregs are more vulnerable for apoptosis and have a weak anti-oxidant system. Authors should discuss this study and the differences to their findings.
- authors should mention limitations of the study
- authors state that it is benefecial to target NRF2 especially in inflammed HCC. While this might be true for established tumors, authors should also mention that targeting Tregs and or NRF2 can also have dramatic effects in inflammation, such as NASH ".

We thank Reviewer 3 for raising these points. The manuscript's Discussion section now includes a brief commentary on the routes towards clinical translation as well as the potential challenge of treating patients with MASLD. Furthermore, we have added paragraph describing the limitations of the study as well as the contrasting results described by Maj. et al.

We would like to thank you for your very insightful comments. We believe that the quality and scientific robustness of our manuscript has been significantly improved as a result of the review process. Of note, we have now included an additional dataset showing that patients with advanced HCCs enriched in Tregs with high Nrf2 activation exhibit shorter progression-free survival following Atezolizumab/Bevacizumab treatment (see **Fig.4**). These novel data substantially increase the clinical relevance of our findings.
Altogether, we hope that our manuscript now meets the standards for publication in Nature Communications.

Thank you all again.

With best wishes,

Dr N Safinia BSc (Hons), PhD, FRCP
Medical Research Council (MRC) Clinician Scientist
Roger Williams Institute of Liver Studies Hepatobiliary Cancer Theme Lead
School of Immunology and Microbial Sciences
King's College London University
London SE5 9NU, United Kingdom

Professor Sanchez Fueyo MD PhD
Professor of Hepatology
Academic Deputy Director of Roger Williams Institute of Liver Studies
School of Immunology and Microbial Sciences, King's College London University London SE5 9NU, United Kingdom

Dear Reviewers,

We are grateful for the opportunity to provide additional information in support of our submission.

As requested by Reviewer #2, we have appended below a new version of the original point-by-point letter including all revised/new data and addressing the additional issues raised (see point 4). We apologise for the mislabelling of some of the Figures, which we have now corrected.

Response to Reviewer #2

1) *"Your study identifies Nrf2 as a critical regulator of intratumoral Treg survival and function in HCC, suggesting its inhibition as a therapeutic strategy. However, prior studies have shown context-dependent roles of Nrf2 in Tregs. For instance, Klemm et al. (EJI, 2019) reported that constitutive Nrf2 activation via Keap1 deletion destabilizes Tregs and leads to autoimmunity, while a more recent study (Front. Immunol., 2024) showed that pharmacological Nrf2 activation by dimethyl fumarate enhances Treg differentiation and function in allergic asthma. Given these contrasting outcomes, could the authors speculate on how the TME-specific metabolic context (e.g., lactate-induced oxidative stress) uniquely shapes Nrf2 function in Tregs in HCC, and how this might differ from homeostatic or inflammatory settings such as allergy? Additionally, how do the authors envision the therapeutic window for Nrf2 inhibition, given its potentially beneficial role in peripheral immune tolerance?".*

We thank Reviewer 2 for this insightful question regarding the context-dependent roles of Nrf2 in Tregs. As hinted by the reviewer, we believe that the apparent contradictions in the literature can be reconciled by considering the unique metabolic and oxidative stress conditions within the tumour microenvironment (TME) that distinguish it from homeostatic or alternative inflammatory settings. Our data indicate that one of the candidate metabolites, lactate, which is found at high concentration within the TME (Kim EY et al. Molecules (Basel) 2025; Barba I et al. Int J of Mol Sci 2024; Pérez-Tomás R et al. Cancers 2020; Jin M et al. Frontiers in Cell and Develop Biology 2022; Gao Y et al. J of Immunol Res 2022; Tan S et al. Int J of Mol Sci 2025; Romero-Garcia S et al. Frontiers in Immunol 2016) induces lactoylation of Keap1, leading to Nrf2 stabilization and activation. This post-translational modification represents a metabolic adaptation mechanism that is specific to the lactate-rich TME and that differs fundamentally from both the genetic perturbations studied by Klemm et al. and the pharmacological activation examined in allergic asthma models. We hypothesize that the key distinction lies in the stimulus-response context: constitutive genetic activation through Keap1 deletion creates sustained, supraphysiological, Nrf2 activation that disrupts normal cellular homeostasis, while TME-induced lactoylation provides a proportionate, stress-responsive, activation that maintains Treg viability under hostile conditions. Similarly, pharmacological Nrf2 activation in allergic asthma operates in a different metabolic context, in which glucose availability and lactate concentrations differ markedly from what is found in the TME.

Regarding the therapeutic window for Nrf2 inhibition, we acknowledge the potential risks of systemic inhibition, given Nrf2's key anti-oxidant function and the role in peripheral immune tolerance that our work has elucidated. However, several emerging strategies could provide tumour-selective targeting, such as: 1) the use of Nrf2 inhibitors designed as inactive compounds that are selectively activated within the TME (e.g. in response to the altered pH conditions present in tumours); 2) Nrf2 inhibitors could be administered directly into the HCC tumours (e.g. through transarterial chemoembolization (TACE), which is routinely employed as treatment for HCC); 3) the use of proteolysis-targeting chimeras (PROTACs) designed for tumour-selective degradation of Nrf2 (Ji J et al. J of Med Chem 2023).

We have provided clarifications on these topics in the amended version of the manuscript's Discussion.

2) *"Specific for Fig. 1H, given the differences in Treg shown in TILs vs NILs and PBMCs, can the authors explain why the Treg in TILs is only significantly more immunosuppressive vs those in NILs but not PBMCs? In addition, It would also be helpful to discuss the potential mechanisms driving the differences in Tregs phenotypes, particularly the role of IL-2 availability in more detail e.g. by providing analysis of IL-2 level to support the speculation".*

We thank Reviewer 2 for this comment. While it is true that no statistically significant differences were observed between TIL and PBMC Tregs, **Fig. 1i** shows a strong trend in favour of TIL Tregs (as compared to both PBMC and healthy liver Tregs). Furthermore, *in vitro* suppression assays are extremely reductionistic in that they do not capture the whole array of immunosuppressive mechanisms exerted by Tregs. Indeed, the higher activation status and expression levels of phenotypic markers such as CTLA-4, TIGIT, CD95 and PD-1 noted on TIL Tregs indicate that these cells are likely to be substantially more immunosuppressive than those detected in other tissue compartments.
In response to Reviewer 2's request we have conducted additional RNA-seq and cytokine array experiments to better characterize the inflammatory environment of tumoral and non-tumoral liver tissue and identify potential drivers of HCC TIL Treg accumulation/fitness. These experiments (provided as **Supplementary Fig. 10**) have revealed no differences between the 2 tissue compartments in what regards conventional Treg growth factors, which further support our conclusion that Treg metabolic adaptations are key for their expansion within the tumour microenvironment.

Fig. 1i:

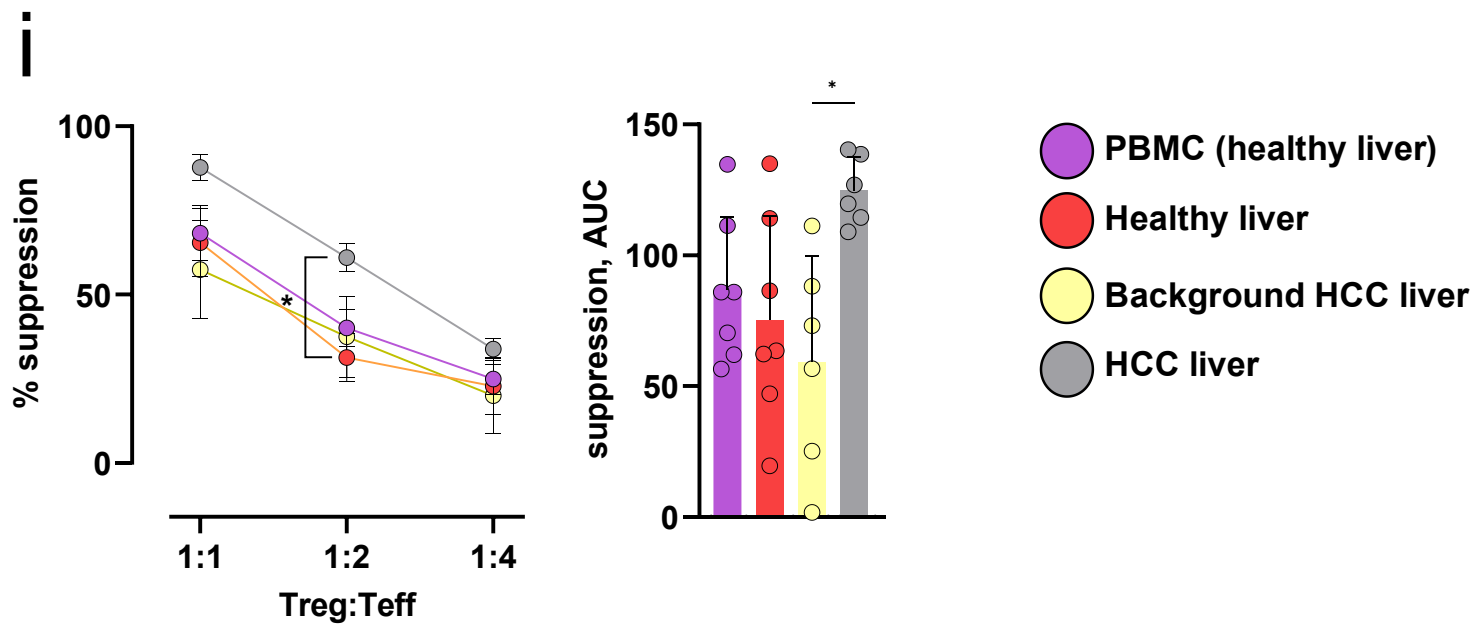

Supplementary Fig. 10:

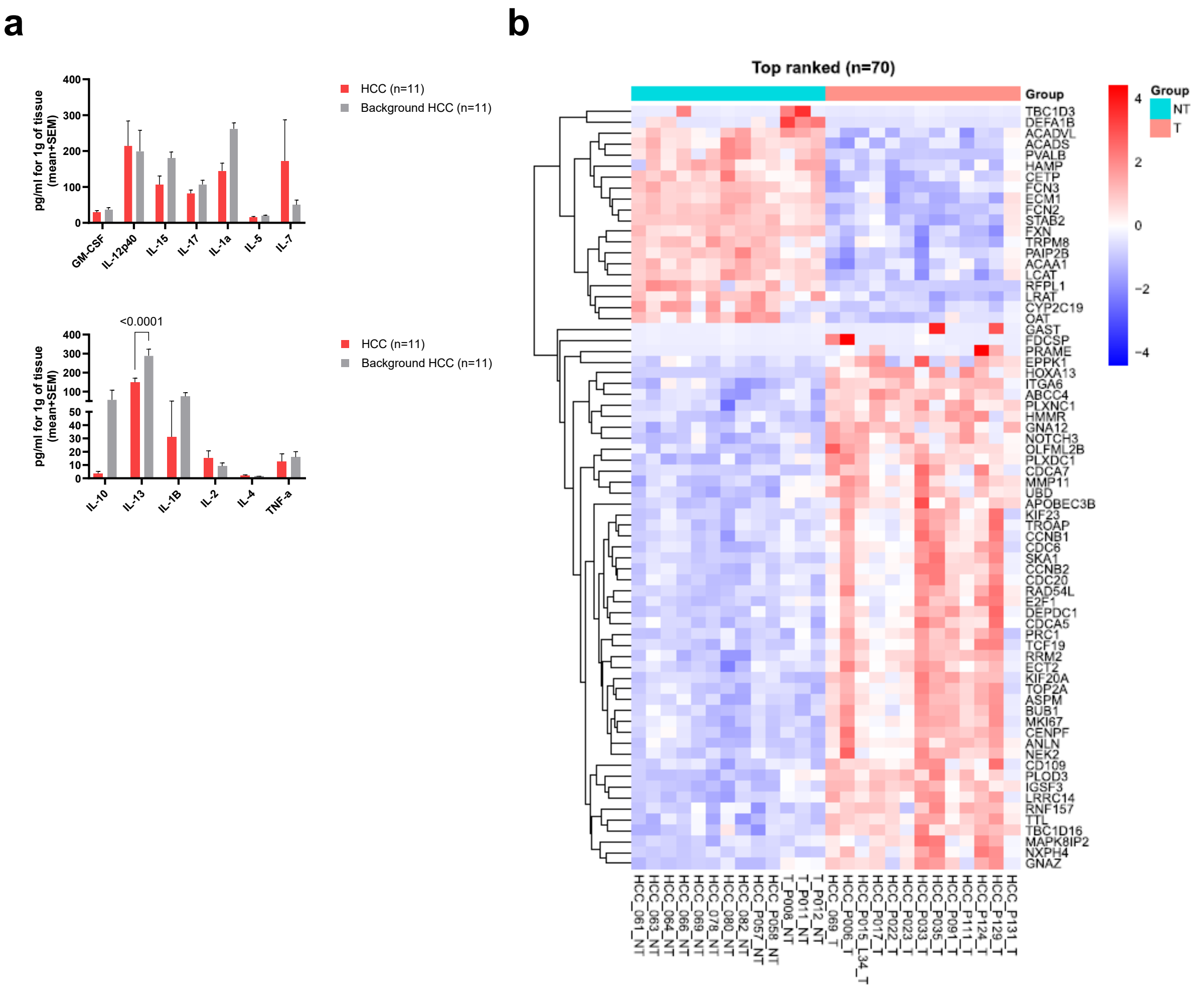

3) *"For the scRNA seq data, if the focus on this study is to decipher phenotypes/ function of Tregs in TILs, why is the scRNA seq analysis conducted only on blood and paired healthy livers but not HCC tumours? Also, it is not clear how Clusters 3, 8, 11, 13 and 15 corresponded to Tregs, the authors should provide expression of key Treg genes, particularly Foxp3, in UMAP or Dot Plot to confirm the annotation".*

The objectives of our study were to identify the mechanisms through which Tregs adapt to the liver microenvironment and determine whether/how these mechanisms are modified once liver cancer arises (see Introduction [page 5] and Study Design [page 23] sections). This is the reason why we first compared blood and paired healthy liver, and only afterwards analysed the differences between cancerous and non-cancerous liver tissue.

As requested, we now provide dot plots showing the expression of *FOXP3* and other key genes to confirm the identity of the Treg clusters (see **Supplementary Fig. 25**).

Supplementary Fig. 25:

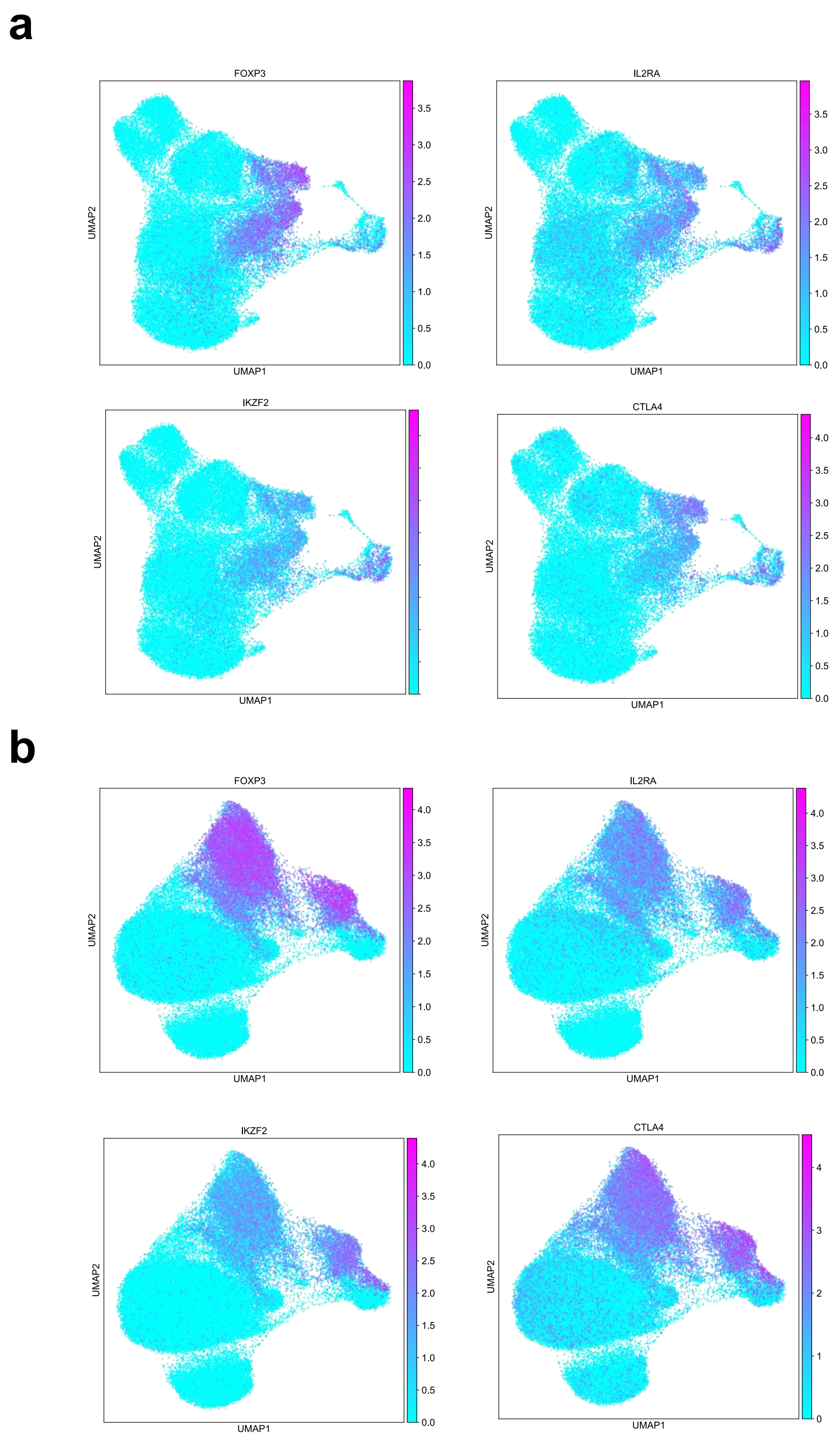

4) *"The scRNA seq analysis related to Fig. 2 is largely descriptive and speculative. For instance it is not clear of the implications of these differences based on genes enrichment or TCR repertoire analysis. For instance, TCR overlap analysis is supportive but not definitive in speculation on Treg trajectory. In fact, a trajectory analysis will be more helpful in this conclusion. Second, the pseudo-bulk approach is acceptable, but a more granular analysis (e.g., module scoring across clusters or pathway-level analysis per Treg subset) would provide greater resolution.*

We are grateful for Reviewer 2's comments. As suggested, we have now conducted pseudotime trajectory analyses employing the *scFates* package to strengthen the conclusions of our original TCR repertoire overlap analyses. The results of these new analyses (provided as **Supplementary Fig. 7**) confirm that clusters E and G (liver) constitute different cell states that derive from cluster C (blood), with cluster E being in closer continuity with circulating Tregs than cluster G. We believe these results

support our hypothesis that circulating Tregs face 2 different fates when they reach the liver: they either become metabolically quiescent and prone to apoptosis (cluster E) or they become metabolically active and clonally expand (cluster G), presumably as a result of being able to recognize cognate antigens.

Supplementary Fig. 7:

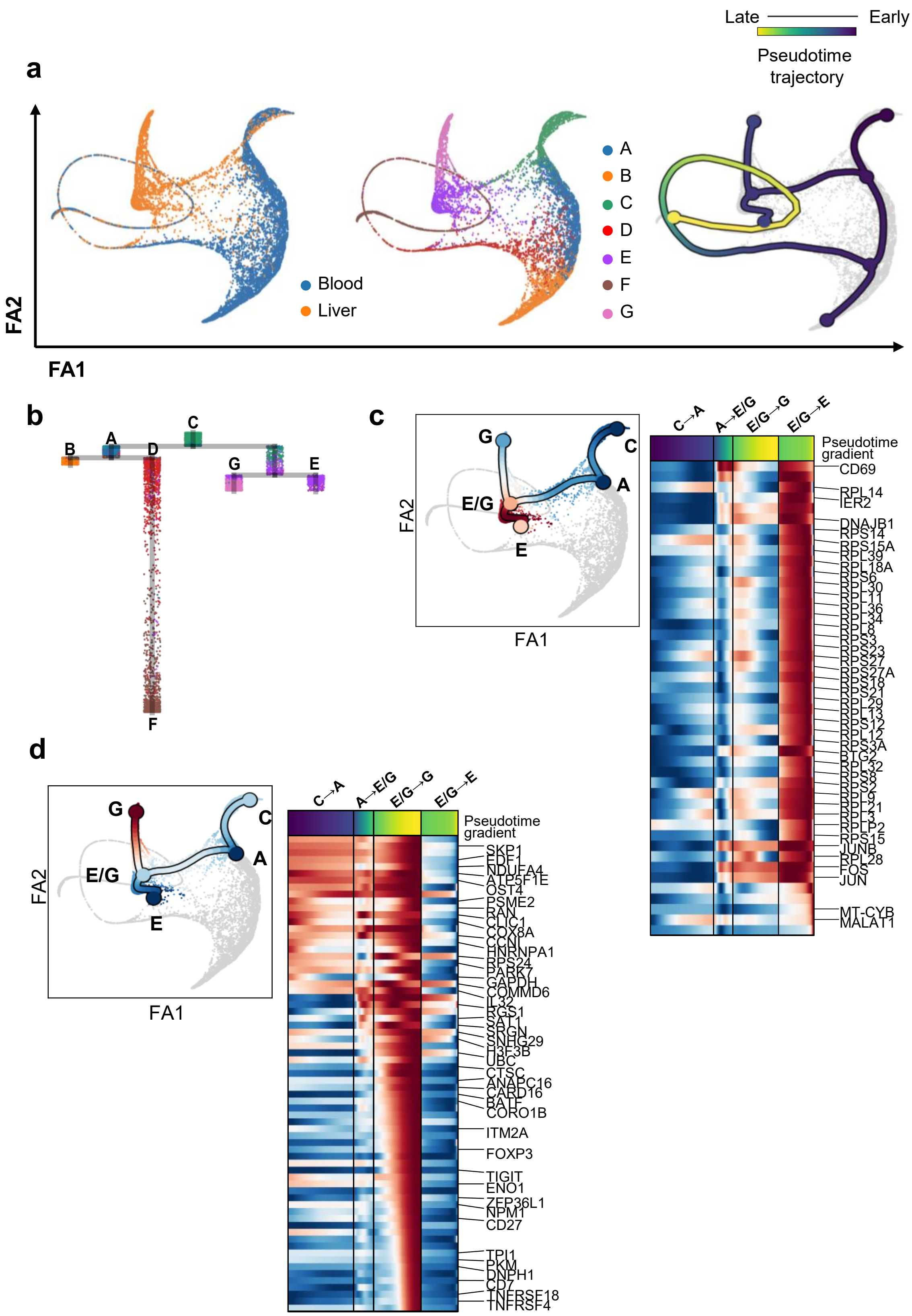

“The trajectory analysis plot seems unconventional, and the directionality of the trajectory is unclear. Furthermore, the gene expression or pathway differences leading to the two distinct fates (E vs. G) are not analysed or illustrated. Please clarify the trajectory direction and include data or discussion that elucidates the molecular or pathway distinctions between these two fates”.

To assess dynamic trajectories, we have employed *scFates* v1.1.1 , a well-established Python package that uses principal graph learning (a conventional technique to conduct trajectory analyses). *scFates* is part of the *scverse* ecosystem and therefore compatible with other Python packages (e.g. Harmony and Scanpy) that we have employed in our scRNASeq data analysis pipeline (see https://scfates.readthedocs.io/en/latest/Basic_Curved_trajectory_analysis.html).

The graphs shown in **Supplementary Fig. 7** correspond to a linear (curve) trajectory inference from the single cell data with cluster C as the root or starting point. The directionality can be inferred from the colour scale provided in panel A and from the dendrogram shown in panel B. As requested, we have now analysed the transcripts linked to the bifurcation between clusters E and G, and we provide the data in panels C and D of the amended version of the **Supplementary Fig. 7**.

5) *"Most importantly, the narrative is somewhat confusing: the initial observations focus on TIL-Tregs, but the subsequent mechanistic analyses (e.g., scRNA-seq) shift to intrahepatic Tregs from healthy liver tissue, without addressing TIL-Tregs in the same depth (same comment as in #3). Furthermore, the added value of subclustering Treg from Tconv, which is already a highly specific subset of CD4⁺ T cells, is not clear.*

The narrative of the manuscript follows the objectives of the study, which were: 1) to identify the key determinants of Treg liver tissue residency; 2) to investigate how these processes are impacted by HCC development; and 3) to elucidate the contribution of Nrf2 signalling to the homeostasis of intra-tumoral Tregs and to the progression of HCC. This is the reason why, after describing the differential accumulation of Tregs in non-tumoral and tumoral liver tissue as compared to other compartments, our scRNA-seq experiments were first focused on the differences between Tregs residing on healthy liver and those found in the circulation. Having determined that liver tissue Tregs exhibit some unique features, we investigated the extent to which these features change when primary liver cancer develops. We apologise if this did not come across clearly enough. We have amended the manuscript to highlight the study's objectives and we have provided more clarity as to the narrative (see Introduction [page 5], Results [page 11], and Study Design [page 23] sections).

We decided to re-cluster the cells that we had identified as Tregs to ensure that the resulting Treg clusters would be driven by differences in the Treg transcriptome, rather than by major transcriptional differences between Tregs and Tconv (which is what accounts for the differences observed in the UMAPs shown in **Supplementary Fig. 3a** and **Supplementary Fig. 8a**). We believe this is standard practice in the scRNA-seq field, although we agree that it increases the complexity of the results. We have carefully reviewed our manuscript and added additional clarifications were needed to facilitate the reading.

Supplementary Fig. 3a:

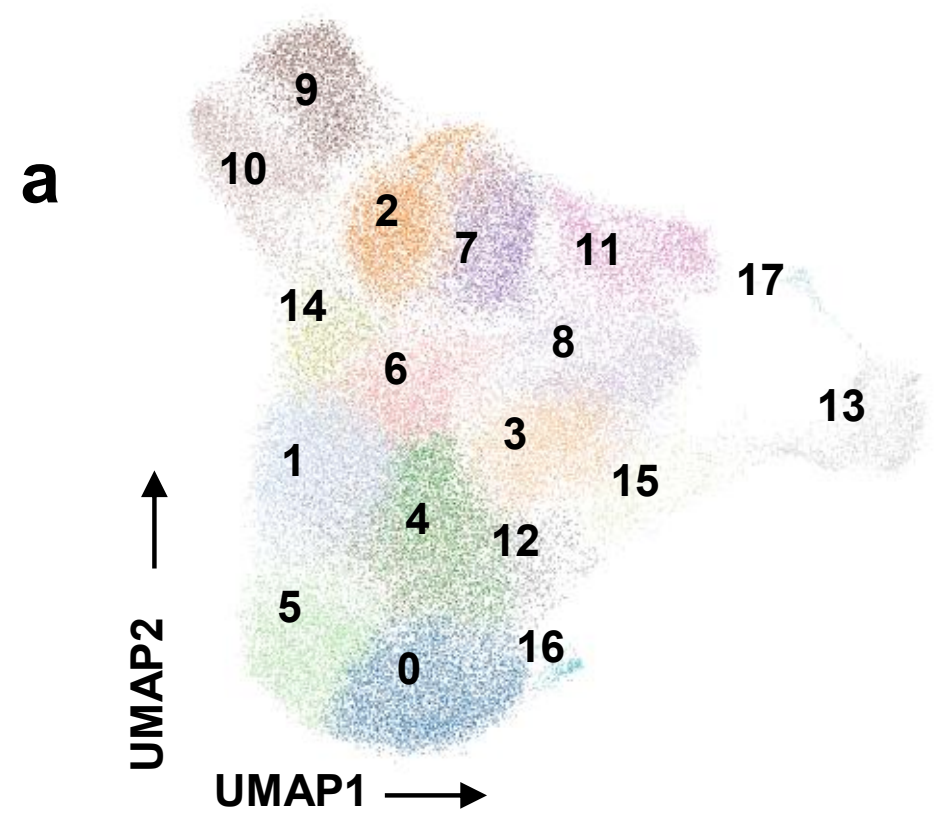

Supplementary. Fig. 8a:

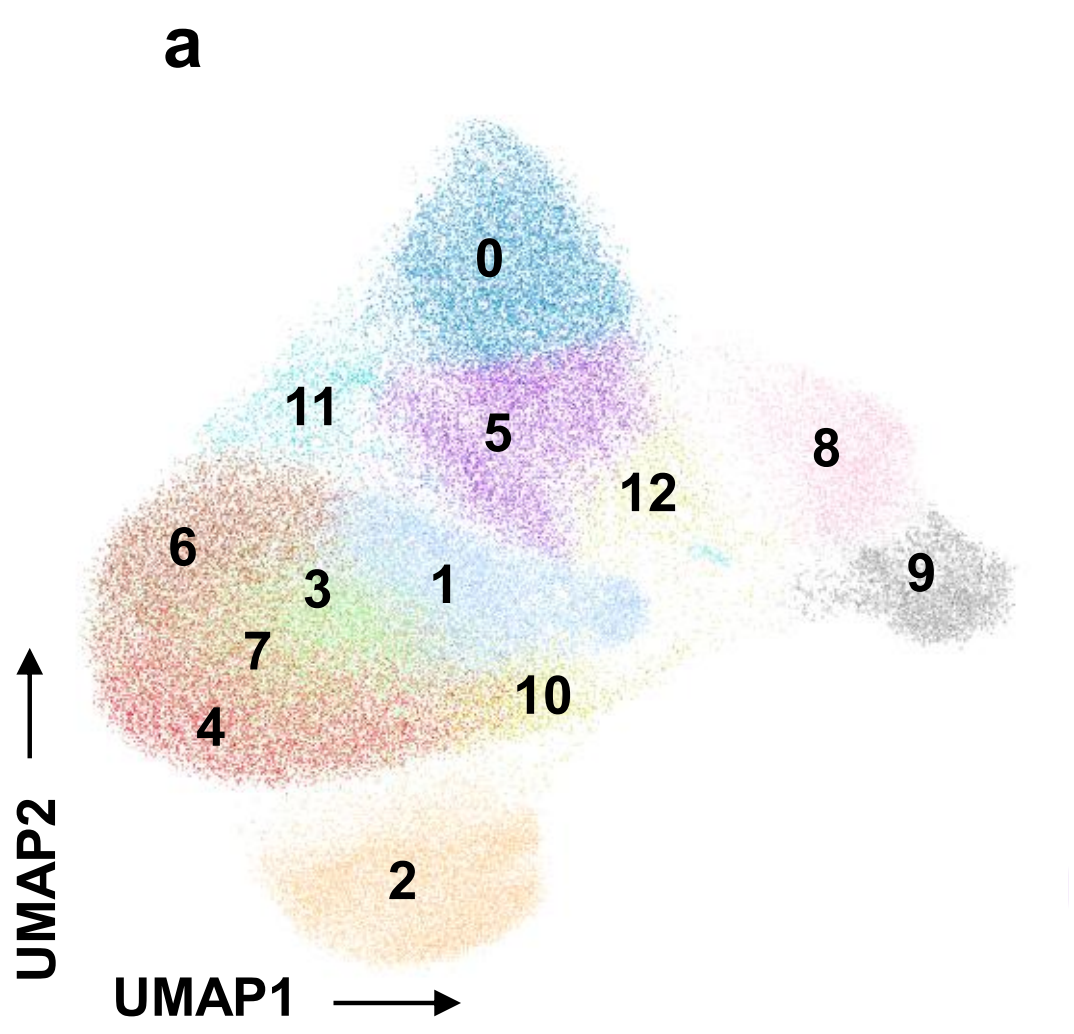

6) *"Finally the analysis based on TILs-Treg is provided in Fig.3. In my opinion, Integrating all three compartments: TILs, NILs and PBMCs in a unified analysis (instead of providing redundant analysis in Fig. 2 & 3) would have provided a more comprehensive and physiologically relevant understanding of Treg heterogeneity and dynamics in HCC. In addition, same issues as comment in #3, the authors should provide expression of key Treg genes in UMAP or Dot Plot to justify the clusters annotation. Furthermore, this analysis is again highly speculative without any functional evidence. The interpretation that TIL Tregs are oligoclonally expanded and distinct from non-tumoral liver Tregs is also based on TCR overlap but lacks depth in clonal trajectory analysis".*

We agree with Reviewer 2 that, rather than conducting 2 separate sets of scRNA-seq analyses, it would have been more informative to integrate all tissue compartments (i.e. blood, non-tumoral liver tissue, tumoral liver tissue) into one single analysis. We did conduct an analysis of all compartments grouped together (i.e. PBMC, healthy liver, background HCC liver, and HCC), but had to discard it because the PBMC compartment hindered the overall data integration and prevented us from simultaneously dealing with the heterogeneity of patients and compartments.

As can be seen in panel A of the figure below (which includes all samples pooled together), an integration step was necessary to mitigate the batch effects from the different patients. When we attempted to remove the patient batch effect on the whole dataset, most tissue compartments remained separated (panel B), which would have interfered with any subsequent analyses (i.e. most Treg clusters would have been compartment-specific). In contrast, when we removed the PBMC compartment, data integration was much more successful, and we managed to mitigate both patient and compartment batch effects (panel C). We believe that this is due to the effects on the T cell transcriptome of the tissue homogenization & digestion protocol needed to isolate intra-hepatic lymphocytes. This is despite having employed very stringent criteria to remove those intra-hepatic lymphocytes whose transcriptome showed signs of having been affected by the digestion protocol (19% of the total). Of note, we had much fewer difficulties in integrating scRNA-seq data from PBMC and healthy liver tissue samples (presumably because these were paired samples), which is why we opted to describe the 2 analyses separately.

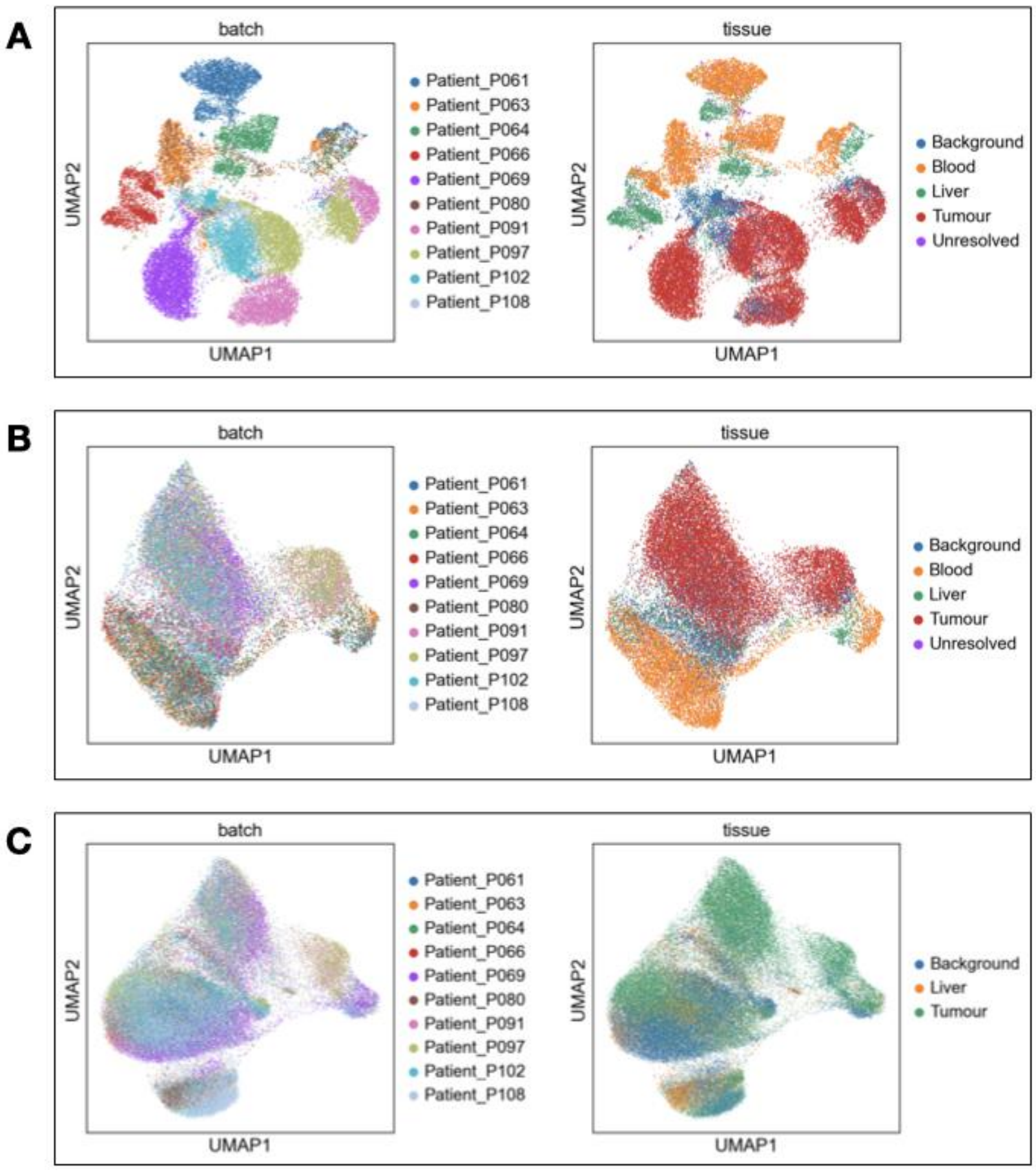

The methodology employed to integrate the different samples has been better described in the Supplementary Materials and Methods, and the figures shown above is now provided as **Supplementary Fig. 24**.

As requested, the expression of key Treg genes is now provided to better illustrate the cluster annotation (see UMAPs in **Supplementary Fig. 25**).

Finally, we agree with Reviewer 3 that scRNA-seq data are highly descriptive and that any conclusions should be considered speculative in the absence of functional validation. As stated above, the objective of our project was not to investigate the origin of intra-tumoral Tregs (circulation versus surrounding non-tumoral tissue), given that previous studies in other type of cancers had already unambiguously settled this question (e.g. Plitas G et al. Immunity 2016). This is the reason why we did not analyse PBMC samples paired with the HCC samples. As outlined above, our objective was to elucidate which mechanisms allow Tregs to accumulate within the HCC lesions but not within the non-tumoral liver tissue. The scRNA-seq data pointed us towards Nrf2 as a potential target, which we then validated conducting various *in vitro* and *in vivo* experiments. We have amended the manuscript to ensure that any claims based on scRNA-seq data alone are appropriately tempered down.

7) *"Can the authors provide justification of the 64-gene signature for Cluster B' Tregs as it lacks typical Treg-feature genes like Foxp3?"*

To define a Cluster B'-specific gene signature likely to be of clinical relevance, we first ranked the genes differentially expressed between cluster B' and all other CD4+ T cells, next we selected those with FDR<0.01 and log2ratio>2 (179 genes), and finally we used Cox regression (LogRank p-value <0.05; Hazard Ratio >1) to identify those significantly associated with hepatocellular carcinoma (HCC) survival in the TCGA database (64 genes). This is described in page 8 of the Supplementary Materials (Supplementary Materials and Methods: "Transcriptomic analysis of publicly available scRNA-seq and bulk RNASeq HCC datasets").

8) *"The design of panel for Mass Cytometry in Fig. 5 is not clearly explained. How did the authors select those markers for analysis? The flow of the earlier discovery to Nrf2 seems rather sudden and without clear justification. Also, among other potential targets, picking Nrf2 for downstream analysis is rather strange and somewhat bias/cherry-picking".*

The mass cytometry panel was adapted from a previously published study (Hartmann FJ et al. Nat Biotechnol 2021). Antibody concentrations and staining protocols were carefully optimized to reduce background and enhance signal intensity. To ensure specificity and consistency across batches, a control PBMC sample was included in every run, allowing validation of marker expression and dynamic range. This is now described in the Material & Methods section.

We apologize if the rationale for focusing on Nrf2 was not clearly described in the manuscript. We previously reported that in chronic liver disease (the pathology underlying most cases of HCC Tregs are dysfunctional and prone to apoptosis as a result of Nrf2 dysregulation (Vaikunthanathan T et al. Ebiomedicine 2023). We then hypothesized that the reverse would be true in HCC (i.e. that the HCC microenvironment would promote Treg accumulation by activating Nrf2, ensuring their metabolic fitness and survival). Addressing this hypothesis was one of the main objectives of our current study. Given that both our scRNA-seq and CyTOF experimental supported this hypothesis, we decided to thoroughly investigate the functional effects of Nrf2 on intra-tumoral Tregs. We have amended our manuscript to ensure that our hypothesis and rationale is now clearly described (see Introduction [page 5], Results [page 11], and Study Design [page 23]).

9) *"The aim of introduction of H2O2 in Fig. 6 is not clear. How is that relevant to the earlier findings/conclusion?"*

We thank Reviewer 2 for seeking clarification on the rationale for investigating the effects of H_2O_2 (Fig.6). The HCC TME is characterized by elevated oxidative stress and increased reactive oxygen species (ROS) (Wang X et al. Scientific Reports 2024; Zhang S et al. Frontiers in Cell and Develop Biol 2023; Ma-On C et al. Medical Oncology 2017). This is driven by multiple factors such as hypoxia, inflammatory cell infiltration, and metabolic reprogramming (Chen TH et al. Cancer Metastasis Reviews 2025; Wang Z et al. Oxidative Medicine and Cellular Longevity 2016; Cardin R et al. World J of Gastroenterol 2014). We used H_2O_2 to investigate *in vitro* the influence of Nrf2 activation on the effects of oxidative stress on both Tregs and Tconv. This allowed us to demonstrate for the first time that the cytoprotective effects of Nrf2 activation are different for Tregs and Tconv (Nrf2 activation induced by DMF treatment preserved the viability of Tregs, but not Tconv, even when exposed to the highest concentration of H_2O_2; see **Fig.6b** and **c**). We believe this is an important finding that complements our additional experiments investigating the differential impact of Nrf2 activation on Tregs and Tconv exposed to high lactate/low glucose culture conditions (see **Fig.6d-k**). Taken together, what these experiments reveal is that in culture conditions that replicate the characteristics of the TME (i.e. oxidative stress induced by H2O2 and metabolic stress caused by high lactate/low glucose), Nrf2 provides survival advantages to Tregs, but not to Tconv.

Fig. 6

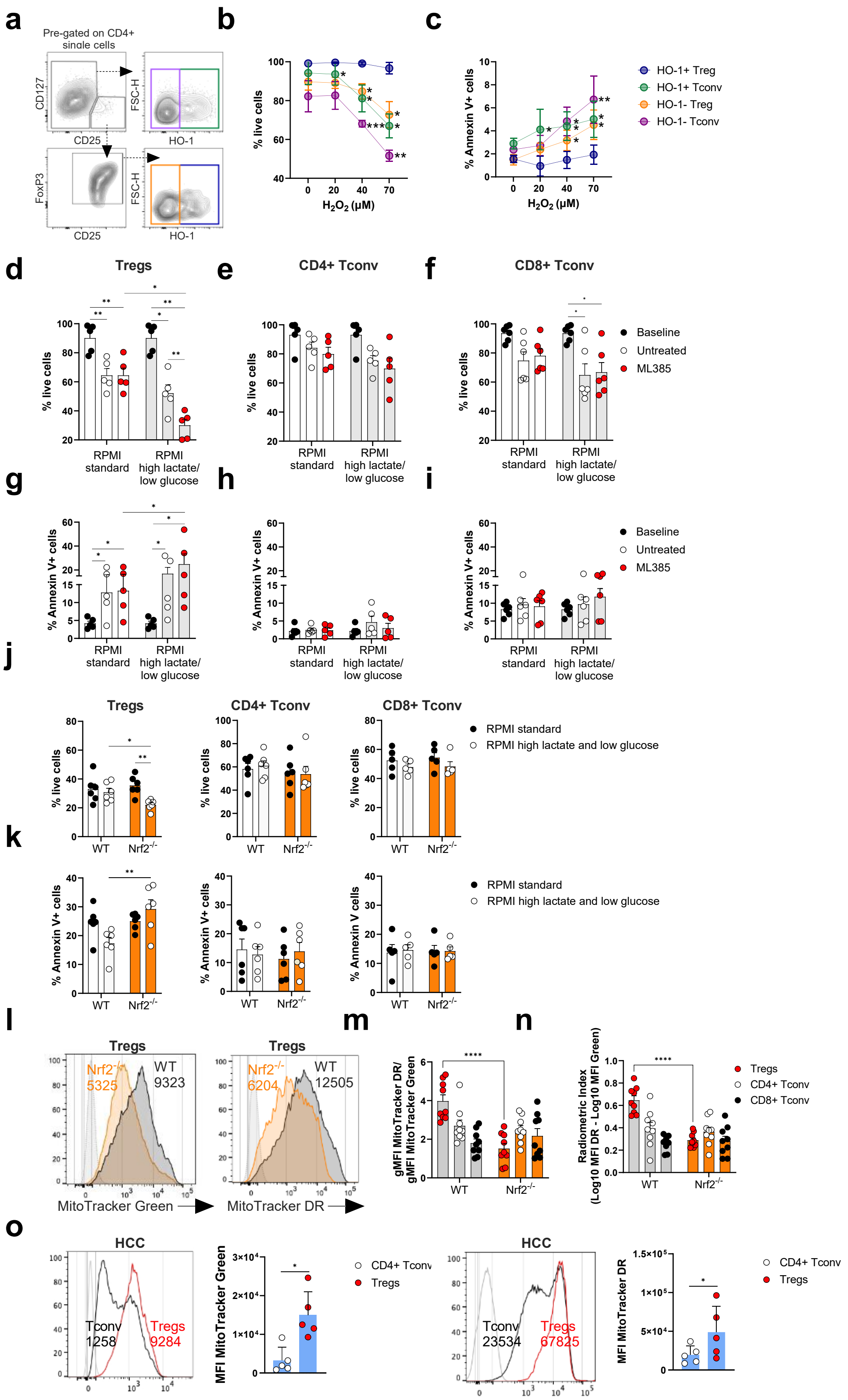

a
Pre-gated on CD4+ single cells
CD127
CD25
FSC-H
HO-1
FoxP3
b
% live cells
H2O2 (µM)
c
% Annexin V+ cells
H2O2 (µM)
HO-1+ Treg
HO-1+ Tconv
HO-1- Treg
HO-1- Tconv
d
Tregs
e
CD4+ Tconv
f
CD8+ Tconv
% live cells
RPMI standard
RPMI high lactate/ low glucose
Baseline
Untreated
ML385
g
h
i
% Annexin V+ cells
j
Tregs
CD4+ Tconv
CD8+ Tconv
% live cells
WT
Nrf2-/-
RPMI standard
RPMI high lactate and low glucose
k
% Annexin V+ cells
% Annexin V cells
l
Tregs
Nrf2-/- 5325
WT 9323
Nrf2-/- 6204
WT 12505
MitoTracker Green
MitoTracker DR
m
gMFI MitoTracker DR/ gMFI MitoTracker Green
n
Radiometric Index (Log10 MFI DR - Log10 MFI Green)
Tregs
CD4+ Tconv
CD8+ Tconv
o
HCC
Tconv 1258
Tregs 9284
MFI MitoTracker Green
CD4+ Tconv
Tregs
Tconv 23534
Tregs 67825
MFI MitoTracker DR

10) *"The comprehensive TILs analysis e.g. effector (CD8+ T and NK cells) immune cell subsets on Treg-specific Nrf2 KD model is lacking. The similar analysis like those provided in Fig. 7I is important to explain the effect on tumour burden in this model".*

The flow cytometry experiments described in **Fig. 7i** were performed by our collaborator Dr Jack Leslie in Newcastle. Unfortunately, we could not perform the same type of analyses at the time we performed the Treg-specific Nrf2-knock-out experiments. What we conducted were IHC analyses, which, reassuringly, showed consistent results with changes in Tregs and in the CD8+/FoxP3+ (Treg) ratio (**Fig. 7e**). This suggests that both systemic and Treg-specific Nrf2 inhibition results in similar changes to the immune tumour microenvironment.

Fig. 7:

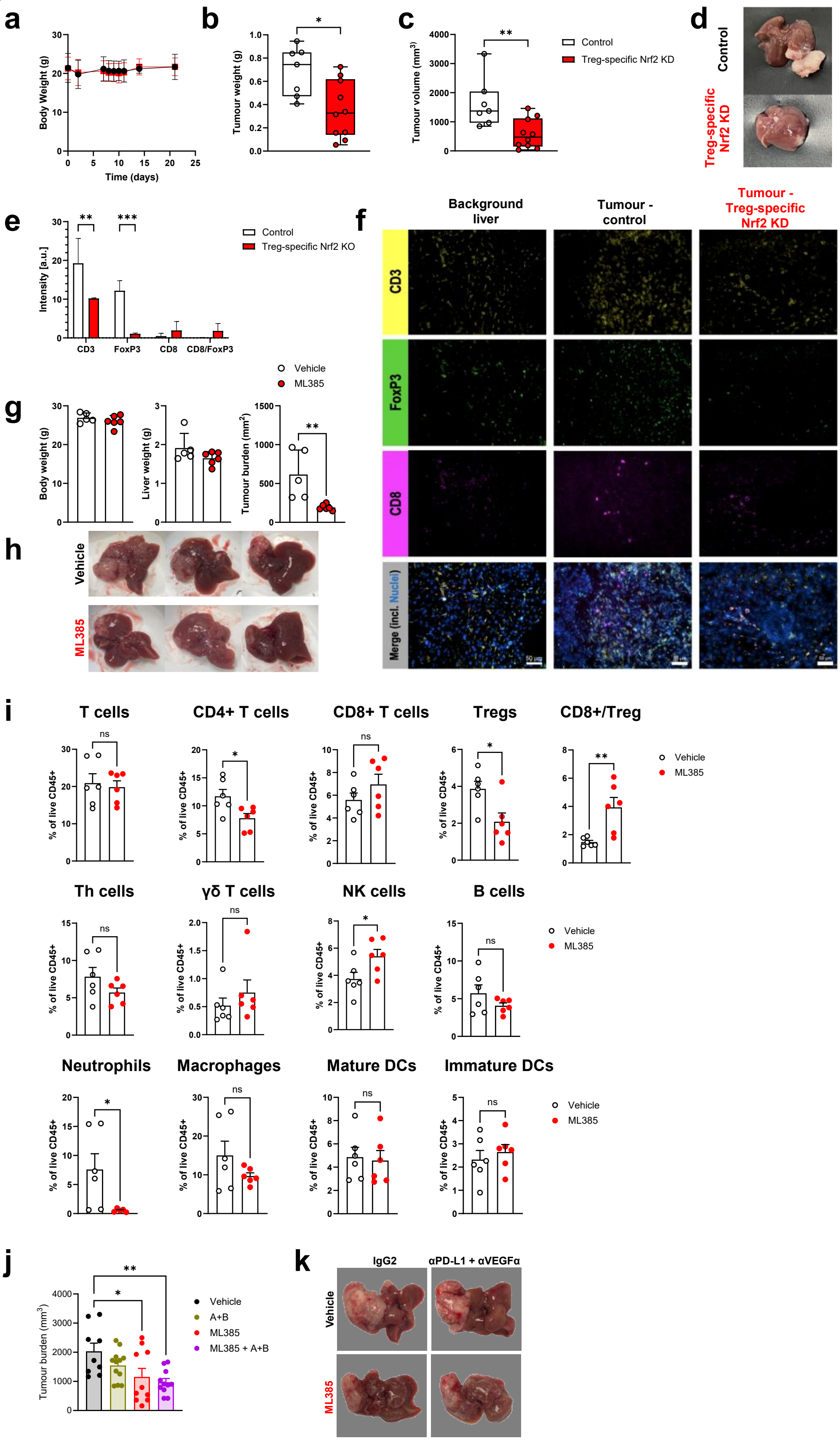
a
Body Weight (g)
Time (days)
b
Tumour weight (g)
c
Tumour volume (mm3)
Control
Treg-specific Nrf2 KD
d
Control
Treg-specific Nrf2 KD
e
Intensity [a.u.]
Control
Treg-specific Nrf2 KO
CD3
FoxP3
CD8
CD8/FoxP3
f
Background liver
Tumour - control
Tumour - Treg-specific Nrf2 KD
CD3
FoxP3
CD8
Merge (incl. Nuclei)
g
Vehicle
ML385
Body weight (g)
Liver weight (g)
Tumour burden (mm2)
h
Vehicle
ML385
i
T cells
CD4+ T cells
CD8+ T cells
Tregs
CD8+/Treg
Th cells
γδ T cells
NK cells
B cells
Neutrophils
Macrophages
Mature DCs
Immature DCs
% of live CD45+
Vehicle
ML385
j
Tumour burden (mm3)
Vehicle
A+B
ML385
ML385 + A+B
k
IgG2
αPD-L1 + αVEGFα
Vehicle
ML385

11) *"Histology analysis on mice fed with western diet should be provided to confirm MAFLD conditions in the liver".*

We apologize for this omission. Our collaborator Dr Jack Leslie, who supervised the HCC murine experiments included in our study, has already reported that western diet consistently results in MAFLD conditions (Leslie J et al. Gut 2022). The corresponding reference has been added to the amended manuscript. Furthermore, representative micrographs of liver tissue stained for histopathological evaluation and the MASH scores have been included as **Supplementary Fig. 18** and **Supplementary Table 15**.

Supplementary Fig. 18:

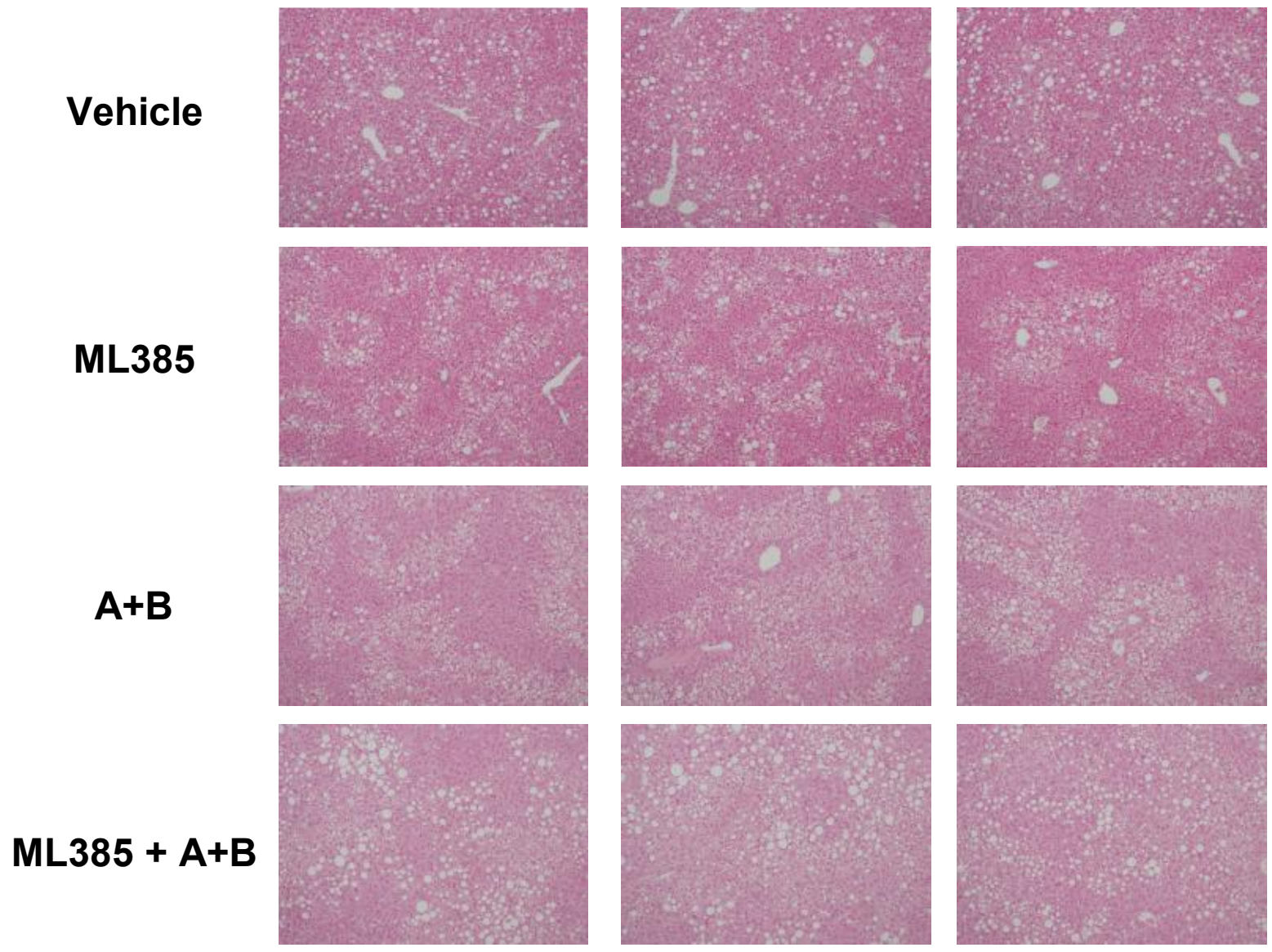

Supplementary Table 15:

Group	Mouse ID	Steatosis	Inflammation	Ballooning	Total
Vehicle	ER410	2	1	0	3
Vehicle	ER411	2	2	0	4
Vehicle	ER412	2	1	0	3
Vehicle	ER413	3	2	0	5
Vehicle	ER414	2	1	0	3
Vehicle	ER449	1	1	0	2
Vehicle	ER450	2	1	0	3
Vehicle	ER451	1	1	0	2
Vehicle	ER452	1	2	0	3
ML385	ER415	2	1	0	3
ML385	ER416	2	2	0	4
ML385	ER417	1	1	0	2
ML385	ER418	2	1	0	3
ML385	ER419	1	2	0	3
ML385	ER420	2	1	0	3
ML385	ER421	2	1	0	3

ML385	ER422	2	1	0	3
ML385	ER423	2	2	0	4
ML385	ER424	1	1	0	2
A+B	ER425	2	1	0	3
A+B	ER426	2	1	0	3
A+B	ER427	2	1	0	3
A+B	ER428	2	1	0	3
A+B	ER429	2	1	0	3
A+B	ER430	2	1	0	3
A+B	ER431	2	1	0	3
A+B	ER432	3	1	0	4
A+B	ER434	3	1	0	4
A+B	ER435	2	1	0	3
A+B	ER436	2	1	0	3
ML385 + A+B	ER437	1	1	0	2
ML385 + A+B	ER438	3	2	0	5
ML385 + A+B	ER439	2	1	0	3
ML385 + A+B	ER441	2	1	0	3
ML385 + A+B	ER442	2	2	0	4
ML385 + A+B	ER443	2	1	0	3
ML385 + A+B	ER444	3	2	0	5
ML385 + A+B	ER445	3	1	0	4
ML385 + A+B	ER446	1	1	0	2
ML385 + A+B	ER447	1	2	0	3
ML385 + A+B	ER448	2	1	0	3

We would like to express our gratitude once more to the Editorial Office and the external reviewers for the very constructive input received. We hope our manuscript will now be acceptable for publication in *Nature Communications*.

Sincerely,

N.Safinia

A.Sanchez-Fueyo
